# Preparation of Alkyl *N*‑Alkylphosphonamidates by the Microwave-Assisted Selective Monoaminolysis of Dialkyl Phosphites

**DOI:** 10.1021/acs.orglett.6c03484

**Published:** 2026-09-09

**Authors:** Zsuzsanna Szalai, Anna Sára Kis, Péter Keglevich, György Keglevich

**Affiliations:** Department of Organic Chemistry and Technology, Faculty of Chemical Technology and Biotechnology, 61810 Budapest University of Technology and Economics, Műegyetem rkp. 3, 1111 Budapest, Hungary

## Abstract

The title compounds, (RO)­(NHR′)­P­(O)­H, were synthesized earlier from *P*-chloro intermediates by substitutions or from dialkyl phosphites by reaction with organometallic reagents. Instead of these not clear-cut and not efficient transformations, a green, solvent- and catalyst-free synthesis of alkyl phosphonamidates has been developed by the microwave-assisted aminolysis of dialkyl phosphites. The monosubstitution was fully selective. Under more forcing conditions, an interesting side reaction was observed. The resulting products may be valuable reagents to prepare α-hydroxy- and α-aminophosphonic derivatives.

## Graphical abstract

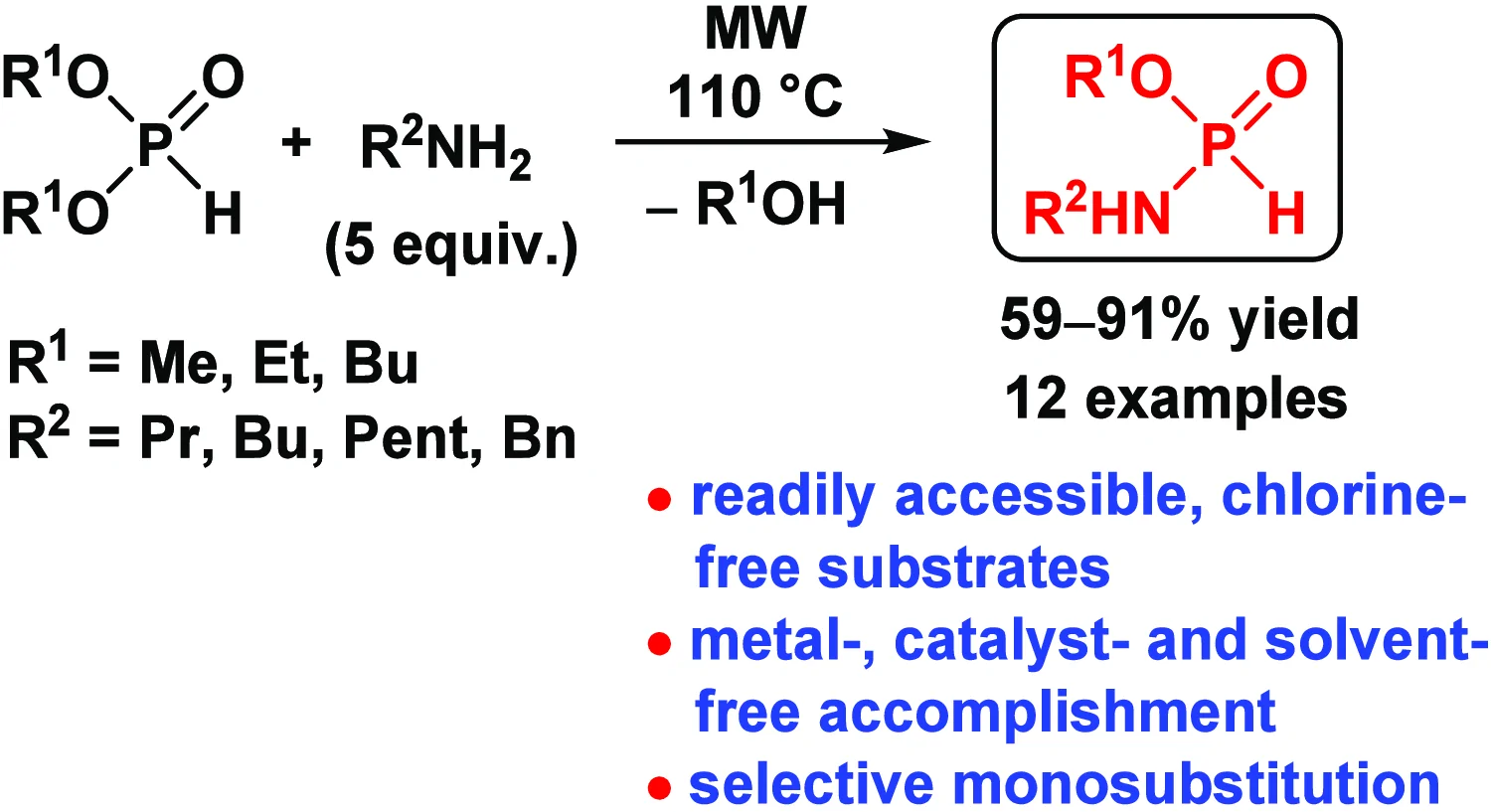

Dialkyl phosphites [(RO)_2_P­(O)­H] are valuable reagents in the synthesis of α-hydroxyphosphonates^1^ and α-aminophosphonates,^2^ and prepared by the reaction of phosphorus trichloride and 3 equiv of the appropriate alcohol in the absence of a base.^3^ At the same time, diaminophosphorous acids [(R_2_N)_2_P­(O)­H] are applied more rarely and are most often synthesized by the selective hydrolysis/fission of phosphorous trisamide^4^ or by the loss of isobutylene from *tert*-butyl tetraethylphosphorodiamidite.^5^ Both starting materials were obtained from phosphorus trichloride. The >P­(O)H species with an alkoxy and an amino substituent [(RO)­(NR′_2_)­P­(O)­H] are special reagents available from either Cl_2_POR^6,7^ or Cl_2_PNR′_2_ ^7,8^ by reaction with an amine or alcohol, respectively, followed by hydrolysis/fission, as shown in Figure 1, marked by “route A” and “route B”, respectively. These are also approaches based on phosphorus trichloride. “Route C” suggests a special method for the preparation of alkyl phosphonamidates suggested by Modro et al., making use of the reaction of diethyl phosphite and a coordination compound, such as tetrakis­(diisopropylamino)­titanium­(IV)^9^ or bis­(diethylamino)­mangan­(II),^10^ or the analogous butylamino derivatives of tin­(II) and tin­(V).^11^ Mostly, this transfer of the dialkylamino group was not a clear-cut reaction. Moreover, the need for organometallic reagents is a disadvantage. A less studied but good option for the preparation of alkyl phosphonamidates involves the alcoholysis of bis­(dialkylamino)­phosphorous acid. Four products were reported in the early literature; however, they were not characterized by spectral data.^12^ (Figure 1, route D). A problem is that the synthesis of the starting diamides has not been elaborated.

**Figure 1:**
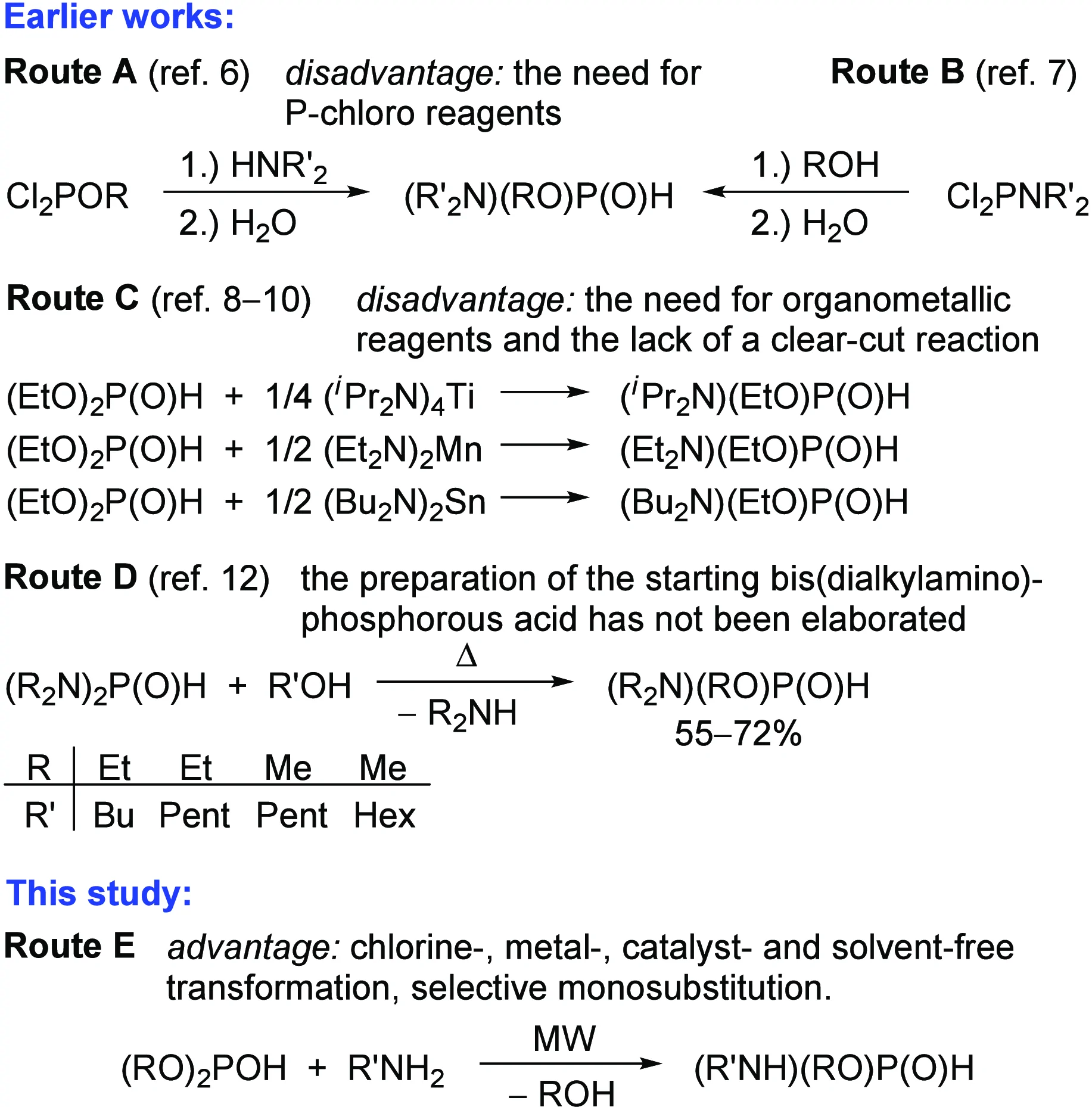
Synthesis strategies for the preparation of alkyl phosphonamidates (R_2_N)­(R′O)­P­(O)­H.

We wished to develop a chlorine-, metal-, and catalyst-free as well as solvent-free microwave (MW)-assisted method for the synthesis of (RO)­(NHR′)­P­(O)H phosphonamidates by the aminolysis of the easily available dialkyl phosphites (Figure 1, route E).

In an earlier study, the MW-assisted transesterification of dialkyl phosphites [(RO_2_)­P­(O)­H] was investigated by the senior author of this letter with co-workers. The ratio of the intermediate with two different alkoxy groups [(RO)­(R′O)­P­(O)­H] and the fully transesterified product [(R′O)_2_P­(O)­H] depended on the excess of alcohol, the temperature, and the time of irradiation,^13^ but it was not possible to obtain the (RO)­(R′O)­P­(O)­H intermediate as the exclusive or even predominating product (Scheme 1).

**Scheme 1:**
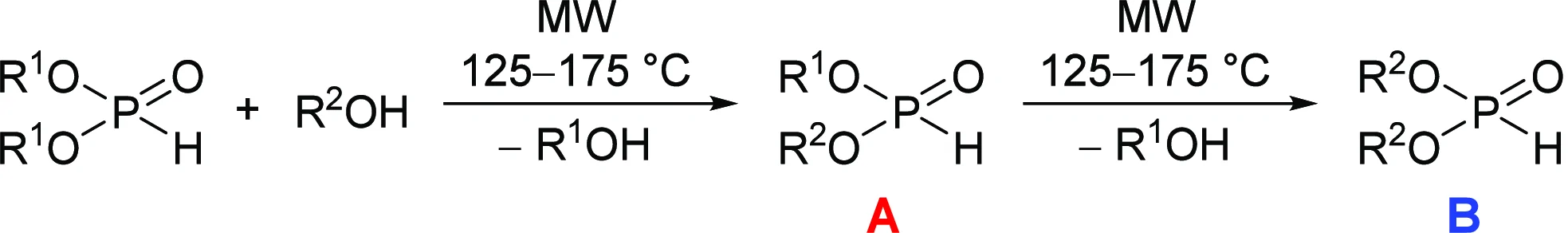
Alcoholysis of Dialkyl Phosphites^13^

However, acyclic and cyclic primary, secondary, and tertiary alcohols incorporating a carbohydrate, a steroid, or an amino acid scaffold could be converted selectively to the corresponding monosubstituted derivative of dimethyl phosphite in the presence of either Zn­(acac)_2_ or bis­(2,2,6,6-tetramethyl-3,5-heptanedionato)­zinc­(II) catalyst under mild conditions.^14^ Obviously, the steric hindrance may be the reason for the selectivity. At the same time, the optical resolution of dialkyl phosphites with different alkyl groups has not yet been elaborated.

We hoped that, during the aminolysis of dialkyl phosphites with alkylamines, a selective monosubstitution will be possible. As another precedent, the MW-promoted aminolysis of alkyl diphenylphosphinates and 1-alkoxy-3-methyl-3-phospholene 1-oxides was investigated by us.^15^ In the course of our work supported by refs 13 and 15 , MW irradiation ensured fast, selective, and efficient transformations due to the beneficial effect of the statistically occurring local overheatings.^16^

Our aim was to elaborate a simple and “green” method for the preparation of (RO)­(NHR′)­P­(O)H species by the MW-assisted condensation of dialkyl phosphites and primary amines (Figure 1, route E).

It was a surprising observation during the preliminary experiments that the reaction of dialkyl phosphites and primary amines furnished monosubstituted product (RO)­(R′NH)­P­(O)H in complete selectivity. The reaction conditions were then optimized via the condensation of diethyl phosphite and butylamine. The experimental data were collected in Table 1. One can see that the conversion of the aminolysis performed at 120 °C with an irradiation time of 4 h applying the amine in a 1 equiv quantity was 85%. Complete conversions could be obtained at 110 °C after a reaction time of 4.5 and 3 h using 3 and 5 equiv of butylamine, respectively. In the latter two cases, the isolated yield of (EtO)­(BuNH)­P­(O)­H (**2b**) was 79 and 91%, respectively. The work-up included removal of the excess of the amine and purification by flash column chromatography.

**Table 1:**
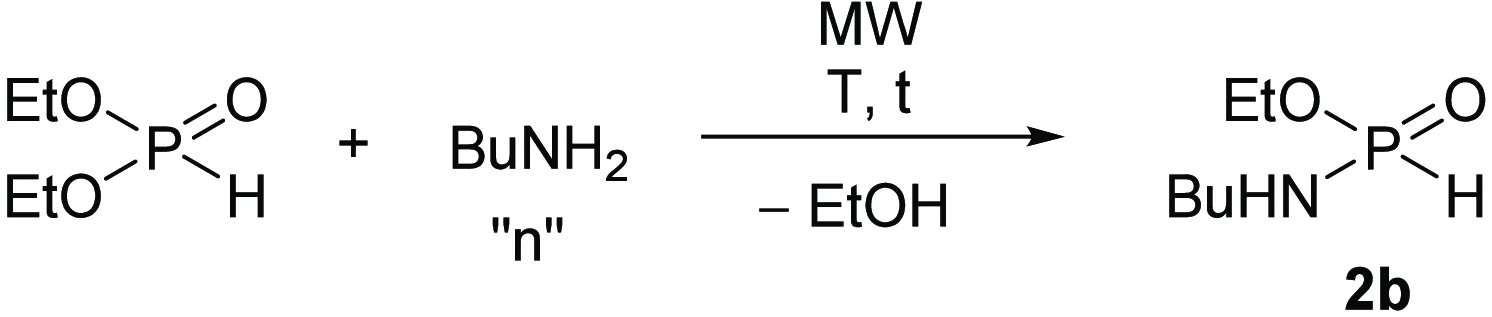
Optimization of the Reaction Conditions

*n* (equiv)	*T* (°C)	*t* (h)	conversion (%)^a^	yield (%)
1	120	4	85	
3	110	4.5	100	79
5	110	3	100	91
5	110^b^	3	83	

a On the basis of relative ^31^P NMR intensities.

b Comparative thermal experiments.

A comparative thermal experiment afforded target product **2b** in a lower conversion of 83%, highlighting the potential of MWs.

Then, the optimized conditions comprising MW irradiation at 110 °C for 3 h applying the alkylamines (propylamine, butylamine, and pentylamine) in a 5-fold quantity were utilized in the aminolysis of dimethyl phosphite, diethyl phosphite, and tributyl phosphite (Scheme 2). The alkyl phosphonamidates **1a**–**1c**, **2a**–**2c**, and **3a**–**3c** were obtained as oils in yields of 59–91% after removing the excess of the amine in vacuum and purifying the crude products so obtained by flash column chromatography. ^31^P NMR analysis of the crude mixtures suggested a completely selective aminolysis. No disubstitution took place, not even in the presence of 5-fold excess of the alkylamines. This is especially noteworthy if the outcome of the aminolyzes under discussion is compared to that of the alcoholyses of dialkyl phosphites mentioned above.^13^ The mechanism comprises the nucleophilic attack of the primary amine on the P atom of the P=O function pentavalent tetracoordinated *H*-phosphonate. In the first step, a pentacoordinated adduct is formed that may undergo elimination of the alcohol molecule following pseudorotations navigating the system to an energy minimum.^17,18^ The reasons for the selectivity will be explored by quantum chemical calculations in due course. With the change from alkylamines to benzylamine as the nucleophile, only 1 equiv of this high-boiling-point reagent was applied in order to simplify the work-up. As a compensation, a longer irradiation period of 6 h was necessary. In this way, three additional alkyl phosphonamidates **1d**, **2d**, and **3d** could be obtained. The yields were somewhat more moderate (60–70%).

**Scheme 2:**
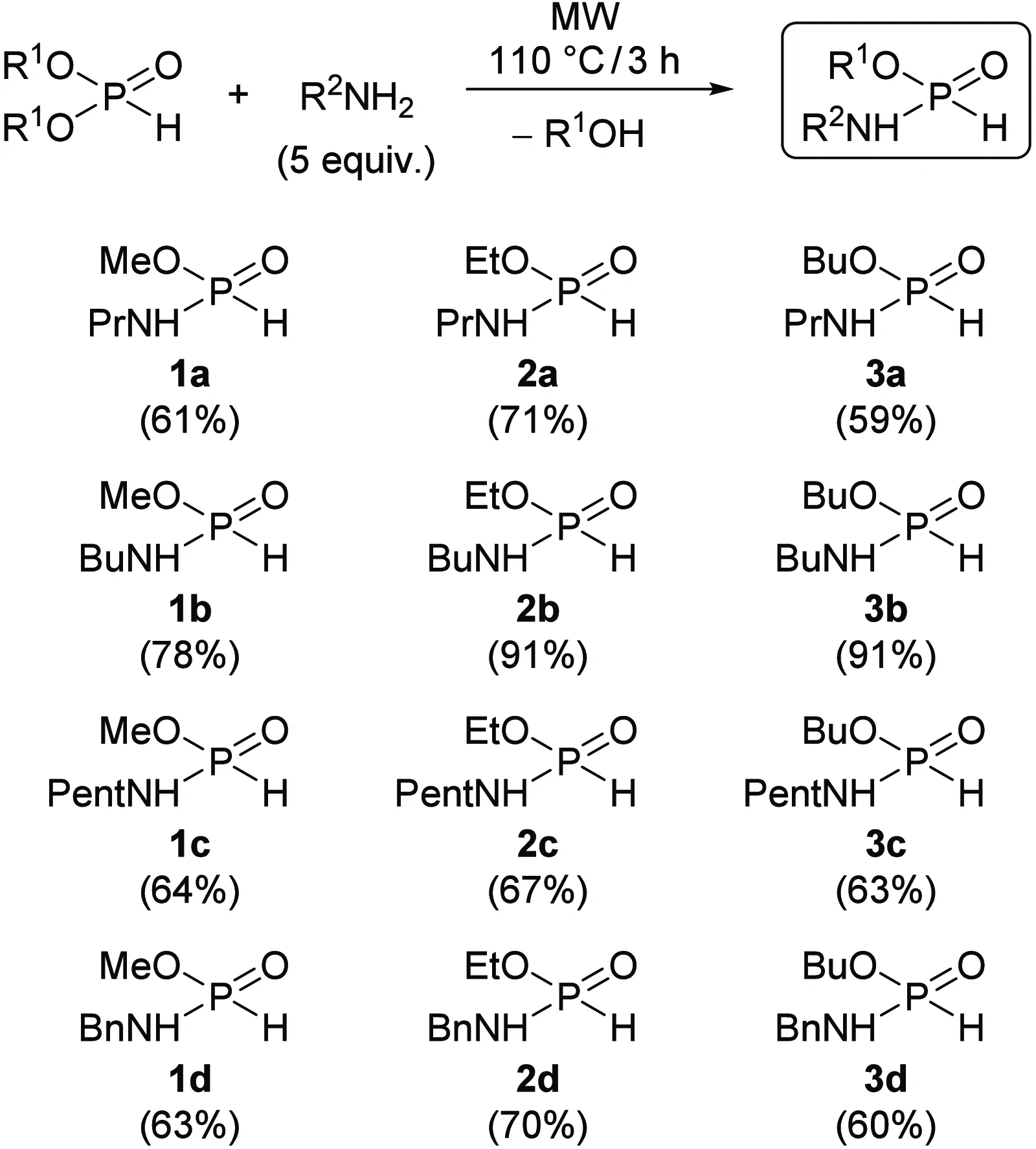
MW-Assisted Aminolysis of Dialkyl Phosphites

New alkyl phosphonamidates **1**–**3**/**a**–**d** were fully characterized by ^31^P, ^13^C and ^1^H NMR data. The ^1^ *J*_PH_ couplings of 613–628 Hz were obtained from the ^1^H NMR spectra and were confirmed by data from the proton-coupled ^31^P NMR spectra (Table 2). No doubt remained regarding the P­(O)H function in products **1**–**3**. The NMR data, such as the δ_C_ and δ_H_ chemical shifts along with *J*_PC_ and *J*_PH_ couplings, confirmed the presence of the NH-alkyl and O-alkyl substituents. However, the molecular ions did not appear in analyses under protonating soft-ionization (ESI) mass spectral methods. This phenomenon was attributed to the lability of the P–N bond: protonation may promote its solvolytic cleavage. To get further evidence, IR spectra of phosphonamidates **1**–**3** were also recorded. The ν_P=O_ and ν_P–O–C_ stretching vibrations in the range of 1201–1209 and 1054–1064 cm^–1^, respectively, were characteristic.

**Table 2:** ^1^ *J*_PH_ Couplings Observed in Alkyl Phosphonamidates **1**–**3**/**a**–**d**

compound	^1^ *J*_PH_ (Hz) from the ^1^H NMR spectra	^1^ *J*_PH_ (Hz) from the H-coupled ^31^P NMR spectra
**1a**	627.5	625.6
**1b**	622.8	624.2
**1c**	615.7	618.0
**1d**	623.8	621.2
**2a**	621.8	618.7
**2b**	625.8	623.2
**2c**	613.2	615.3
**2d**	620.2	618.1
**3a**	615.4	616.6
**3b**	615.0	612.1
**3c**	613.5	616.1
**3d**	621.4	619.3

Going further, 1 equiv of α-phenyl-ethylamine was also reacted with diethyl phosphite under MW irradiation at 110 °C for 6 h. The substitution was significantly slower, as the conversion was only 49%. Besides product (EtO)­[NHCH­(Ph)­Et]­P­(O)H **2e** [δ_P_ (CDCl_3_) 3.0], some decomposition also took place according to the ^31^P NMR spectrum of the crude mixture. Applying 135 °C for 3.5 h, the reaction mixture became quite complex. It means that primary amines with a branched substituent are not so reactive in the transformation under discussion. This is obvious if the steric hindrance is considered. In the next stage of our work aiming at possible extensions, we shall try to find the appropriate conditions to use less reactive amines, including secondary amines and optically active amines.

The excess of the alkylamines obtained on the concentration of the reaction mixtures could be re-used in subsequent experiments.

The chemoselective aminolysis of dialkyl phosphites with primary alkylamines developed by us seems to be of a more general value and will be investigated in detail to evaluate the possible extensions as well as the scope and limitations.

It was interesting to find that, carrying out the condensation of dibutyl phosphite with 5 equiv of butylamine at a somewhat higher temperature of 135 °C for 3 h, an unexpected product identified as (BuO)­(BuNH)­P­(NBu)H (**5**) was also present in 45% in the crude mixture beyond the 55% of expected product **3b**. If its tautomer is named, **5** is the monoester diamide derivative of phosphorous acid that was isolated in a pure form in a 32% yield by column chromatography.

The formation of unexpected product **5** was explained by assuming tautomerism of phosphonamidate **3b** to form **4**, followed by the condensation of the latter with a second molecule of the amine under the conditions of the reaction, leading to product **5** existing under tautomeric form **5′** (Scheme 3).

**Scheme 3:**
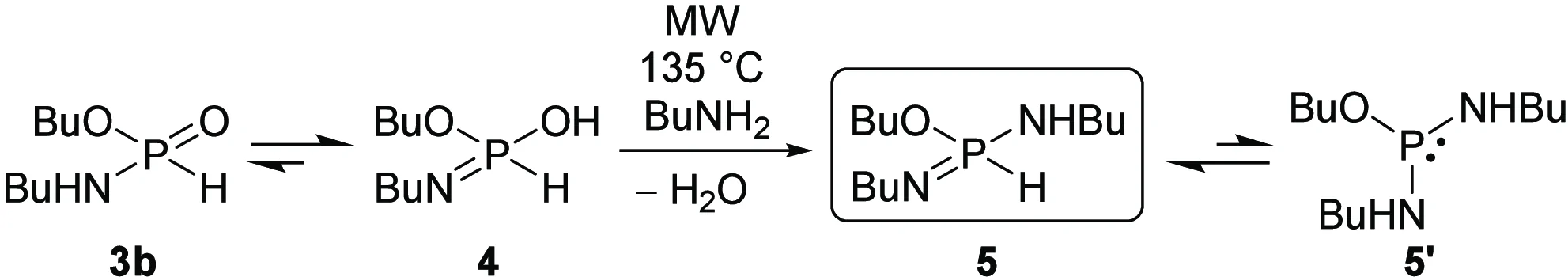
Formation of the Butyl Bis­(butylamino)­phosphorous Acid (**5**) Byproduct under More Forcing Conditions

A similar side reaction also took place with propylamine under the conditions applied above to furnish a species in which, instead of a propyl substituent, there was a butyl group, as in (BuO)­(BuNH)­P­(NPr)­H (**6**). The ratio of components **3a** and **6** was 1:1 in the crude mixture. The butylamino-propylamino derivative (**6**) was isolated in 39% yield. The change of the alkyl group may have occurred due to the effect of the butyl alcohol formed in the first step of the reaction sequence.

Structures of the phosphorous acid ester diamides **5** and **6** were supported by ^31^P, ^13^C, and ^1^H NMR spectral data. IR absorption for the P=N unit appeared at 1206 cm^–1^ in accordance with literature data.^19^ The unexpected transformation could not be observed applying benzylamine in condensation with dibutyl phosphite.

It was observed that, allowing the MW irradiation of the mixture of dibutyl phosphite and 3 equiv of propylamine at 110 °C for 6 h, there was a minor component in the crude mixture besides the expected product (**3a**). After work-up and identification, the byproduct was found to be butyl *N*-butyl-*N*-propyl phosphonamidate (**7**) obtained in 23% yield from the crude mixture (Scheme 4). The butyl-propylamino derivative (**7**) was fully characterized. Its formation was explained by assuming that primarily formed butyl phosphonamidate **3a** was alkylated under the conditions of the reaction by butyl alcohol that was the byproduct in the first condensation step (Scheme 4). In this letter, the side reactions including the N-alkylation were not investigated in detail.

**Scheme 4:**
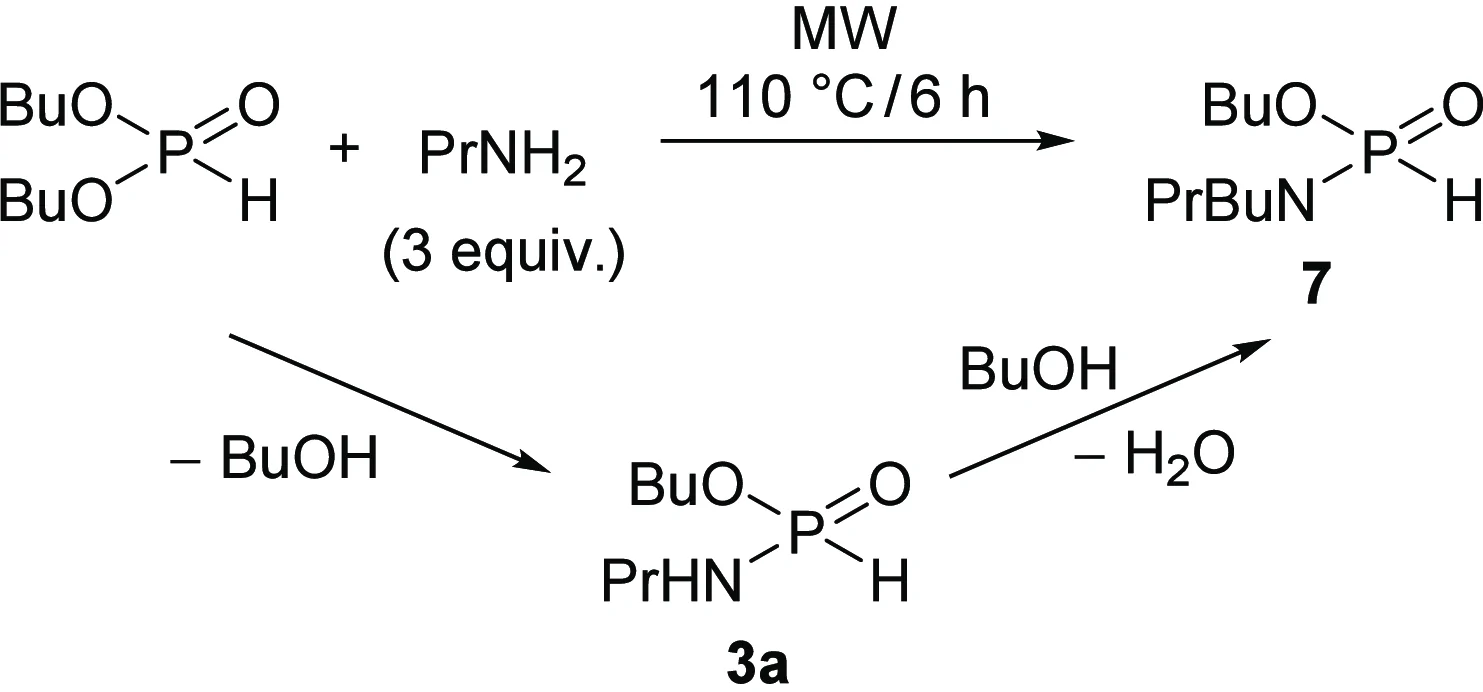
Formation of the N-Butylated Byproduct (**7**) in the Aminolysis of Dibutyl Phosphite with Propylamine

In conclusion, the chemoselective aminolysis of easily available dialkyl phosphites with primary alkylamines affording alkyl phosphonamidates was developed. Optimum conditions of this transformation involved MW irradiation of the 1:5 mixture of the phosphite and the primary amine at 110 °C for 3 h. The work-up comprised removal of the excess of the amine and flash column chromatography. The method developed seems to be of a more general nature and more advantageous than those described in the literature starting from *P*-chloro species or applying organometallic reagents and facing selectivity problems. Three side reactions were also identified that may orient us to fine tune the transformations found.

## Supplementary Material



## Data Availability

The data underlying this study are available in the published article and its Supporting Information.

## Notes

Supplementary text (experimental) and ^31^P, ^13^C, and ^1^H NMR spectra (PDF)

## References

[1] d Rádai, Z. ; Kiss, N. Z. ; Keglevich, G. In Organophosphorus Chemistry: Novel Developments; Keglevich, G. , Ed.; Walter de Gruyter: Berlin, Germany, 2018; Chapter 5, pp 91–107, 10.1515/9783110535839-005.

[2] a Kukhar, V. P. ; Hudson, H. R. Aminophosphonic and Aminophosphinic Acids: Chemistry and Biological Activity; John Wiley & Sons: Chichester, U.K., 2000; ISBN: 0471891495.

[3] Quin, L. D. A Guide to Organophosphorus Chemistry; Wiley & Sons: New York, 2000; ISBN: 978-0-471-31824-8.

[4] Clement J.-L., Frejaville C., Tordo P. (2002). DEPMPO and lipophilic
analogs: synthesis and EPR studies. Res. Chem.
Intermediat..

[5] Gazizov T. Kh., Sal’keeva L. K., Gafurov E. K., Kazantsev A. V. (1988). Mechanism
of the reaction of phosphorous ester amides with protic acids. Zh. Obshch. Khim..

[6] Foss V. L., Lukashev N. V., Borisenko A. A., Lutsenko I. F. (1980). Anhydrides of *O*-alkyldialkylamidophosphorous
acids and monoxides isomeric to them. Phosphorotropic tautomerism. Zh. Obshch. Khim..

[7] Zwierzak A., Koziara A. (1967). Phosphorous acid amides. II. Synthesis of monoalkyl
phosphoro amidites (RO) (R_2_′N)­P­(O)­H. Tetrahedron.

[8] Sal’keeva L. K., Nurmagambetova M. T., Kurmanaliev O. Sh., Gazizov T. Kh. (2003). Reactions of tert-Butyl Tetraethylphosphorodiamidite
with Acetic and Trifluoroacetic Acids. Russ.
J. Gen. Chem..

[9] Froneman M., Cheney D. L., Modro T. A. (1990). Dialkylamino group transfer from
titanium­(IV) to a phosphoryl center. Structure-reactivity studies. Phosphorus, Sulfur, Silicon.

[10] Fester V. D., Vather S. M., Modro T. A. (1987). The reactivity
of manganese diethylamide
with phosphate triesters. Phosphorus, Sulfur,
Silicon.

[11] Froneman M., Modro T. A., Vather S. M. (1989). Dialkylamido derivatives of tin­(II)
and tin­(IV) in phosphorus chemistry. Inorg.
Chim. Acta.

[12] Nifant’ev E. E., Shilov I. V. (1972). Acid amides of phosphorous acid as phosphorylating
reagents. Zh. Obshch. Khim..

[13] Bálint E., Tajti Á., Drahos L., Ilia G., Keglevich G. (2013). Alcoholysis of dialkyl phosphites
under microwave conditions. Curr. Org. Chem..

[14] Saito Y., Cho S. M., Danieli L. A., Kobayashi S. (2020). Zinc-catalyzed
phosphonylation of alcohols with alkyl phosphites. Org. Lett..

[15] Keglevich G., Harsági N., Szöllősi B., Drahos L. (2024). Chlorine-free synthesis
of phosphinic derivatives by change in the P-function. Green Proc. Synth..

[16] Keglevich G., Kiss N. Z., Mucsi Z., Körtvélyesi T. (2012). Insights into
a surprising reaction: The microwave-assisted direct esterification
of phosphinic acids. Org. Biomol. Chem..

[17] Kolodiazhnyi O. I., Kolodiazhna A. (2017). Nucleophilic
substitution at phosphorus: stereochemistry
and mechanisms. Tetrahedron: Asymmetry.

[18] Couzijn E. P. A., Slootweg J. C., Ehlers A. W., Lammertsma K. (2010). Stereomutation
of pentavalent compounds: validating the Berry pseudorotation, redressing
Ugi’s turnstile rotation, and revealing the two- and three-arm
turnstiles. J. Am. Chem. Soc..

[19] Jiang S., Yu G.-W., Li Z.-G., Chu S.-P., Wang S.-P. (2015). Synthesis
and characterization of phosphazene derivatives containing dioxybiphenyl
and 4-sulfanylquinazoline groups. J. Chem. Res..

