# Supplementary material for: Preparation of Alkyl N‑Alkylphosphonamidates by the Microwave-Assisted Selective Monoaminolysis of Dialkyl Phosphites
Source: Org Lett. 2026 Sep 9;28(37):11784–8. doi: 10.1021/acs.orglett.6c03484 (PMC13595902; doi:10.1021/acs.orglett.6c03484)

Supplementary Information

Preparation of Alkyl N-Alkylphosphonamidates by the Microwave-Assisted Selective Monoaminolysis of Dialkyl Phosphites

Zsuzsanna Szalai, Anna Sára Kis, Péter Keglevich, and György Keglevich*

Department of Organic Chemistry and Technology, Faculty of Chemical Technology and Biotechnology, Budapest University of Technology and Economics, 1111 Budapest, Műegyetem rkp. 3, Hungary, E-mail: keglevich.gyorgy@vbk.bme.hu

Table of Contents

1. Experimental 2
  1.1. General Information 2
  1.2. General Procedure for the Synthesis of Alkyl *N*-alkylphosphonamidates 2
  1.3. General Procedure for the Synthesis of Butyl *N,N'*-dibutylphosphonamid-imidate (**5**) and Butyl *N*-butyl-*N'*-propylphosphonamid-imidate (**6**) 6
  1.4. Synthesis of butyl *N*-butyl-*N*-propylphosphonamidate (**7**) 7
2. ^{31}P, ^{13}C, ^{1}H NMR spectra of the compounds **1a-d**, **2a-d**, **3a-d**, **5**, **6** and **7** synthesized 8

1. Experimental

1.1. General Information

The MW reactions were carried out in a CEM Discover (300 W) focused microwave reactor (CEM Microwave Technology Ltd., Buckingham, UK) equipped with a stirrer and a pressure controller using 80–100 W irradiation under isothermal conditions. The reaction mixtures were irradiated in sealed glass vessels (with a volume of 10 mL) available from the supplier of CEM. The reaction temperature was monitored by an external IR sensor.

The ^{31}P, ^{13}C, and ^{1}H NMR spectra were recorded on a Bruker DRX-500 or Bruker Avance-300 spectrometer (Bruker, Billerica, MA, USA) operating at 202, 126, and 500 MHz or 122, 75, and 300 MHz, respectively.

HRMS data could not be obtained, as the molecular ions did not appear in analyses under protonating soft-ionization (ESI) mass spectral methods. Under the conditions applied, the alkyl phosphonamidates **1a-d**, **2a-d** and **3a-d,** as well as derivatives **5**-**7** probably suffered solvolytic cleavage of the P–N bond.

1.2. General Procedure for the Synthesis of Alkyl *N*-alkylphosphonamidates

A mixture of 2.0 mmol of dialkyl phosphite (dimethyl phosphite: 0.18 mL, diethyl phosphite: 0.26 mL, dibutyl phosphite: 0.39 mL) and 10.0 mmol of the alkylamine (***A***) (propylamine: 0.82 mL, butylamine: 0.99 mL, pentylamine: 1.16 mL) or 2 mmol (0.22 mL) of benzylamine (***B***) was heated at 110°C in a vial in a CEM Discover Microwave reactor for 3h (***A***) or 6h (***B***). The crude products obtained after removing the volatile components in vacuum were purified by flash column chromatography on a 10 cm silica gel layer applying dichloromethane – methyl alcohol 97:3 → 9:1 → 1:1 mixtures as specified below. Products **1a-d**, **2a-d** and **3a-d** obtained were characterized below:

Methyl N-propylphosphonamidate (**1a**): eluent: dichloromethane – methyl alcohol 97:3 → 9:1 → 1:1 mixtures used in volumes of 150 ml – 250 ml – 50 ml, respectively; Yield: 0.17 g (61%)

as a colorless oil; ^{31}P {^{1}H} NMR (202 MHz, $CDCl_3$) δ 6.2; ^{13}C {^{1}H} NMR (126 MHz, $CDCl_3$) δ 50.8 (d, J = 4.1 Hz, OCH_3), 40.8 (s, NCH_2), 21.1 (s, CH_2CH_3), 11.1 (s, CH_2CH_3); ^{1}H NMR (500 MHz, $CDCl_3$) δ 6.73 (d, J = 627.5 Hz, 1H), 3.96 (bs, 1H), 3.59 (d, J = 12.1 Hz, 3H), 2.92–2.80 (m, 2H), 1.79–1.72 (m, 2H), 1.02 (t, J = 7.4 Hz, 3H).

Methyl N-butylphosphonamidate (**1b**): eluent: dichloromethane – methyl alcohol 97:3 → 9:1 → 1:1 mixtures used in volumes of 150 ml – 250 ml – 50 ml, respectively; Yield: 0.24 g (78%) as a colorless oil; ^{31}P {^{1}H} NMR (122 MHz, $CDCl_3$) δ 6.3; ^{13}C {^{1}H} NMR (75 MHz, $CDCl_3$) δ 50.8 (d, J = 4.4 Hz, OCH_3), 38.9 (s, NCH_2), 29.7 (s, NCH_2CH_2), 19.8 (s, CH_2CH_3), 13.5 (s, CH_2CH_3); ^{1}H NMR (300 MHz, $CDCl_3$) δ 6.75 (d, J = 622.8 Hz, 1H), 3.58 (d, J = 12.0 Hz, 3H), 2.91–2.70 (m, 3H), 1.74–1.64 (m, 2H), 1.47–1.35 (m, 2H), 0.95 (t, J = 7.3 Hz, 3H).

Methyl N-pentylphosphonamidate (**1c**): eluent: dichloromethane – methyl alcohol 97:3 → 9:1 → 1:1 mixtures used in volumes of 150 ml – 250 ml – 50 ml, respectively; Yield: 0.21 g (64%) as a colorless oil; ^{31}P {^{1}H} NMR (202 MHz, $CDCl_3$) δ 6.3; ^{13}C {^{1}H} NMR (75 MHz, $CDCl_3$) δ 50.8 (d, J = 4.4 Hz, OCH_3), 39.2 (s, NCH_2), 28.7 (s, NCH_2CH_2), 27.5 (s, $CH_2CH_2CH_3$), 22.1 (s, CH_2CH_3), 13.8 (s, CH_2CH_3); ^{1}H NMR (300 MHz, $CDCl_3$) δ 6.72 (d, J = 615.7 Hz, 1H), 3.55 (d, J = 12.0 Hz, 3H), 2.86–2.80 (m, 3H), 1.79–1.64 (m, 2H), 1.42–1.27 (m, 4H), 0.91 (t, J = 6.8 Hz, 3H).

Methyl N-benzylphosphonamidate (**1d**): eluent: dichloromethane – methyl alcohol 97:3 → 9:1 → 1:1 mixtures used in volumes of 150 ml – 250 ml – 50 ml, respectively; Yield: 0.23 g (63%) as a colorless oil; ^{31}P {^{1}H} NMR (122 MHz, $CDCl_3$) δ 5.6; ^{13}C {^{1}H} NMR (75 MHz, $CDCl_3$) δ 133.9 (C_α), 129.0 (C_γ^*), 128.6 (C_β^*), 128.3 (s, C_δ), 50.6 (d, J = 3.3 Hz, OCH_3), 42.9 (s, NCH_2), *may be reversed; ^{1}H NMR (500 MHz, $CDCl_3$) δ 7.42–7.24 (m, 5H), 6.53 (d, J = 623.8 Hz, 1H), 4.24 (bs, 1H), 3.93 (s, 2H), 3.37 (d, J = 11.3 Hz, 3H).

Ethyl N-propylphosphonamidate (**2a**): eluent: dichloromethane – methyl alcohol 97:3 → 9:1 → 1:1 mixtures used in volumes of 150 ml – 250 ml – 50 ml, respectively; Yield: 0.21 g (71%) as

a colorless oil; $^{31}P\ \{^1H\}$ NMR (202 MHz, $CDCl_3$) δ 3.9; $^{13}C\ \{^1H\}$ NMR (75 MHz, $CDCl_3$) δ 59.6 (d, *J* = 4.3 Hz, OCH_2), 40.8 (s, NCH_2), 21.1 (s, $\mathit{C}H_2CH_3$), 16.5 (d, *J* = 7.1 Hz, $OCH_2\mathit{C}H_3$), 11.1 (s, $CH_2\mathit{C}H_3$); ^1H NMR (300 MHz, $CDCl_3$) δ 6.78 (d, *J* = 621.8 Hz, 1H), 3.99–3.81 (m, 2H), 2.93–2.65 (m, 3H), 1.87–1.69 (m, 2H), 1.29 (t, *J* = 7.0 Hz, 3H), 1.00 (t, *J* = 7.4 Hz, 3H).

Ethyl N-butylphosphonamidate (**2b**): eluent: dichloromethane – methyl alcohol 97:3 → 9:1 → 1:1 mixtures used in volumes of 150 ml – 250 ml – 50 ml, respectively; Yield: 0.30 g (91%) as a colorless oil; $^{31}P\ \{^1H\}$ NMR (122 MHz, $CDCl_3$) δ 4.0; $^{13}C\ \{^1H\}$ NMR (75 MHz, $CDCl_3$) δ 59.6 (d, *J* = 4.3 Hz, OCH_2), 39.0 (s, NCH_2), 29.8 (s, $NCH_2\mathit{C}H_2$), 19.9 (s, $\mathit{C}H_2CH_3$), 16.5 (d, *J* = 7.1 Hz, $OCH_2\mathit{C}H_3$), 13.5 (s, $CH_2\mathit{C}H_3$); ^1H NMR (300 MHz, $CDCl_3$) δ 6.78 (d, *J* = 625.8 Hz, 1H), 3.98–3.84 (m, 2H), 3.04–2.79 (m, 3H), 1.79–1.65 (m, 2H), 1.49–1.32 (m, 2H), 1.30 (t, J = 7.1 Hz, 3H), 0.96 (t, *J* = 7.3 Hz, 3H).

Ethyl N-pentylphosphonamidate (**2c**): eluent: dichloromethane – methyl alcohol 97:3 → 9:1 → 1:1 mixtures used in volumes of 150 ml – 250 ml – 50 ml, respectively; Yield: 0.24 g (67%) as a colorless oil; $^{31}P\ \{^1H\}$ NMR (122 MHz, $CDCl_3$) δ 4.1; $^{13}C\ \{^1H\}$ NMR (75 MHz, $CDCl_3$) δ 59.7 (d, *J* = 4.3 Hz, OCH_2), 39.4 (s, NCH_2), 28.8 (s, $NCH_2\mathit{C}H_2$), 27.8 (s, $\mathit{C}H_2CH_2CH_3$), 22.2 (s, $\mathit{C}H_2CH_3$), 16.5 (d, *J* = 7.0 Hz, $OCH_2\mathit{C}H_3$), 13.8 (s, $CH_2\mathit{C}H_3$); ^1H NMR (300 MHz, $CDCl_3$) δ 6.77 (d, *J* = 613.2 Hz, 1H), 3.94–3.84 (m, 2H), 2.86–2.81 (m, 3H), 2.38 (bs, 1H), 1.80–1.64 (m, 2H), 1.40–1.31 (m, 4H), 1.28 (t, *J* = 7.1 Hz, 3H), 0.91 (t, *J* = 6.9 Hz, 3H).

Ethyl N-benzylphosphonamidate (**2d**): eluent: dichloromethane – methyl alcohol 97:3 → 9:1 → 1:1 mixtures used in volumes of 150 ml – 250 ml – 50 ml, respectively; Yield: 0.28 g (70%) as a colorless oil; $^{31}P\ \{^1H\}$ NMR (202 MHz, $CDCl_3$) δ 3.9; $^{13}C\ \{^1H\}$ NMR (75 MHz, $CDCl_3$) δ 134.0 (C_α), 128.9 (C_γ^*), 128.7 (C_β^*), 128.4 (C_δ), 59.6 (d, *J* = 4.2 Hz, OCH_2), 42.9 (s, NCH_2), 16.5 (d, *J* = 7.0 Hz, $CH_2\mathit{C}H_3$), *may be reversed; ^1H NMR (500 MHz, $CDCl_3$) δ 7.33–7.27 and 7.43–7.42 (m, 5H), 6.49 (d, *J* = 620.2 Hz, 1H), 3.94 (s, 2H), 3.68–3.62 (m, 2H), 1.11 (t, *J* = 7.1 Hz, 3H).

Butyl N-propylphosphonamidate (**3a**): eluent: dichloromethane – methyl alcohol 97:3 → 9:1 → 1:1 mixtures used in volumes of 150 ml – 250 ml – 50 ml, respectively; Yield: 0.21 g (59%) as a colorless oil; ^{31}P {^{1}H} NMR (122 MHz, $CDCl_3$) δ 4.4; ^{13}C {^{1}H} NMR (75 MHz, $CDCl_3$) δ 63.5 (d, J = 4.4 Hz, OCH_2), 40.8 (s, NCH_2), 32.7 (d, J = 6.9 Hz, $OCH_2\mathit{C}H_2$), 20.9 (s, $\mathit{C}H_2CH_3$), 18.8 (s, $\mathit{C}H_2CH_3$), 13.6 (s, $CH_2\mathit{C}H_3$), 11.0 (s, $CH_2\mathit{C}H_3$); ^{1}H NMR (500 MHz, $CDCl_3$) δ 6.74 (d, J = 615.4 Hz, 1H), 3.82–3.78 (m, 2H), 3.36 (bs, 1H), 2.81–2.78 (m, 2H), 1.77–1.69 (m, 2H), 1.62–1.56 (m, 2H), 1.42–1.34 (m, 2H), 0.98 (t, J = 7.5 Hz, 3H), 0.92 (t, J = 7.4 Hz, 3H).

Butyl N-butylphosphonamidate (**3b**): eluent: dichloromethane – methyl alcohol 97:3 → 9:1 → 1:1 mixtures used in volumes of 150 ml – 250 ml – 50 ml, respectively; Yield: 0.35 g (91%) as a colorless oil; ^{31}P {^{1}H} NMR (202 MHz, $CDCl_3$) δ 4.1; ^{13}C {^{1}H} NMR (126 MHz, $CDCl_3$) δ 63.8 (d, J = 4.2 Hz, OCH_2), 39.0 (s, NCH_2), 32.8 (d, J = 6.9 Hz, $OCH_2\mathit{C}H_2$), 29.7 (s, $NCH_2\mathit{C}H_2$), 19.9 (s, $\mathit{C}H_2CH_3$), 19.0 (s, $\mathit{C}H_2CH_3$), 13.7 (s, $CH_2\mathit{C}H_3$), 13.5 (s, $CH_2\mathit{C}H_3$); ^{1}H NMR (500 MHz, $CDCl_3$) δ 6.73 (d, J = 615.0 Hz, 1H), 3.81–3.77 (m, 2H), 2.82–2.79 (m, 2H), 1.69–1.63 (m, 2H), 1.60–1.55 (m, 2H), 1.40–1.33 (m, 4H), 0.92 (t, J = 7.5 Hz) and 0.89 (t, J = 7.4 Hz) overlapped, total intensity 6H.

Butyl N-pentylphosphonamidate (**3c**): eluent: dichloromethane – methyl alcohol 97:3 → 9:1 → 1:1 mixtures used in volumes of 150 ml – 250 ml – 50 ml, respectively; Yield: 0.26 g (63 %) as a colorless oil; ^{31}P {^{1}H} NMR (202 MHz, $CDCl_3$) δ 4.7; ^{13}C {^{1}H} NMR (126 MHz, $CDCl_3$) δ 63.8 (d, J = 4.5 Hz, OCH_2), 39.2 (s, NCH_2), 32.8 (d, J = 6.9 Hz, $OCH_2\mathit{C}H_2$), 28.8 (s, $NCH_2\mathit{C}H_2$), 27.4 (s, $CH_2\mathit{C}H_2CH_2CH_3$), 22.2 (s, $\mathit{C}H_2CH_3$), 19.0 (s, $\mathit{C}H_2CH_3$), 13.9 (s, $CH_2\mathit{C}H_3$), 13.7 (s, $CH_2\mathit{C}H_3$); ^{1}H NMR (500MHz, $CDCl_3$) δ 6.74 (d, J = 613.5 Hz, 1H), 3.82–3.78 (m, 2H), 2.83–2.80 (m, 2H), 1.73–1.67 (m, 2H), 1.62–1.56 (m, 2H), 1.42–1.36 (m, 2H) and 1.31–1.29 (m, 4H) somewhat overlapped, 0.92 (t, J = 7.5 Hz) and 0.90 (t, J = 6.5 Hz) overlapped, total intensity 6H.

Butyl N-benzylphosphonamidate (**3d**): eluent: dichloromethane – methyl alcohol 97:3 → 9:1 → 1:1 mixtures used in volumes of 150 ml – 250 ml – 50 ml, respectively; Yield: 0.27 g (60%) as a colorless oil; ^{31}P {^{1}H} NMR (202 MHz, $CDCl_3$) δ 4.7; ^{13}C {^{1}H} NMR (126 MHz, $CDCl_3$) δ 134.1 (C_α), 128.73 (s, C_γ^*), 128.71 (s, C_β^*), 128.3 (s, C_δ), 63.6 (d, *J* = 4.5 Hz, OCH_2), 42.9 (s, CH_2Ph), 32.8 (d, *J* = 6.8 Hz, OCH_2CH_2), 19.0 (s, CH_2CH_3), 13.8 (s, CH_2CH_3), *may be reversed; ^{1}H NMR (500 MHz, $CDCl_3$) δ 7.47–7.40 and 7.38–7.31 (m, 5H), 6.55 (d, *J* = 621.4 Hz, 1H), 3.97 (s, 2H), 3.76–3.67 (m, 2H), 2.75 (bs, 1H), 1.57–1.49 (m, 2H), 1.37–1.28 (m, 2H), 0.89 (t, *J* = 7.4 Hz, 3H).

1.3. General Procedure for the Synthesis of Butyl *N,N'*-dibutylphosphonamid-imidate (5) and Butyl *N*-butyl-*N'*-propylphosphonamid-imidate (6)

A mixture of 2.0 mmol (0.39 mL) of dibutyl phosphite and 10.0 mmol of propylamine (0.82 mL) or butylamine (0.99 mL) was heated at 135°C in a vial in a CEM Discover Microwave reactor for 3h. The crude products obtained after removing the volatile components in vacuum were purified by flash column chromatography on silica gel applying dichloromethane – methyl alcohol 97:3 → 9:1 → 1:1 mixtures as specified below. Products **5** and **6** obtained were characterized below.

Butyl N,N'-dibutylphosphonamid-imidate (**5**): eluent: dichloromethane – methyl alcohol 97:3 → 9:1 → 1:1 mixtures used in volumes of 150 ml – 250 ml – 150 ml, respectively; Yield: 0.16 g (32 %) as a colorless oil; ^{31}P {^{1}H} NMR (122 MHz, $CDCl_3$) δ 3.6; ^{13}C {^{1}H} NMR (126 MHz, $CDCl_3$) δ 63.5 (d, *J* = 3.8 Hz, OCH_2), 47.3 (s, 2xNCH_2), 32.9 (d, *J* = 6.9 Hz, OCH_2CH_2), 27.8 (s, 2xNCH_2CH_2), 20.2 (s, 2xCH_2CH_3), 19.1 (s, CH_2CH_3), 13.7 (s, CH_2CH_3), 13.6 (s, 2xCH_2CH_3); ^{1}H NMR (500 MHz, $CDCl_3$) δ 6.78 (d, *J* = 612.5 Hz, 1H), 3.84–3.80 (m, 2H), 2.81–2.78 (m, 4H), 1.80–1.74 (m, 4H), 1.63–1.57 (m, 2H), 1.43–1.34 (m, 6H), 0.94 (t, *J* = 7.4 Hz, 6H) and 0.92 (t, *J* = 7.3 Hz, 3H) overlapped.

Butyl N-propyl-N'-butylphosphonamid-imidate (**6**): eluent: dichloromethane – methyl alcohol 97:3 → 9:1 → 1:1 mixtures used in volumes of 150 ml – 250 ml – 150 ml, respectively; Yield: 0.18 g (39%) as a colorless oil; ^{31}P {^{1}H} NMR (202 MHz, $CDCl_3$) δ 3.5; ^{13}C {^{1}H} NMR (126 MHz, $CDCl_3$) δ 63.5 (d, *J* = 4.6 Hz, OCH_2), 49.0 (s, NCH_2), 47.2 (s, NCH_2), 32.9 (d, *J* = 7.0 Hz, $OCH_2\mathit{C}H_2$), 27.7 (s, $NCH_2\mathit{C}H_2$), 20.1 (s, $\mathit{C}H_2CH_2CH_3$), 19.2 (s, $\mathit{C}H_2CH_3$), 19.0 (s, $\mathit{C}H_2CH_3$), 13.7 (s, $CH_2\mathit{C}H_3$), 13.6 (s, $CH_2\mathit{C}H_3$), 11.3 (s, $CH_2\mathit{C}H_3$); ^{1}H NMR (500 MHz, $CDCl_3$) δ 6.78 (d, *J* = 611.9 Hz, 1H), 3.85–3.78 (m, 2H), 2.83–2.73 (m, 4H), 2.00 (bs, 1H), 1.87–1.73 (m, 4H), 1.63–1.56 (m, 2H), 1.43–1.33 (m, 4H), 0.97 (t, *J* = 7.4 Hz, 3H), 0.94 (t, *J* = 7.4 Hz, 3H) and 0.92 (t, *J* = 7.3 Hz, 3H) overlapped.

1.4. Synthesis of butyl *N*-butyl-*N*-propylphosphonamidate (7)

A mixture of 2.0 mmol (0.39 mL) of dibutyl phosphite and 6.0 mmol of propylamine (0.49 mL) was heated at 110°C in a vial in a CEM Discover Microwave reactor for 6h. The crude product obtained after removing the volatile components in vacuum was purified by flash column chromatography on silica gel applying dichloromethane – methyl alcohol 97:3 → 9:1 → 1:1 mixtures in volumes of 150 ml – 250 ml – 50 ml, respectively. Product **7** was obtained as a colorless oil. Yield: 0.11 g (23 %);^{31}P {^{1}H} NMR (202 MHz, $CDCl_3$) δ 3.7; ^{13}C {^{1}H} NMR (126 MHz, $CDCl_3$) δ 63.5 (d, *J* = 4.3 Hz, OCH_2), 49.0 (s, NCH_2), 47.2 (s, NCH_2), 32.9 (d, *J* = 6.9 Hz, $OCH_2\mathit{C}H_2$), 27.7 (s, $OCH_2\mathit{C}H_2$), 20.1 (s, $\mathit{C}H_2CH_3$), 19.3 (s, $\mathit{C}H_2CH_3$), 19.0 (s, $\mathit{C}H_2CH_3$), 13.7 (s, $CH_2\mathit{C}H_3$), 13.6 (s, $CH_2\mathit{C}H_3$), 11.3 (s, $CH_2\mathit{C}H_3$); ^{1}H NMR (500 MHz, $CDCl_3$) δ 6.77 (d, *J* = 622.2 Hz, 1H), 3.86–3.82 (m, 2H), 2.80 (bs, 4H), 1.87–1.74 (m, 4H), 1.64–1.58 (m, 2H), 1.43–1.34 (m, 4H), 0.97 (t, *J* = 7.4 Hz, 3H), 0.94 (t, *J* = 7.4 Hz, 3H) and 0.92 (t, *J* = 7.4 Hz, 3H) overlapped.

2. ^{31}P, ^{13}C, ^{1}H NMR spectra of the compounds 1a-d, 2a-d, 3a-d, 5, 6 and 7 synthesized

^{31}P {^{1}H} NMR (202 MHz, $CDCl_3$) spectra for 1a

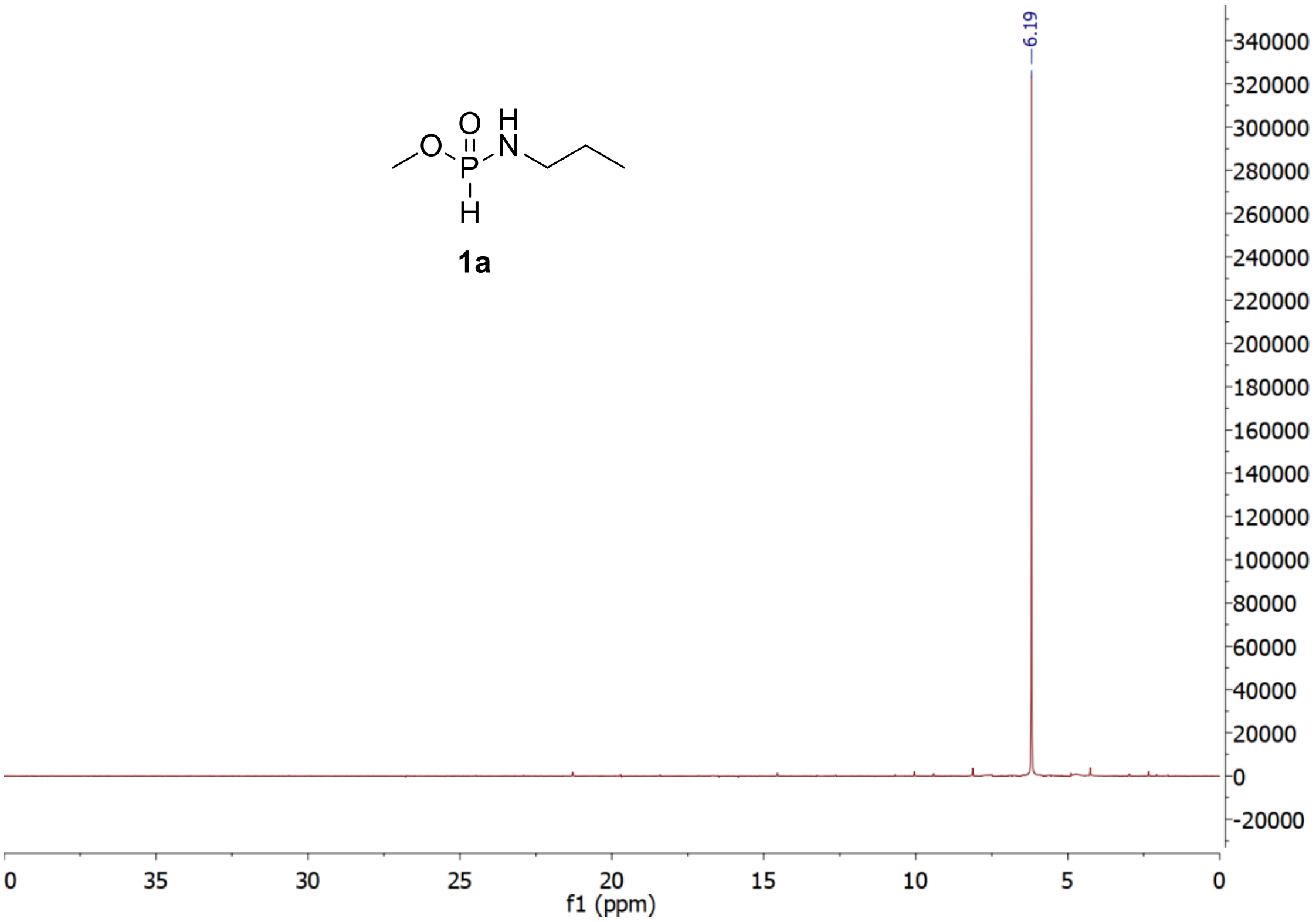

^{13}C {^{1}H} NMR (126 MHz, $CDCl_3$) spectra for 1a

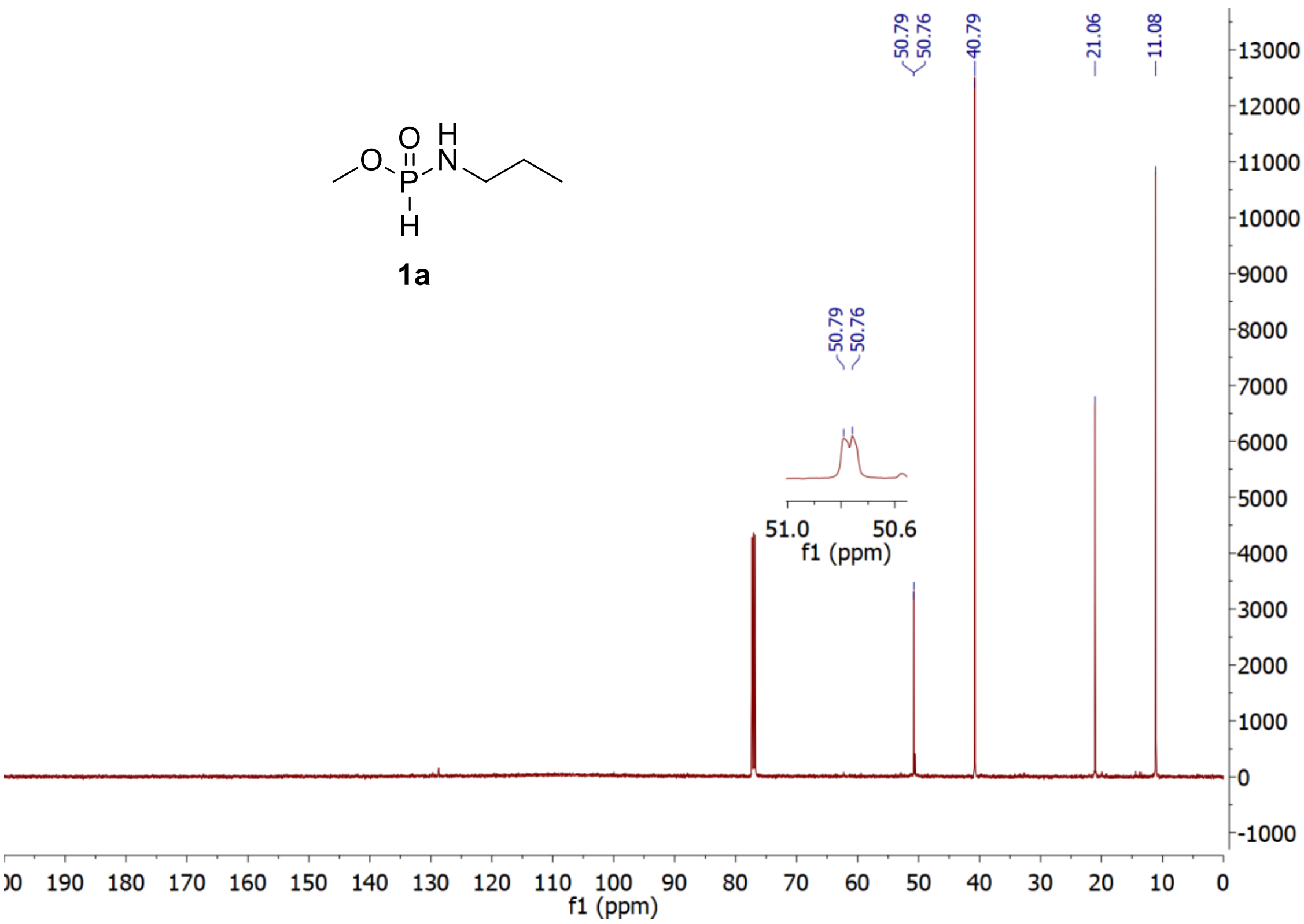

^{1}H NMR (500 MHz, $CDCl_3$) spectra for 1a

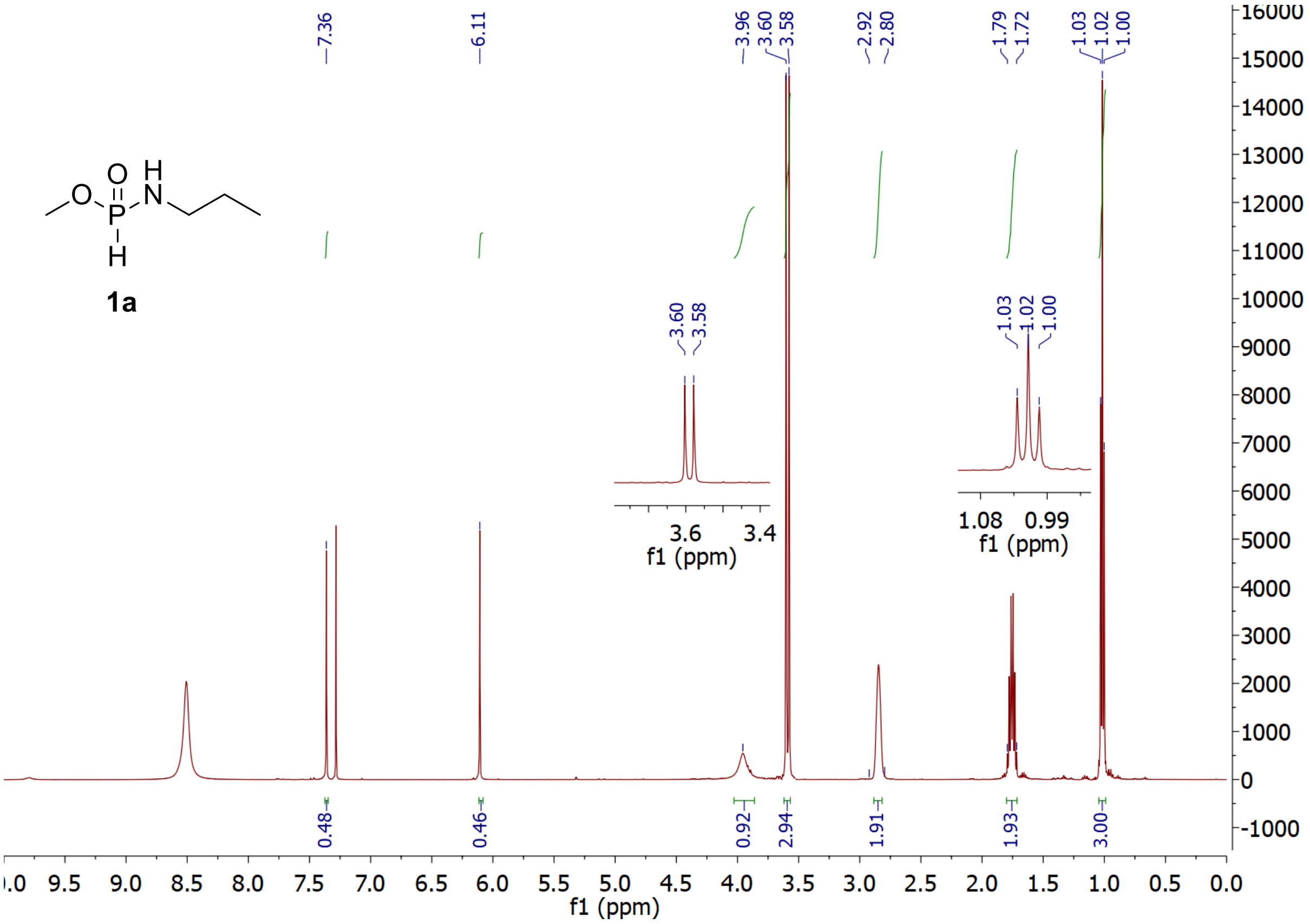

^{31}P {^{1}H} NMR (122 MHz, $CDCl_3$) spectra for 1b

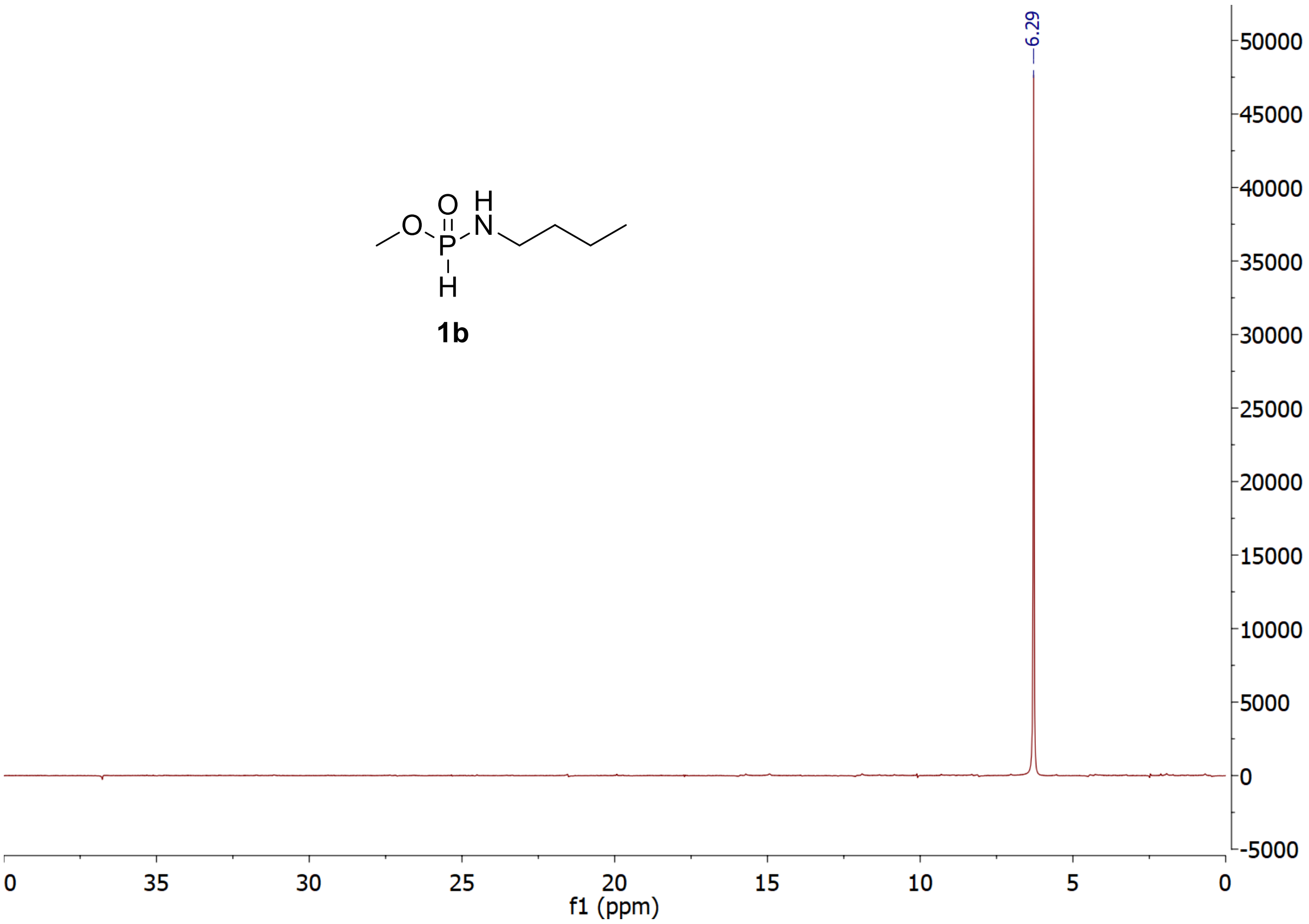

^{13}C {^{1}H} NMR (75 MHz, $CDCl_3$) spectra for 1b

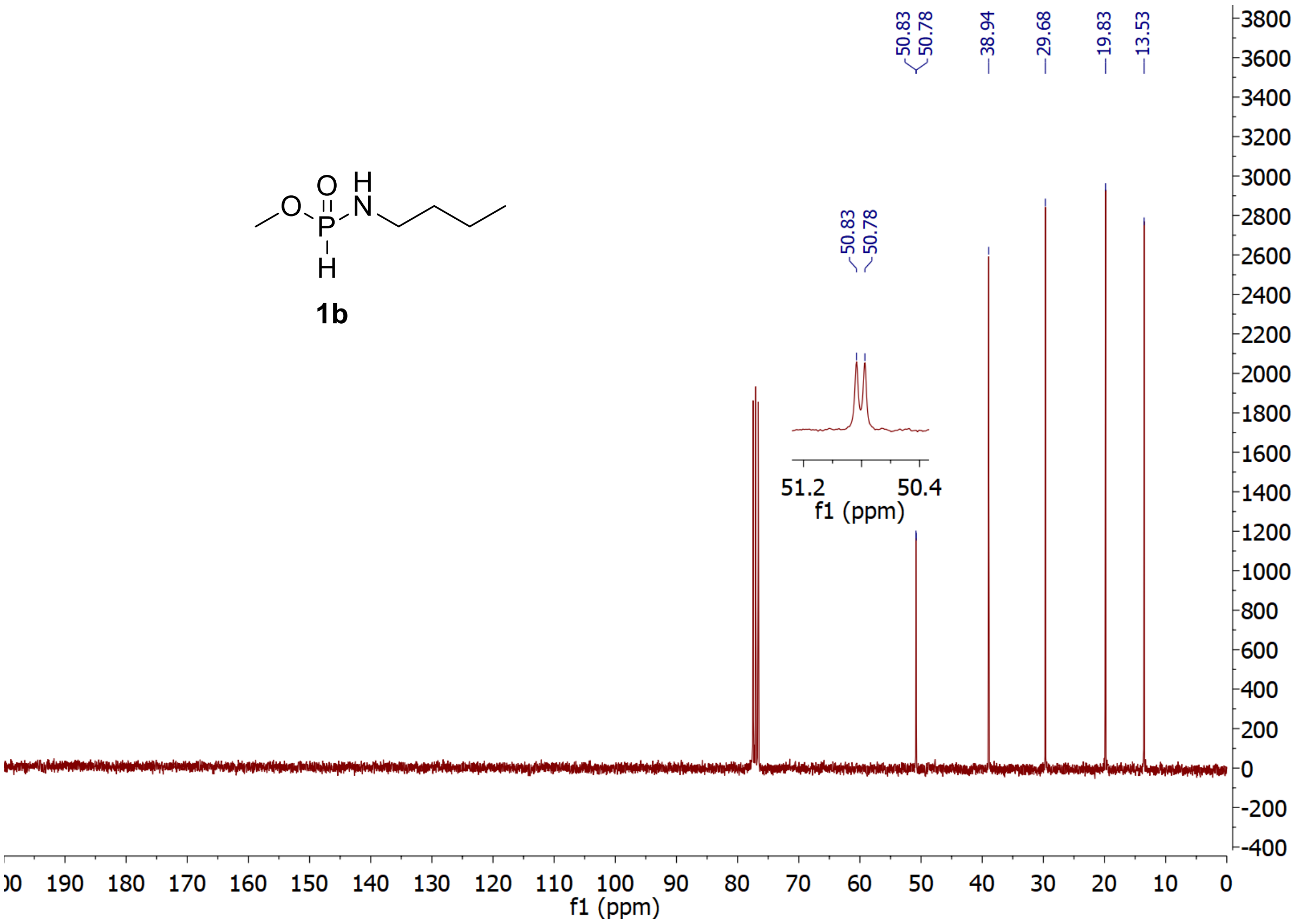

^{1}H NMR (300 MHz, $CDCl_3$) spectra for 1b

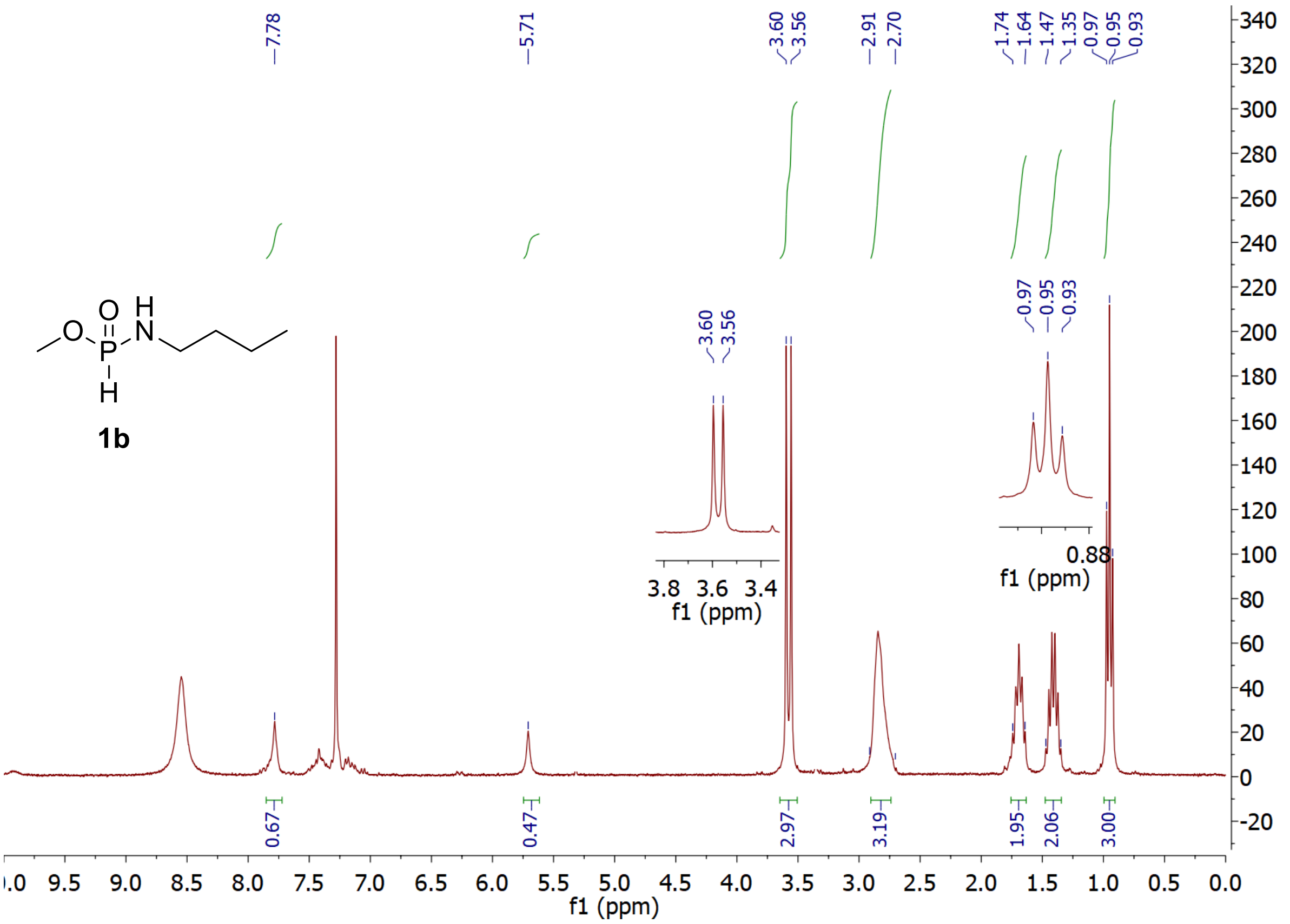

^{31}P {^{1}H} NMR (122 MHz, $CDCl_3$) spectra for 1c

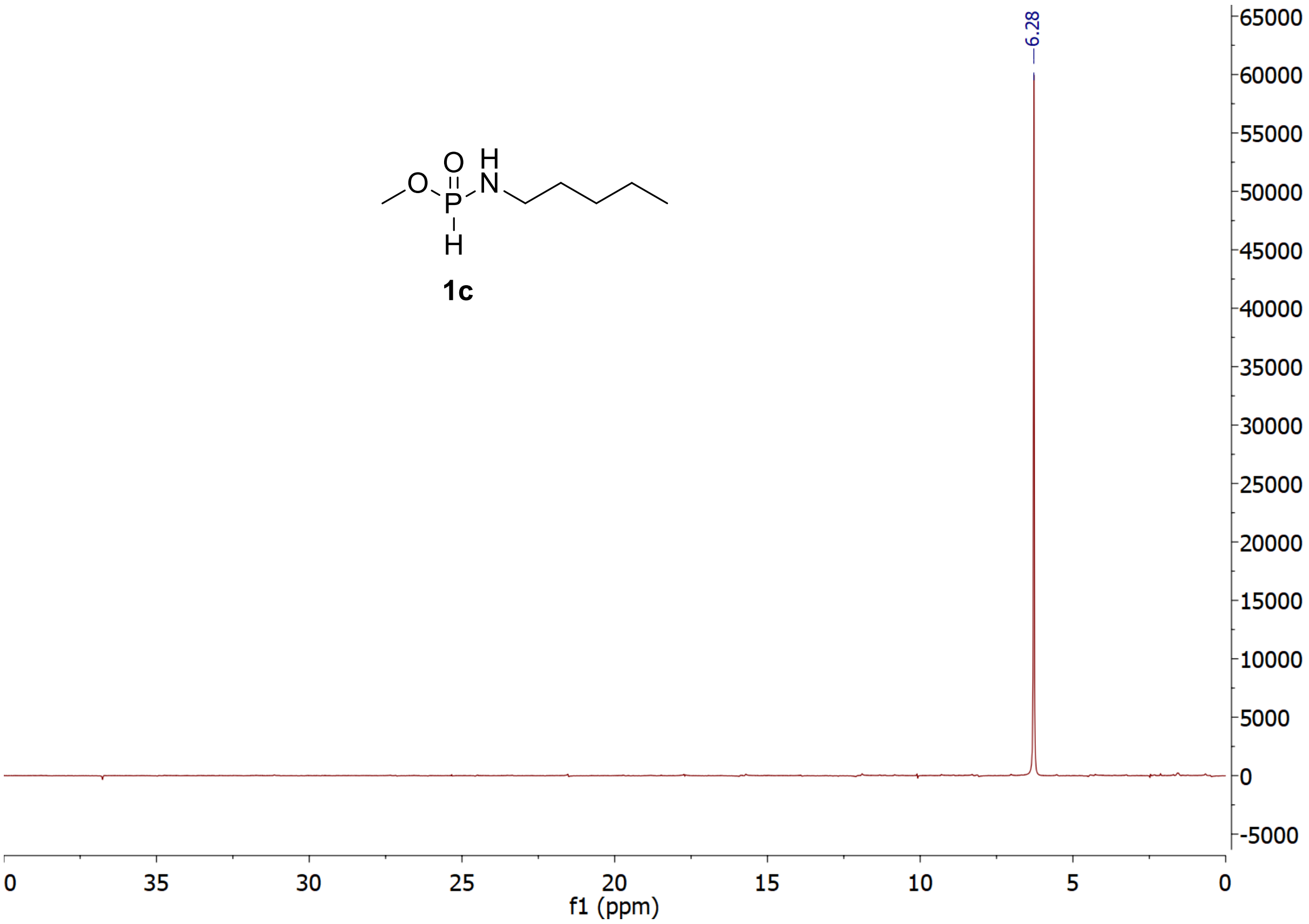

^{13}C {^{1}H} NMR (75 MHz, $CDCl_3$) spectra for 1c

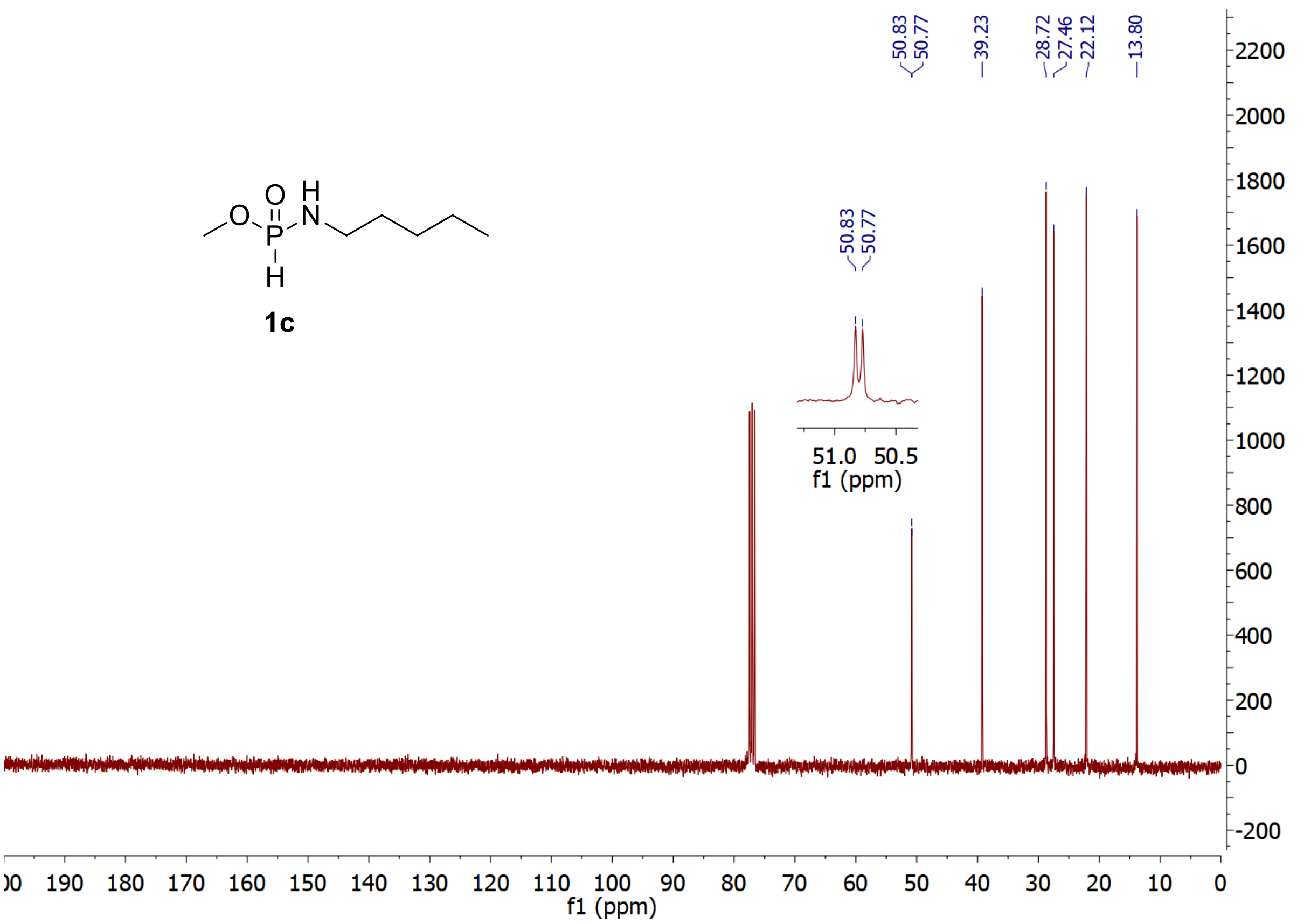

^{1}H NMR (300 MHz, $CDCl_3$) spectra for 1c

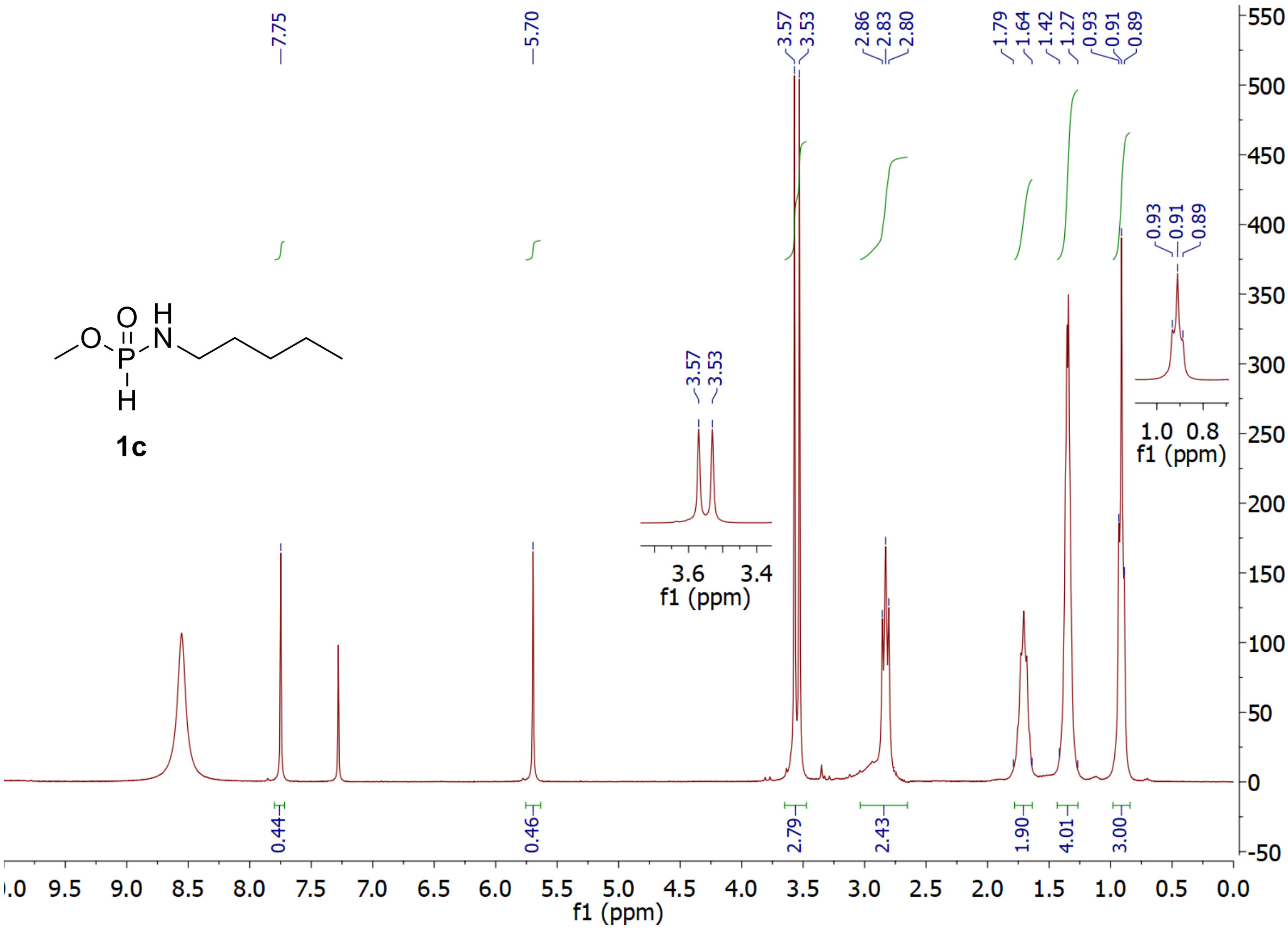

^{31}P {^{1}H} NMR (122 MHz, $CDCl_3$) spectra for 1d

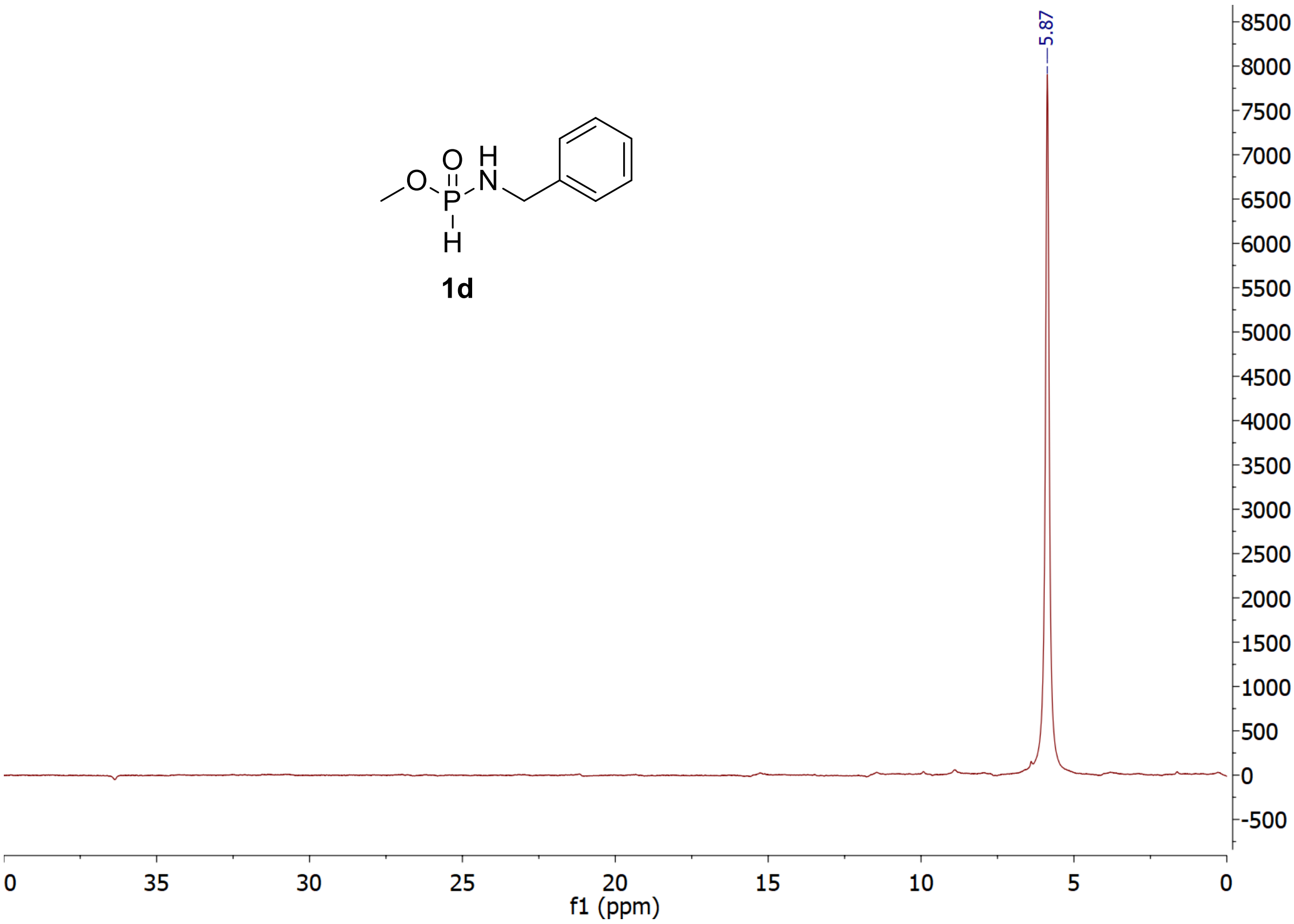

^{13}C {^{1}H} NMR (75 MHz, $CDCl_3$) spectra for 1d

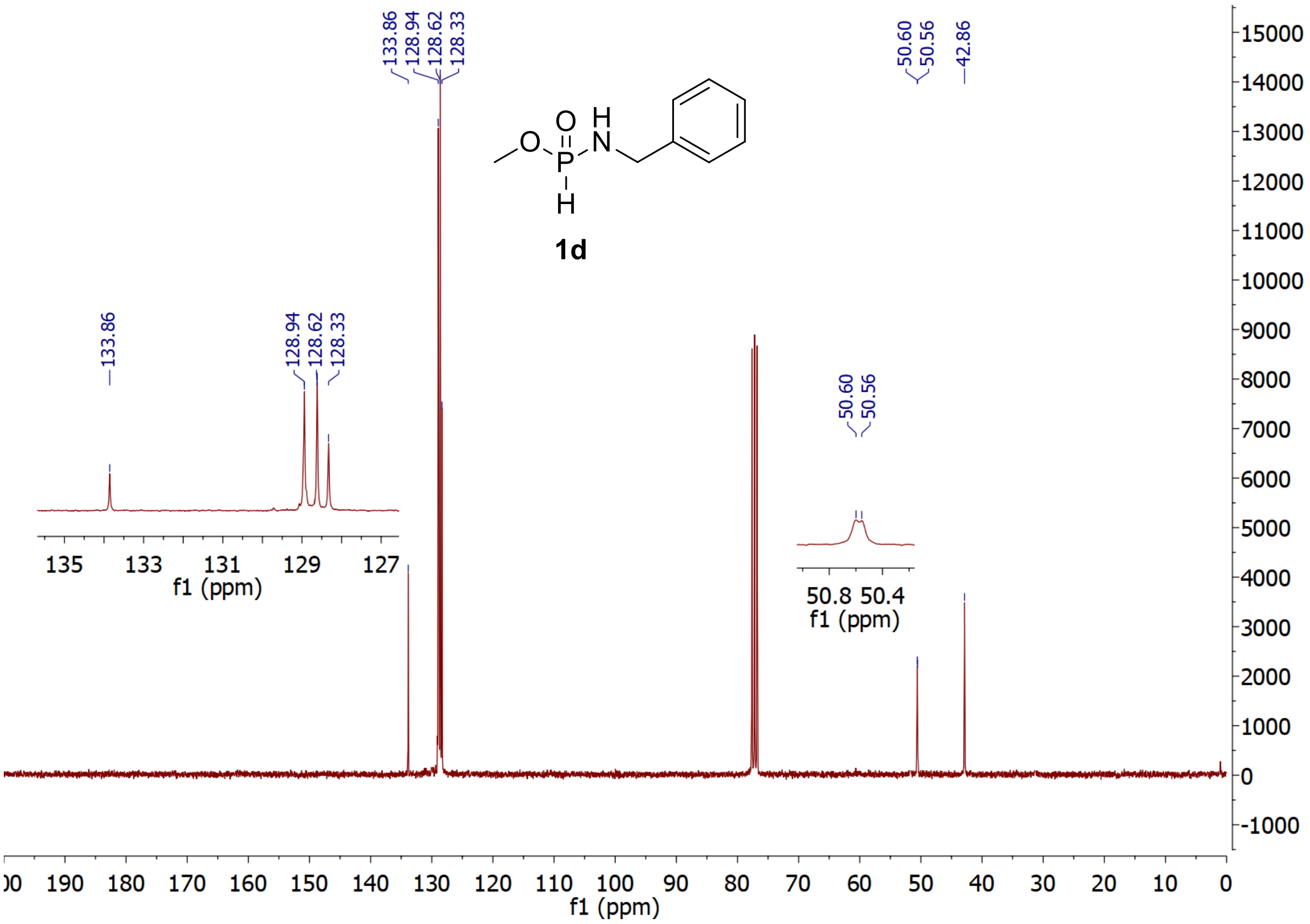

^{1}H NMR (500MHz, $CDCl_3$) spectra for 1d

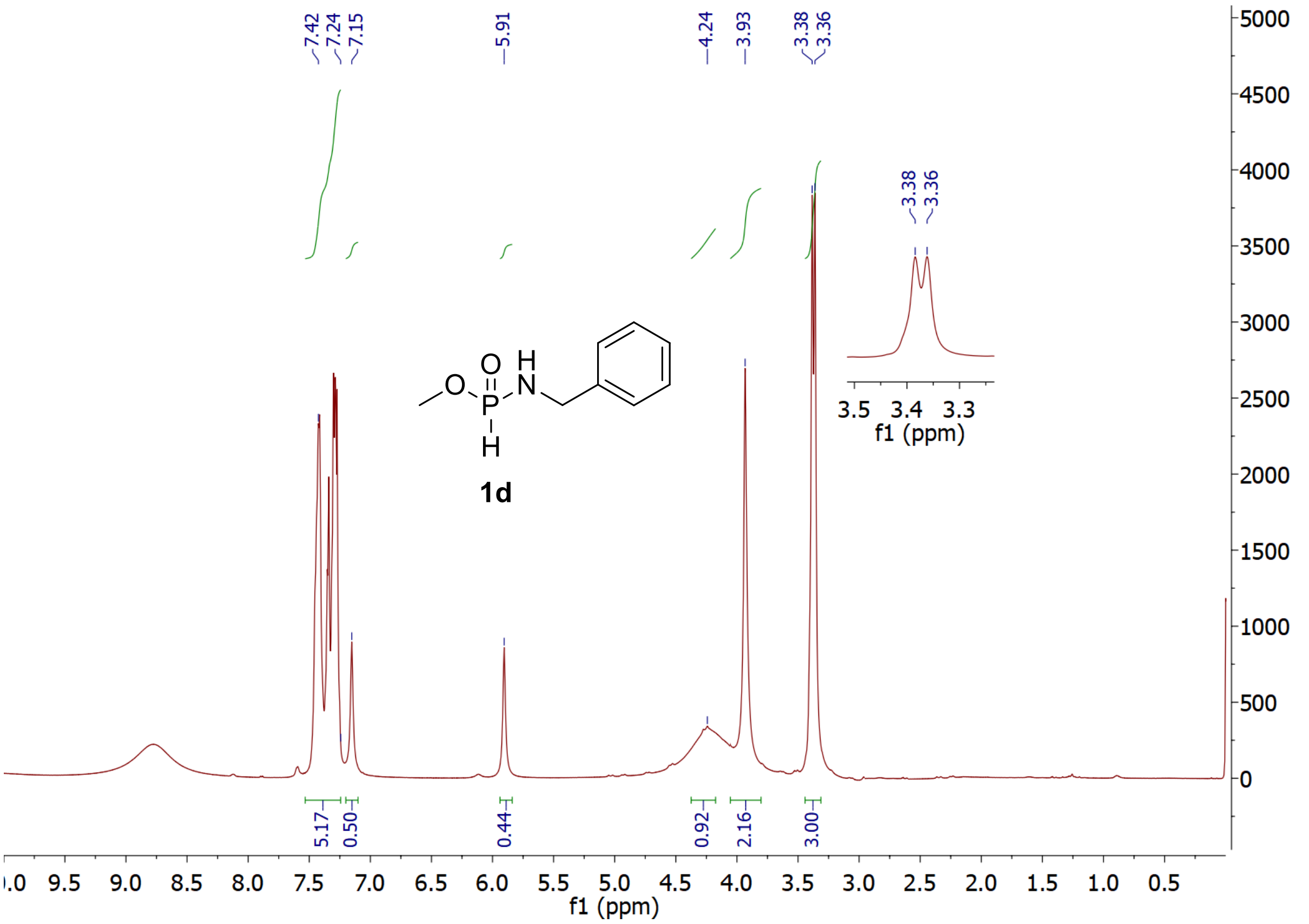

$^{31}P\ \{^{1}H\}$ NMR (202MHz, $CDCl_3$) spectra for 2a

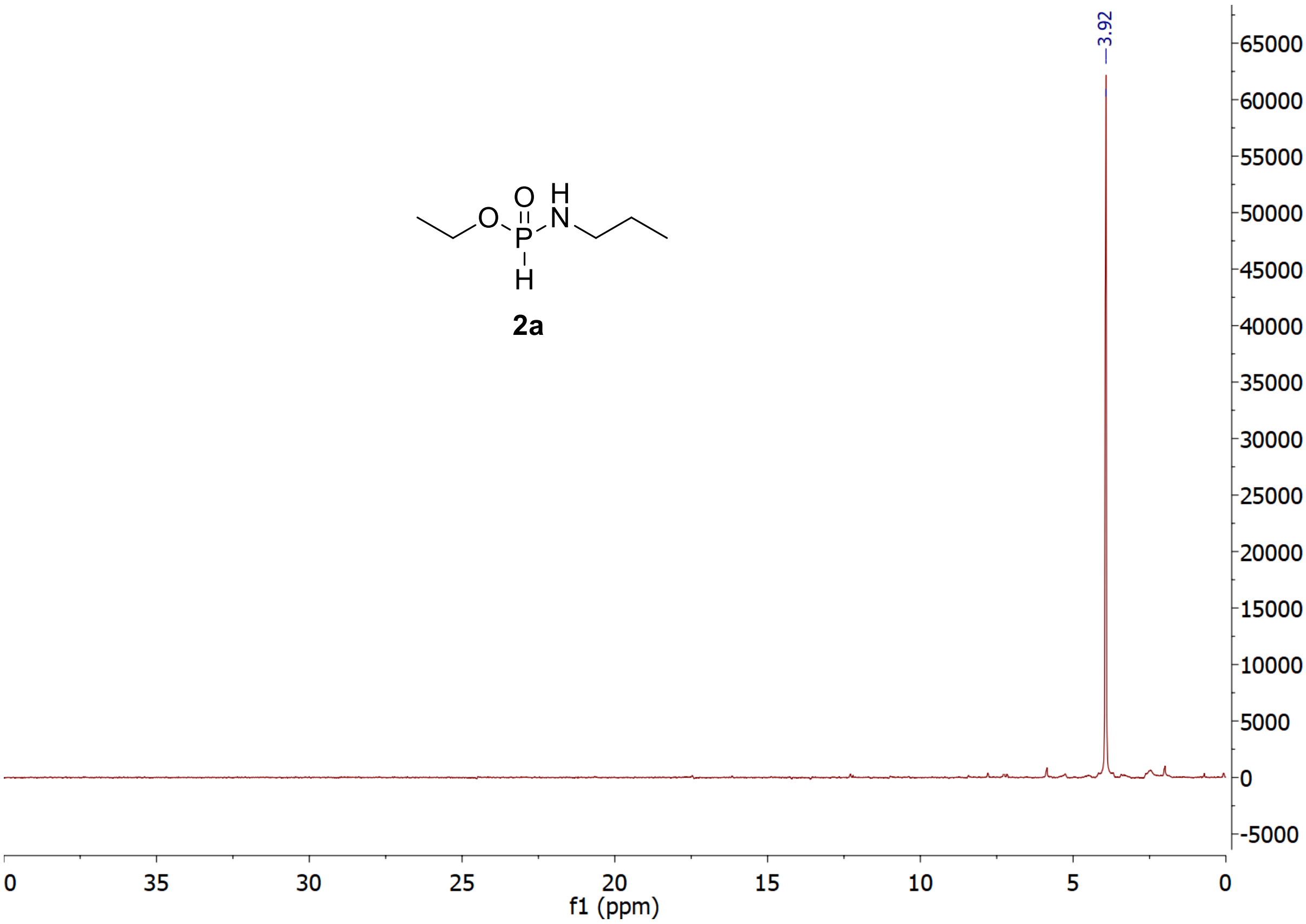

$^{13}C\ \{^{1}H\}$ NMR (75 MHz, $CDCl_3$) spectra for 2a

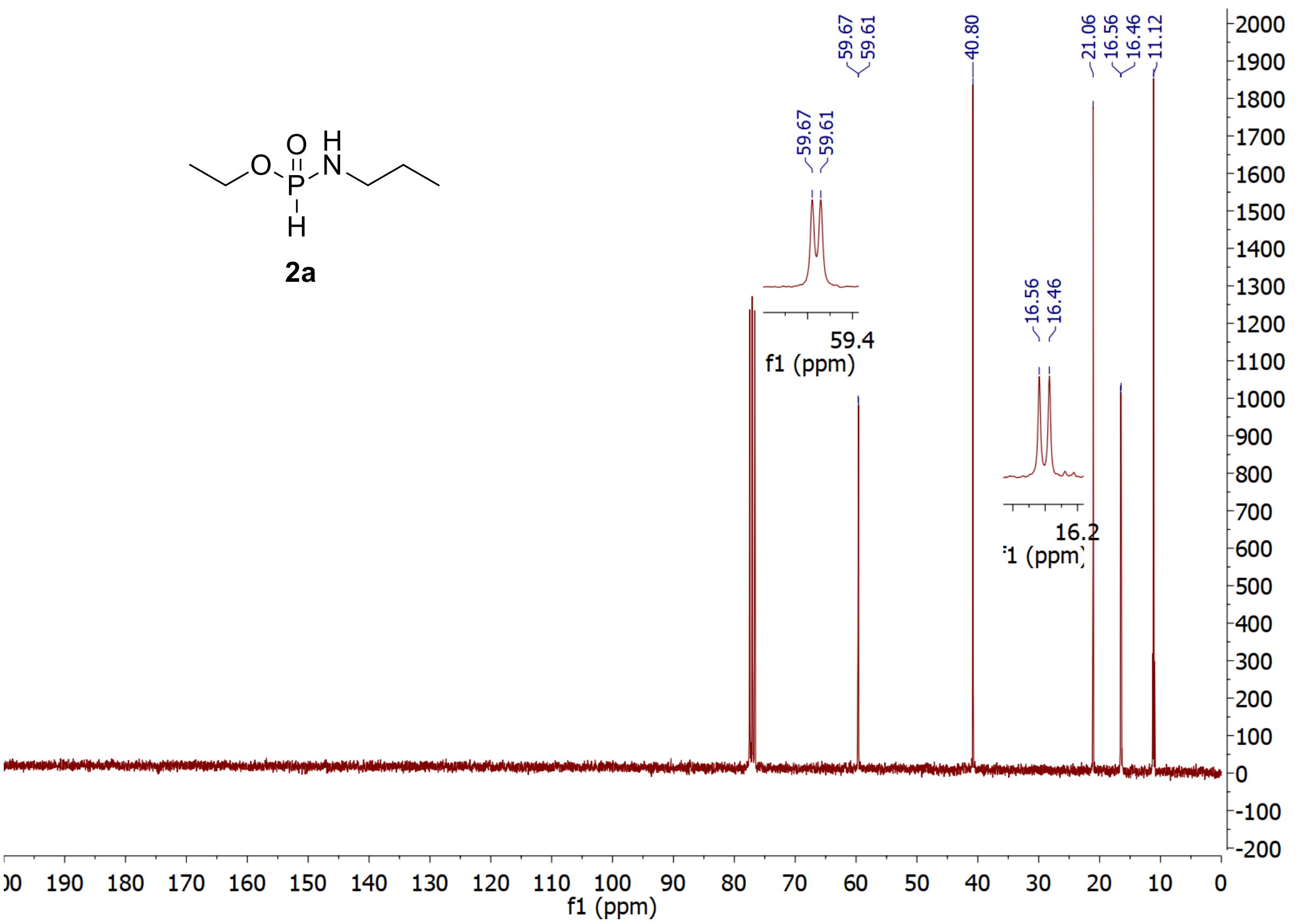

^{1}H NMR (300 MHz, $CDCl_3$) spectra for 2a

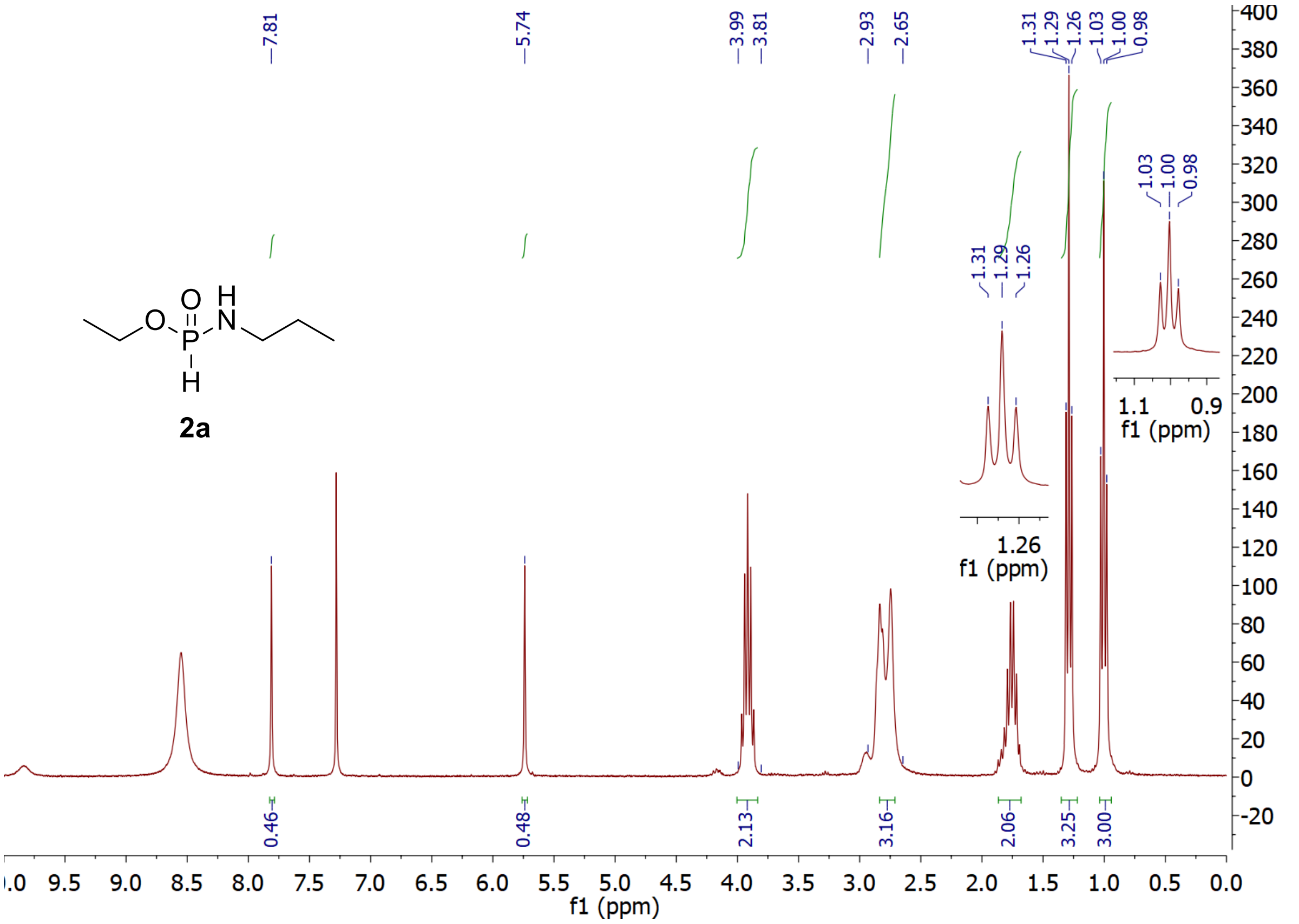

^{31}P {^{1}H} NMR (122 MHz, $CDCl_3$) spectra for 2b

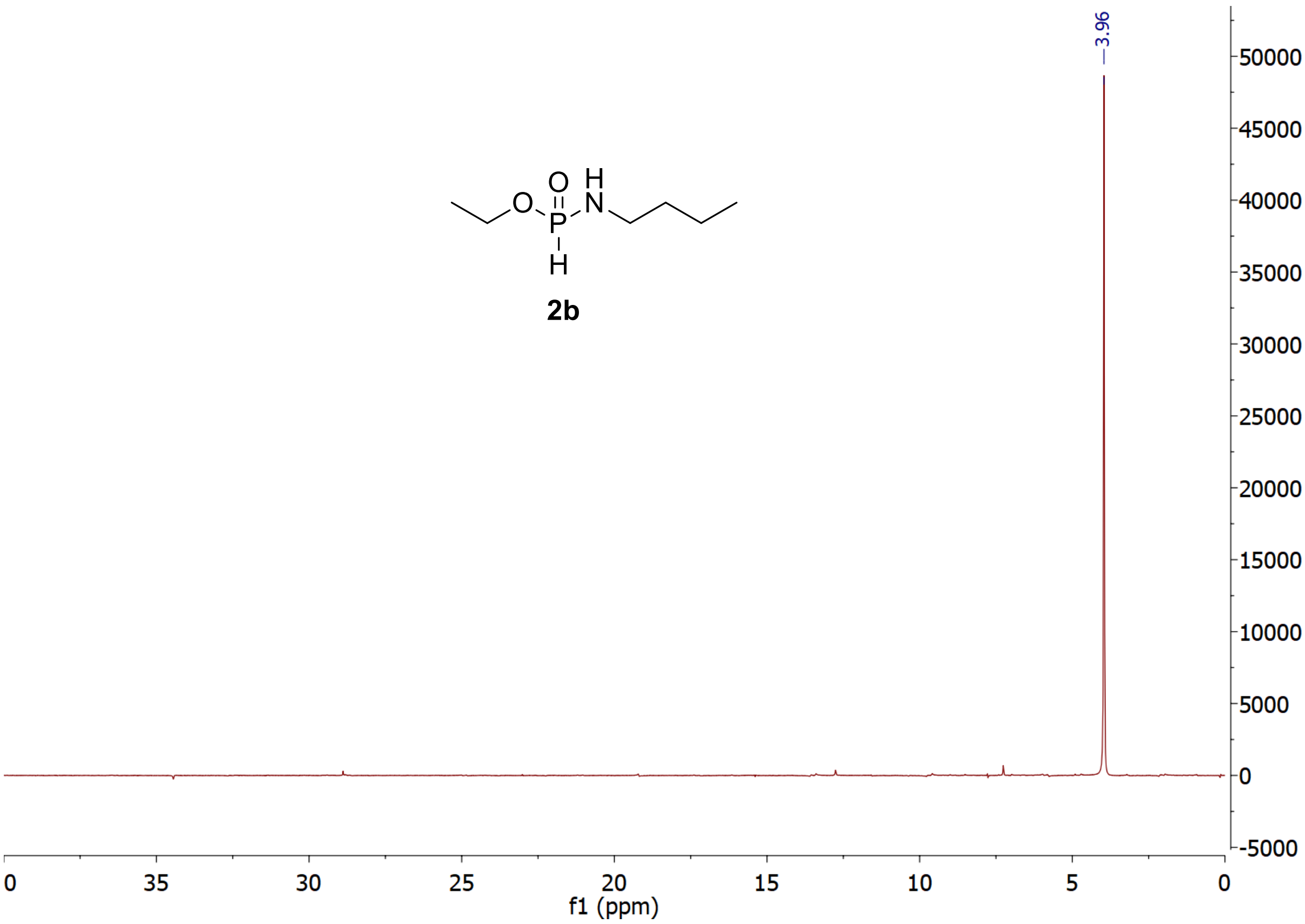

$^{13}C\{^1H\}$ NMR (75 MHz, CDCl$_3$) spectra for 2b

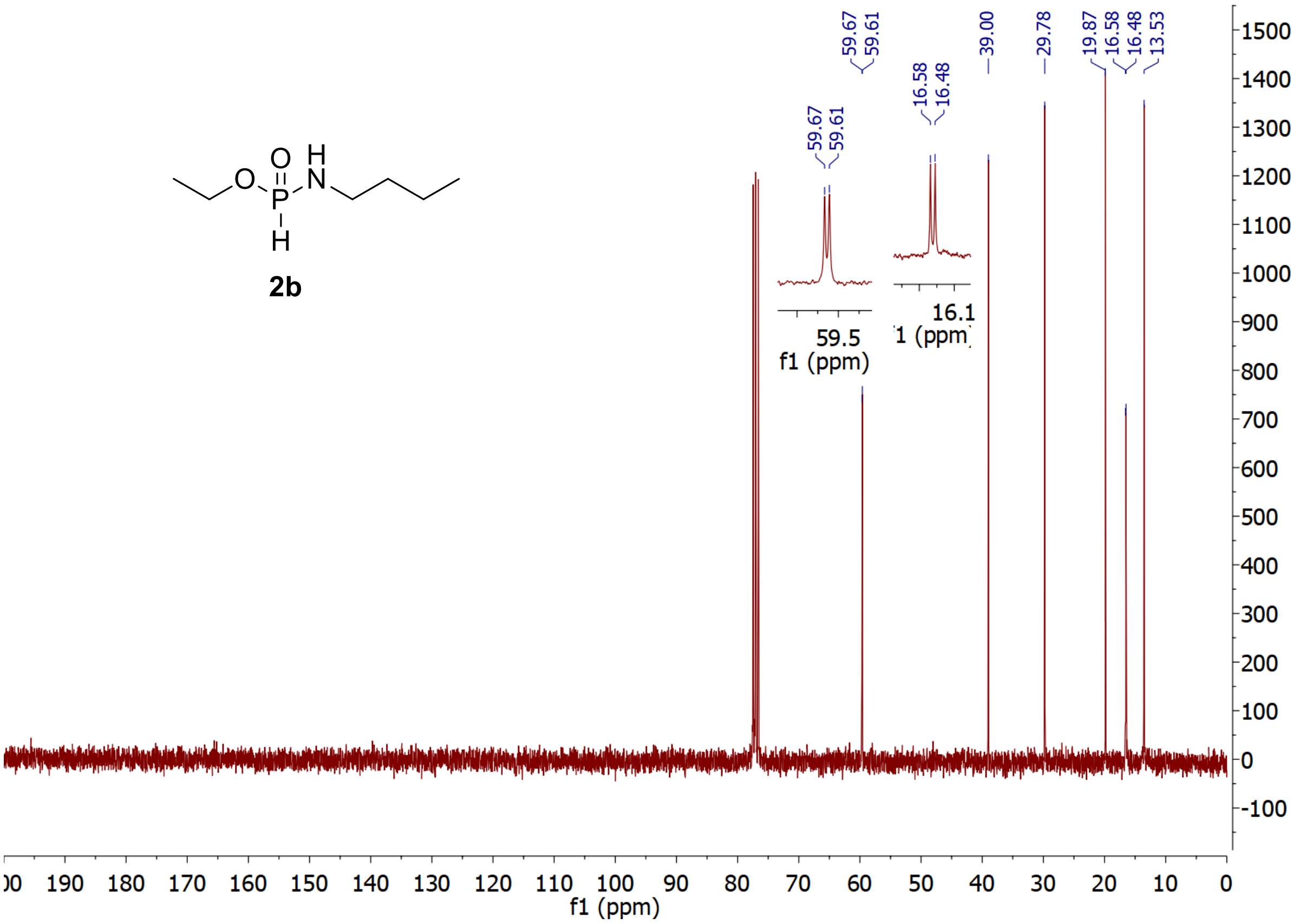

^1H NMR (300 MHz, CDCl$_3$) spectra for 2b

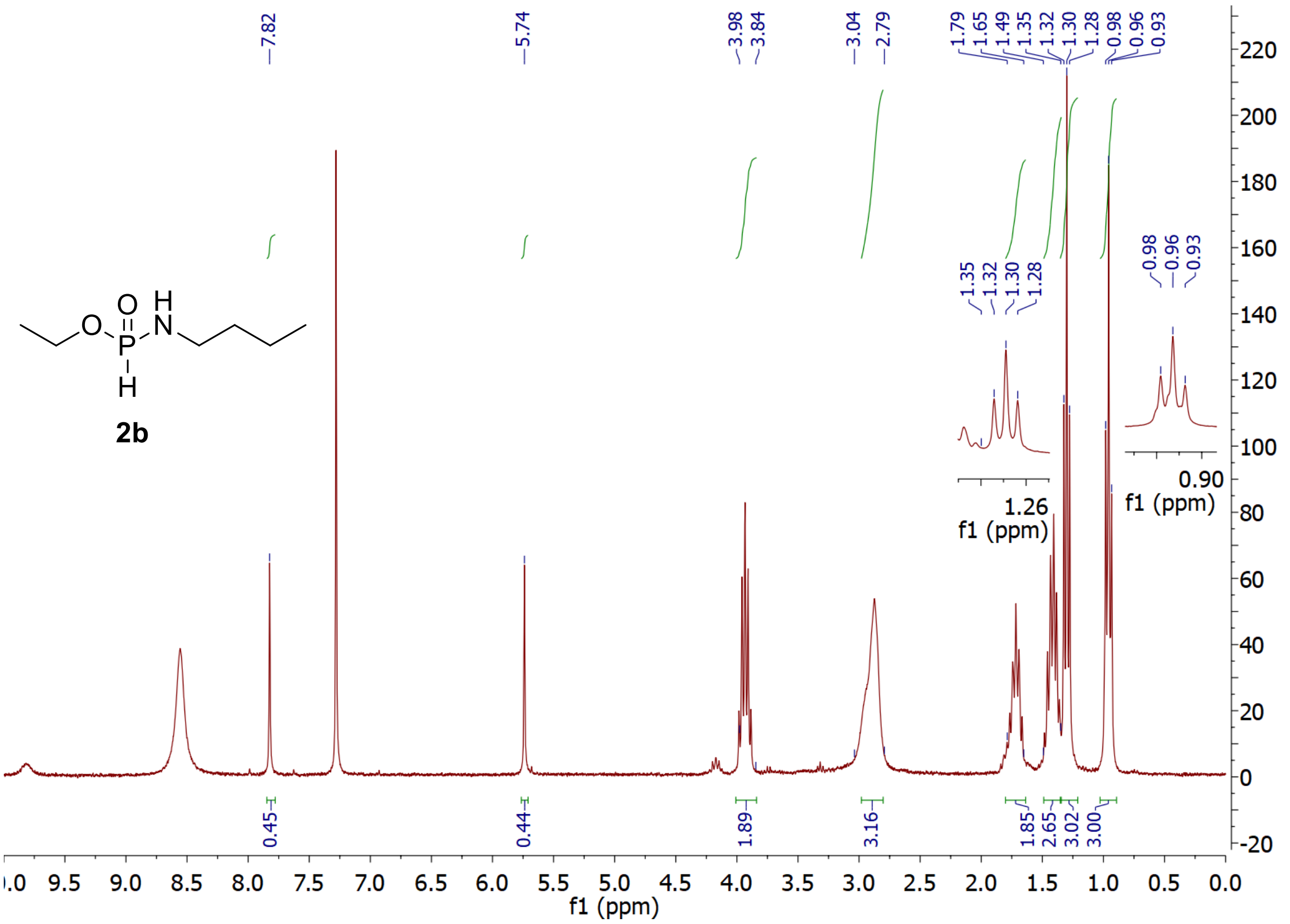

^{31}P {^{1}H} NMR (122 MHz, CDCl₃) spectra for 2c

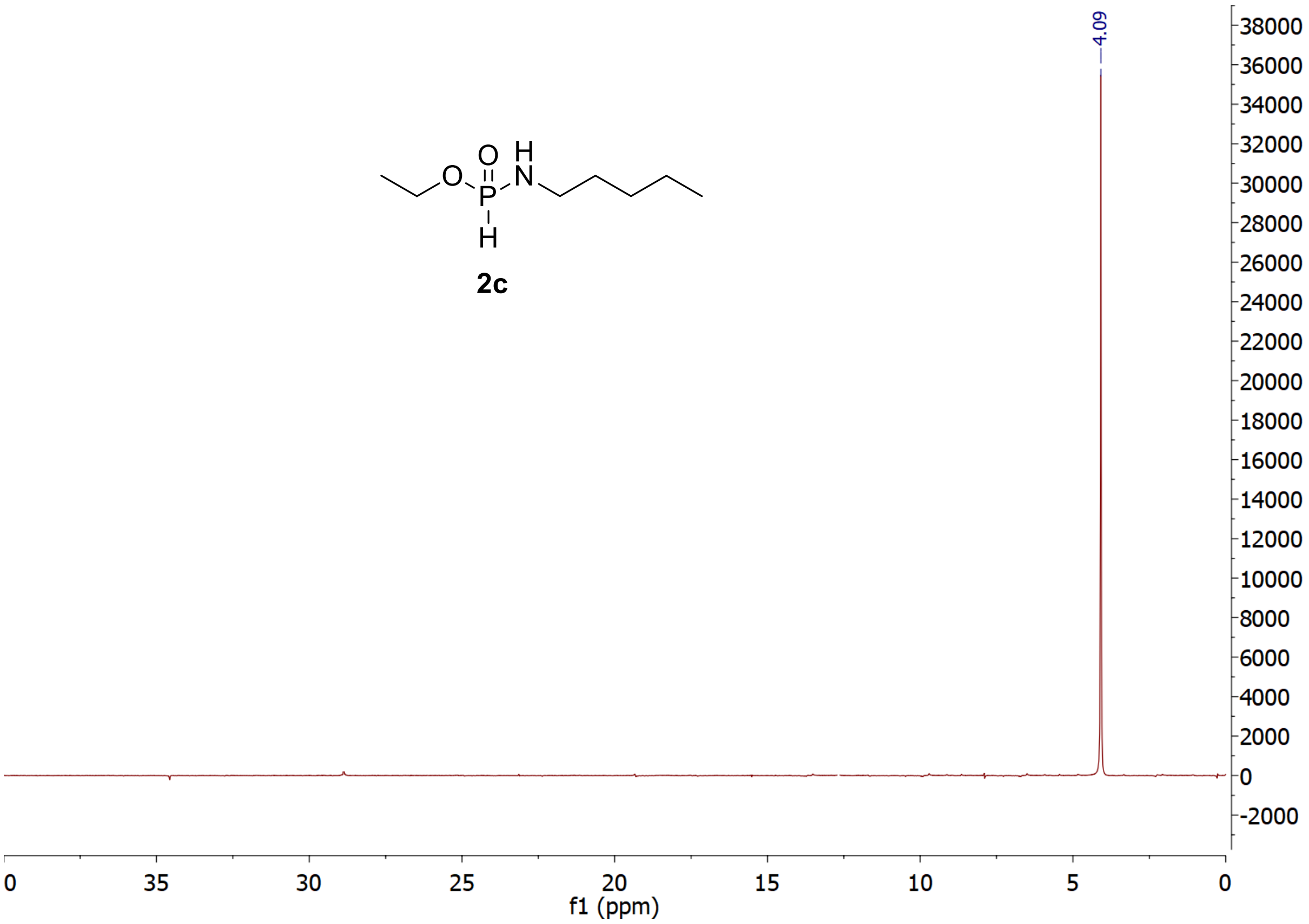

^{13}C {^{1}H} NMR (75 MHz, CDCl₃) spectra for 2c

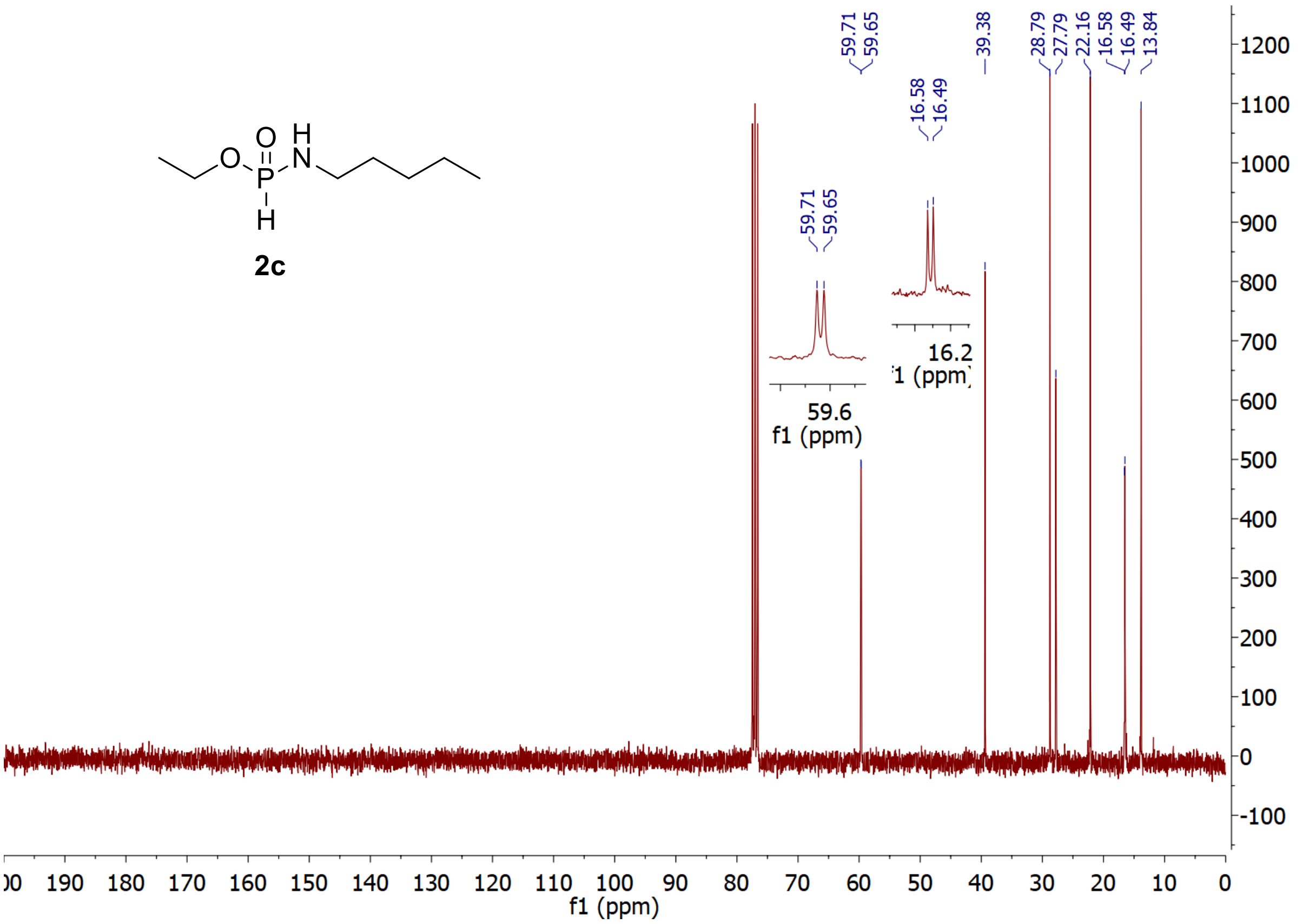

^{1}H NMR (300 MHz, CDCl3) spectra for 2c

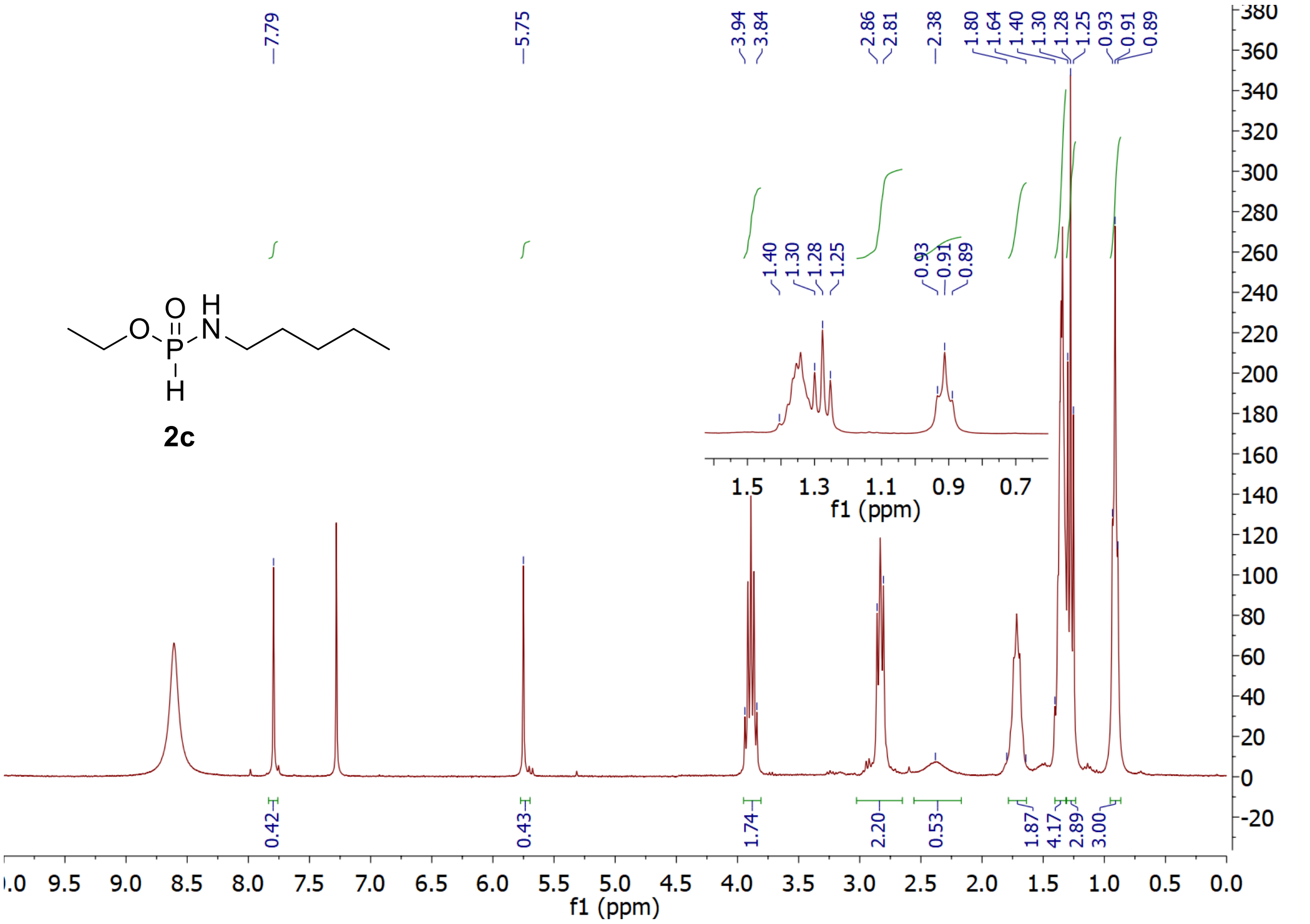

^{31}P {^{1}H} NMR (202 MHz, CDCl3) spectra for 2d

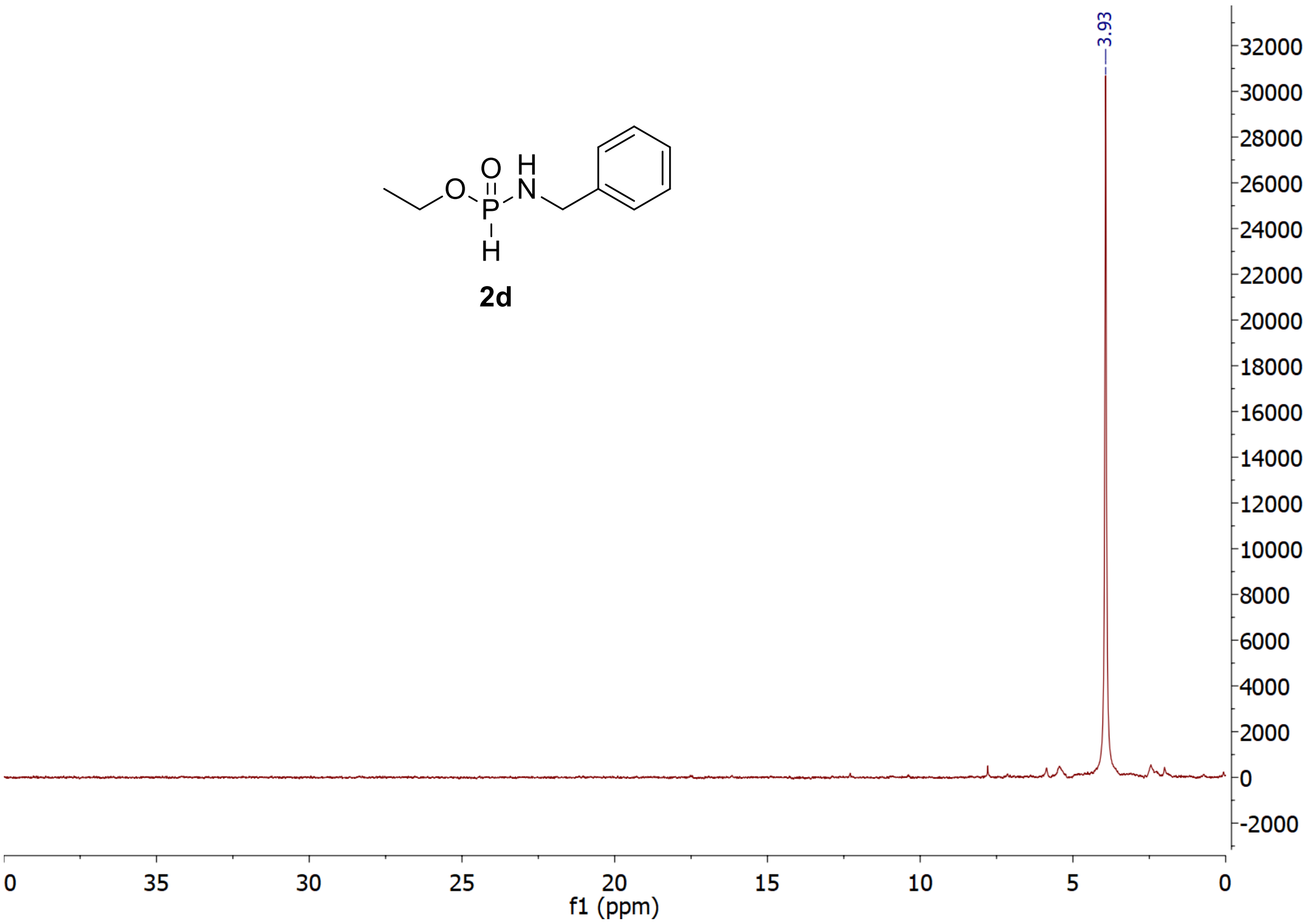

$^{13}C\{^{1}H\}$ NMR (75 MHz, $CDCl_3$) spectra for 2d

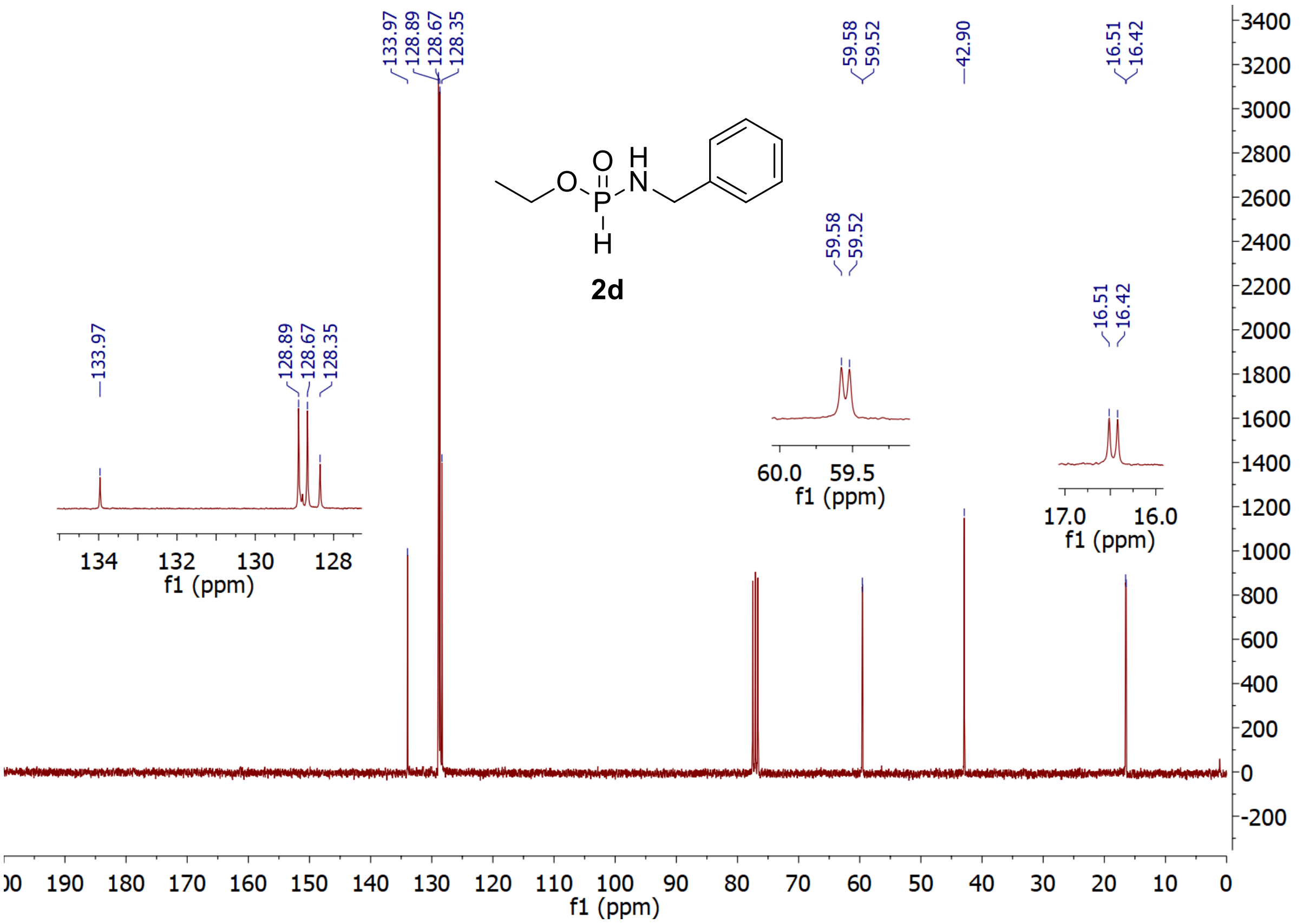

^{1}H NMR (500 MHz, $CDCl_3$) spectra for 2d

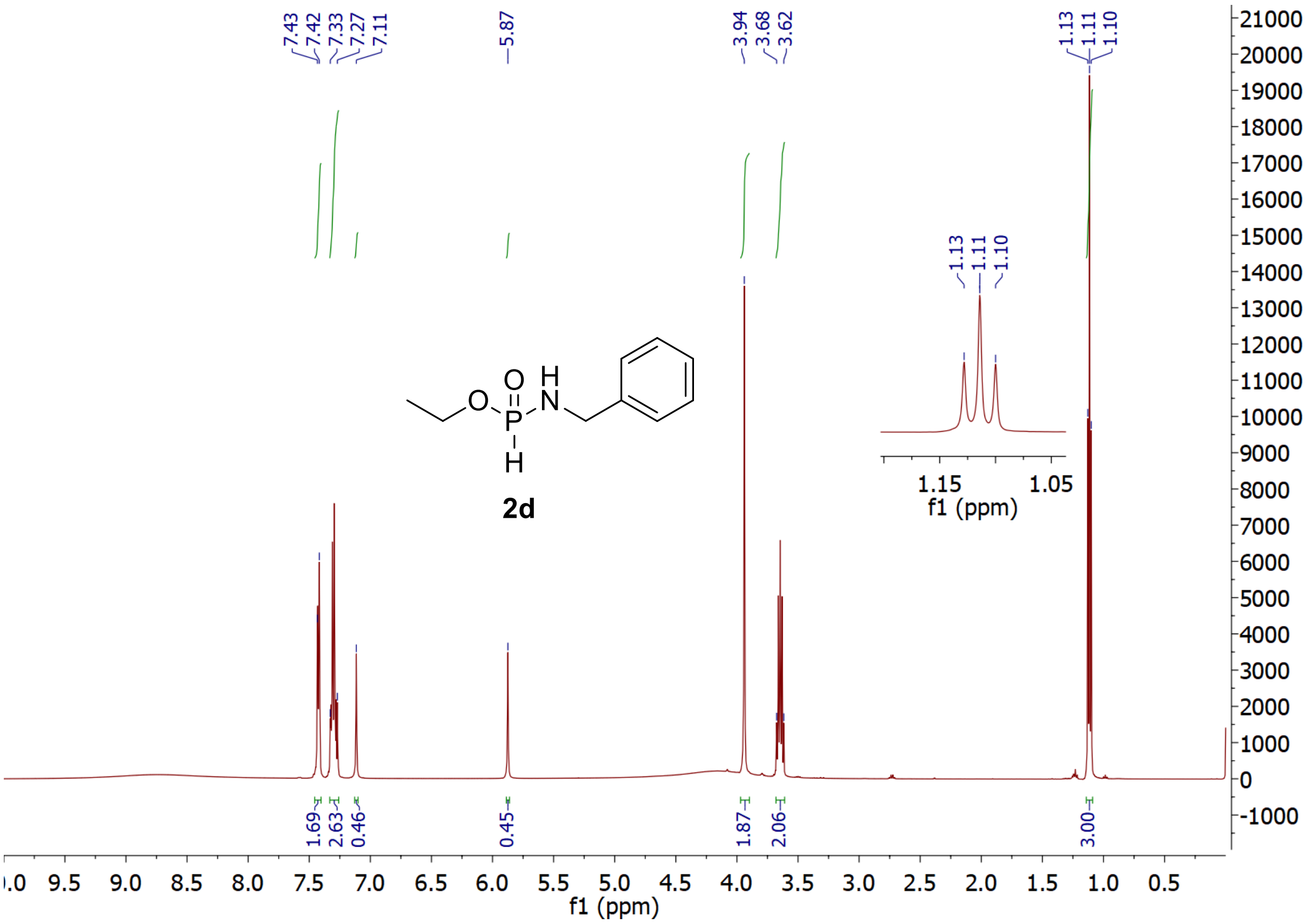

^{31}P {^{1}H} NMR (122 MHz, CDCl3) spectra for 3a

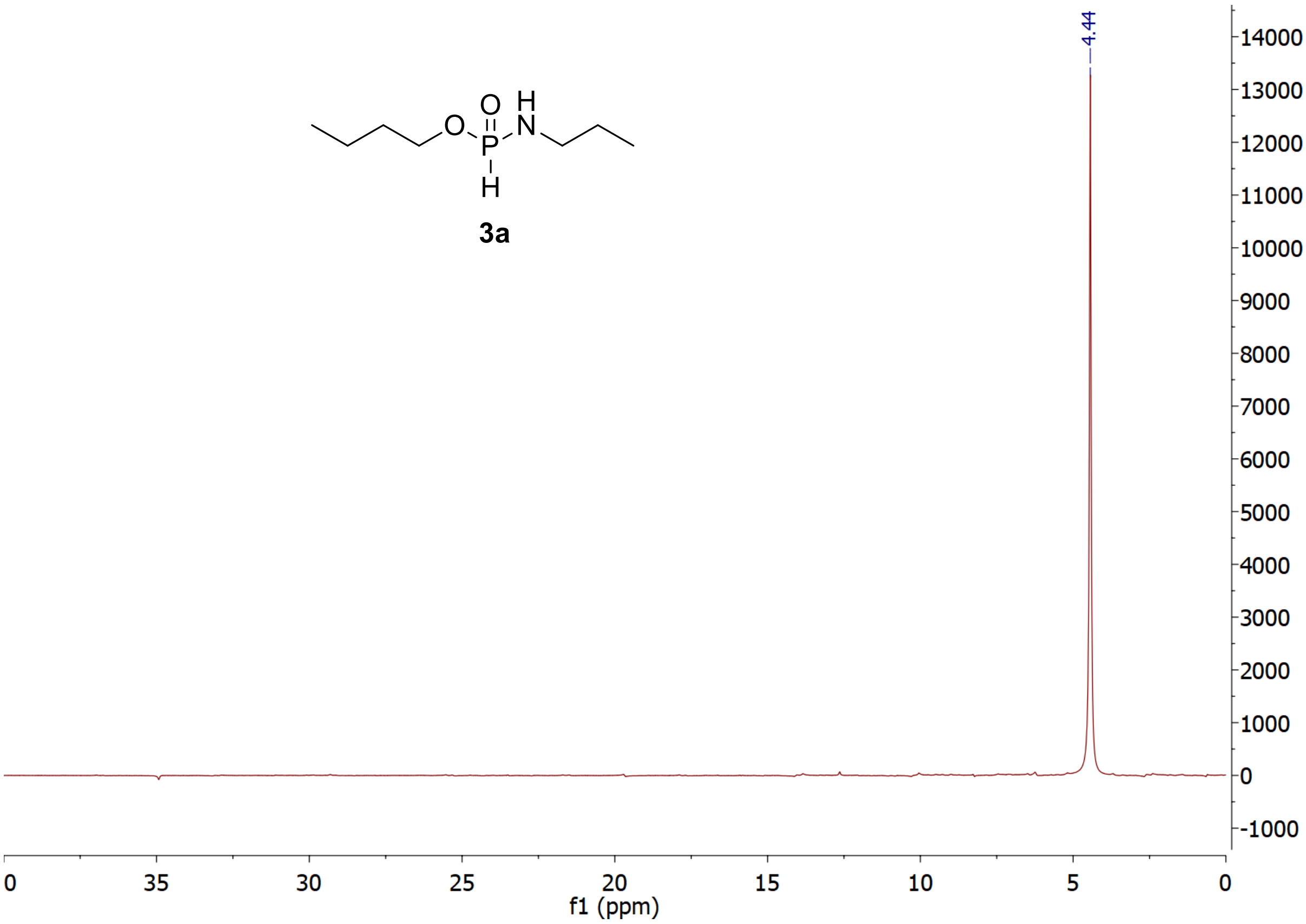

^{13}C {^{1}H} NMR (75 MHz, CDCl3) spectra for 3a

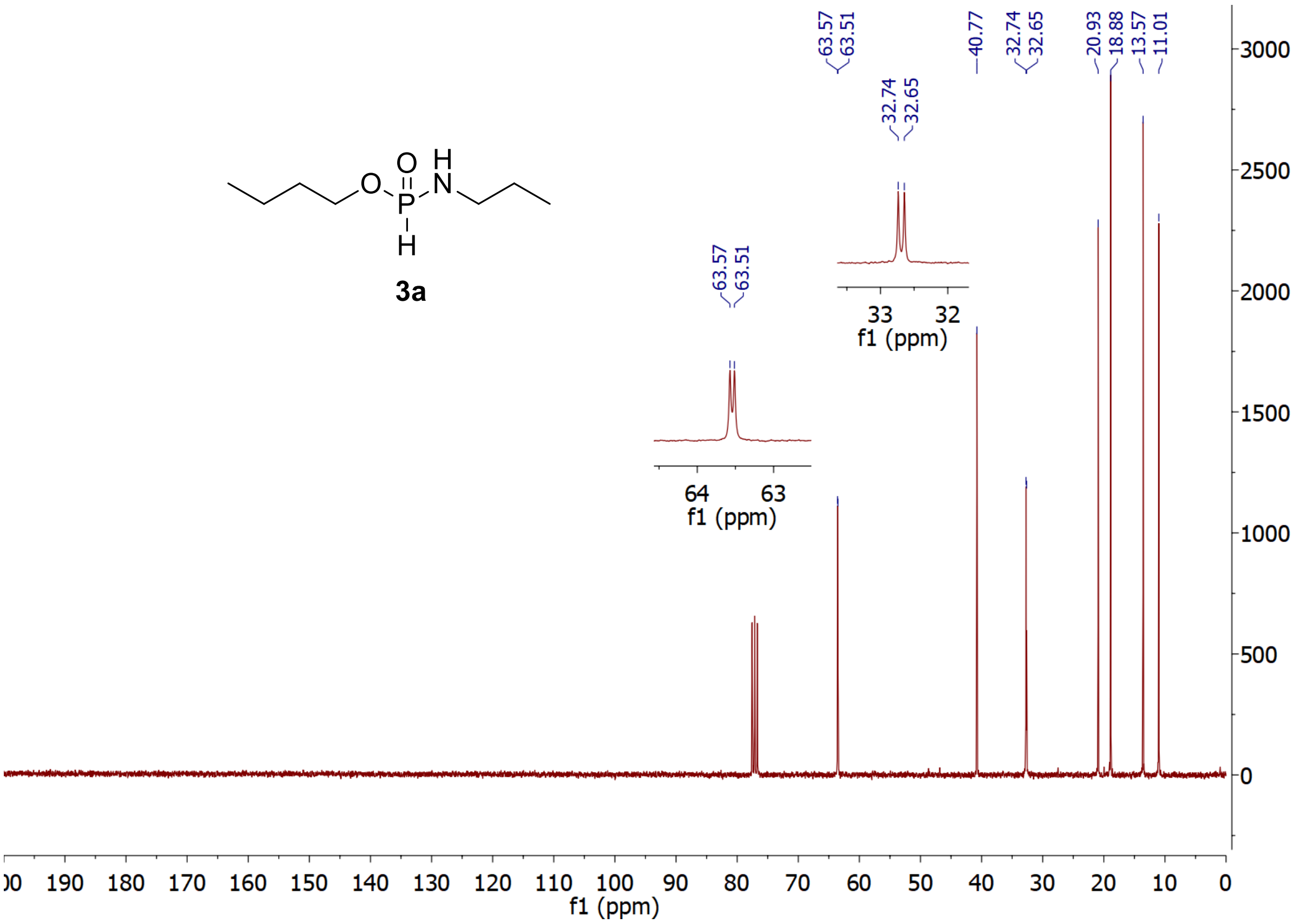

^{1}H NMR (500 MHz, $CDCl_3$) spectra for 3a

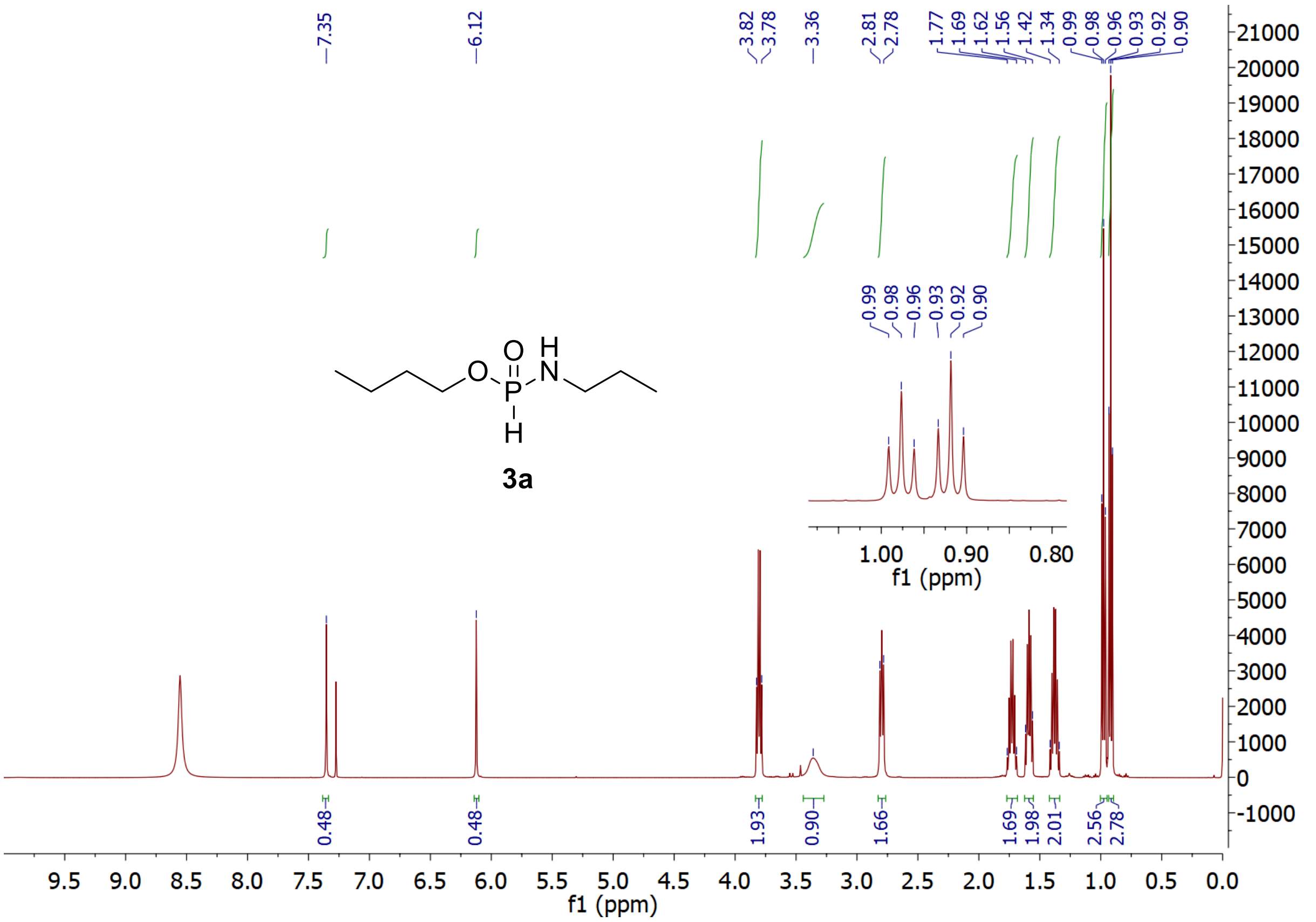

^{31}P {^{1}H} NMR (202 MHz, $CDCl_3$) spectra for 3b

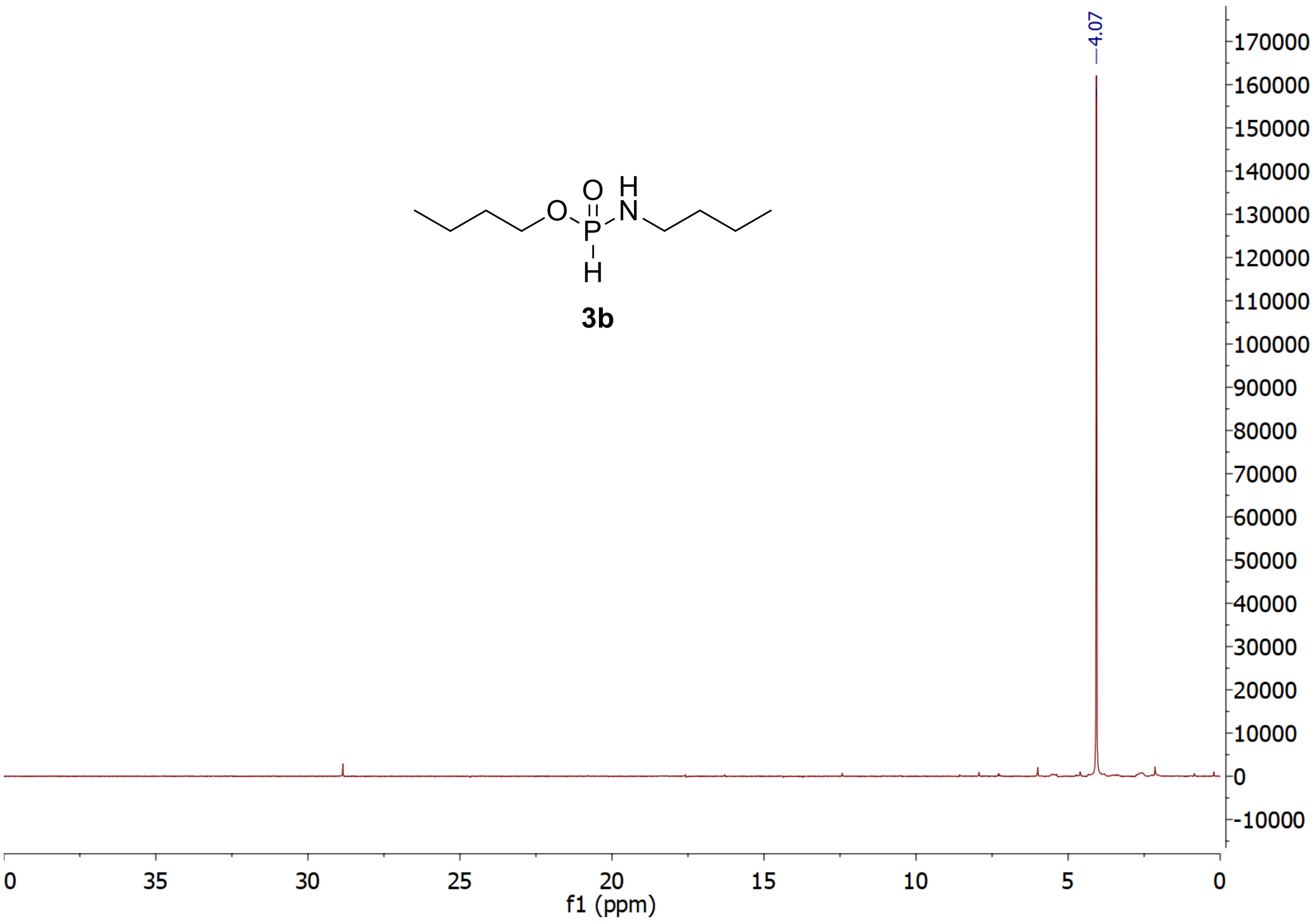

^{13}C {^{1}H} NMR (126 MHz, $CDCl_3$) spectra for 3b

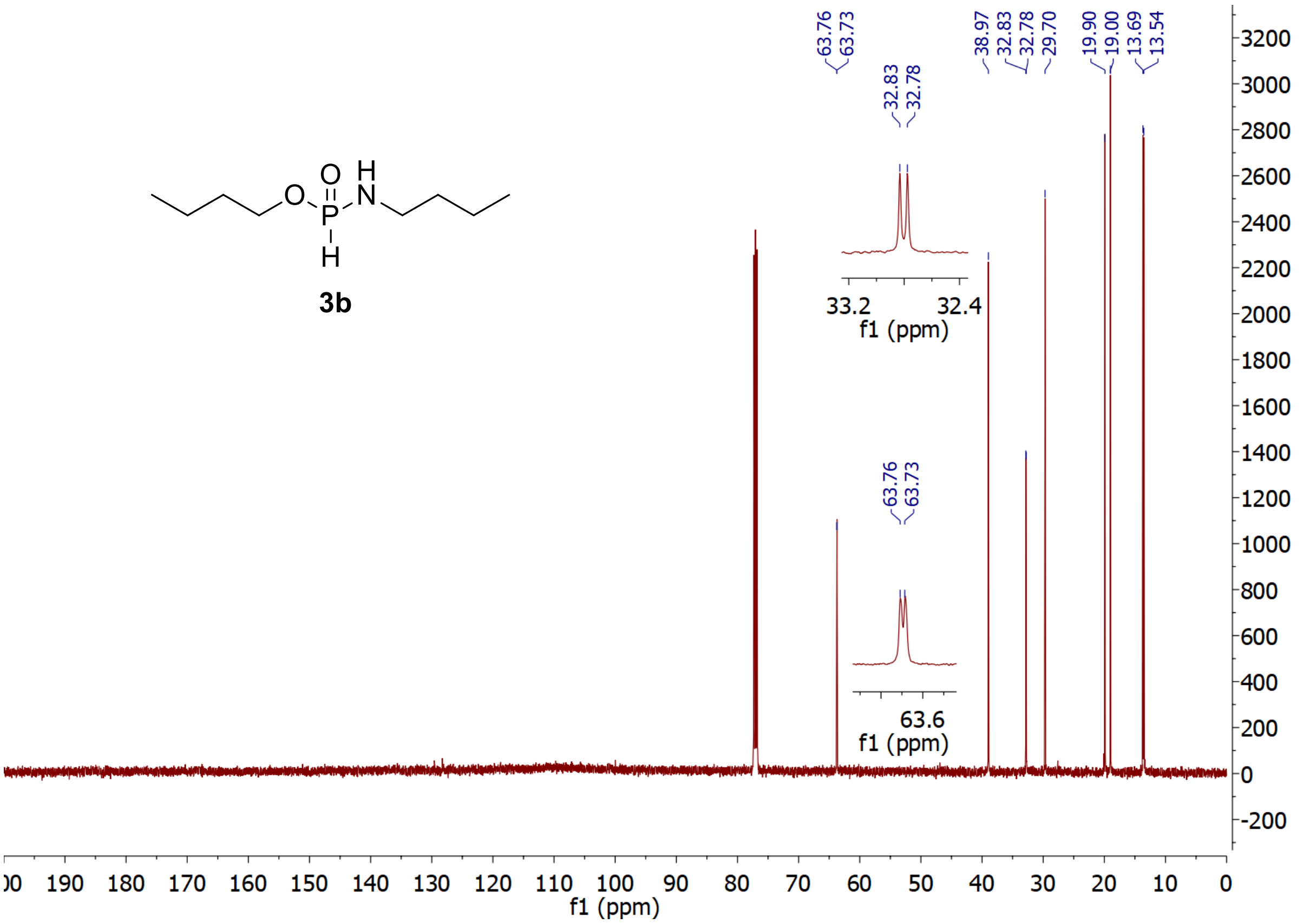

^{1}H NMR (500 MHz, $CDCl_3$) spectra for 3b

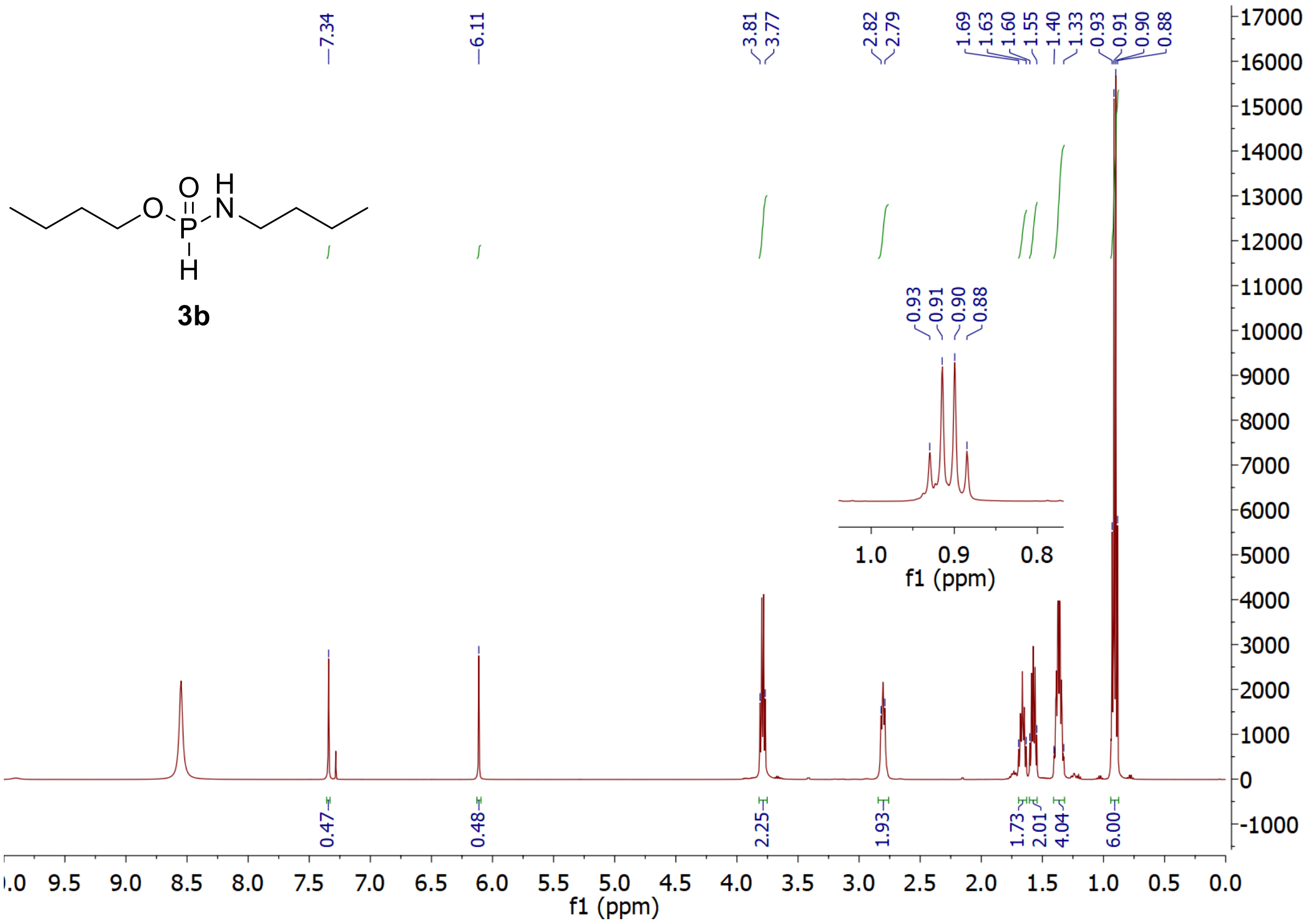

^{31}P {^{1}H} NMR (202 MHz, $CDCl_3$) spectra for 3c

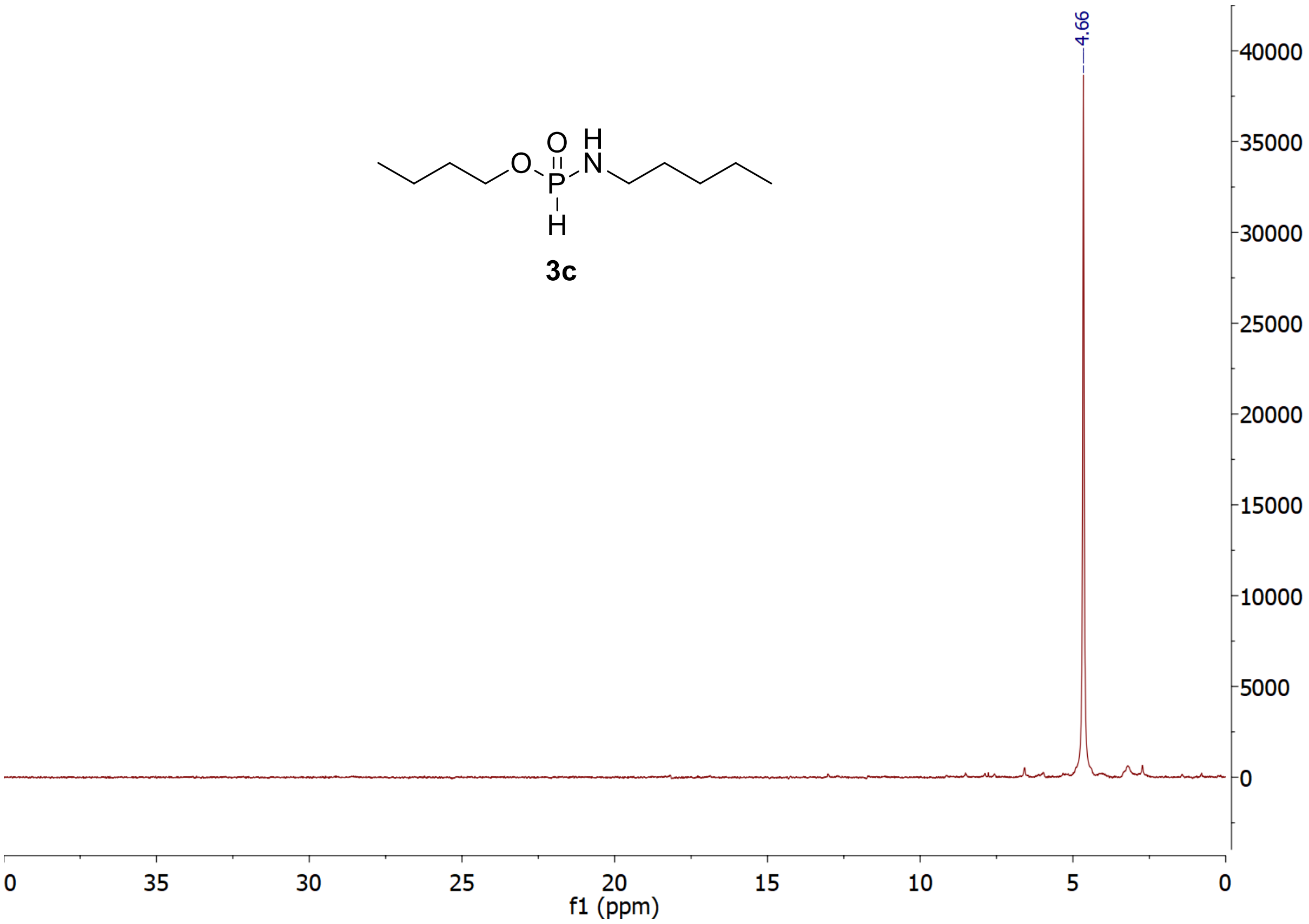

^{13}C {^{1}H} NMR (126 MHz, $CDCl_3$) spectra for 3c

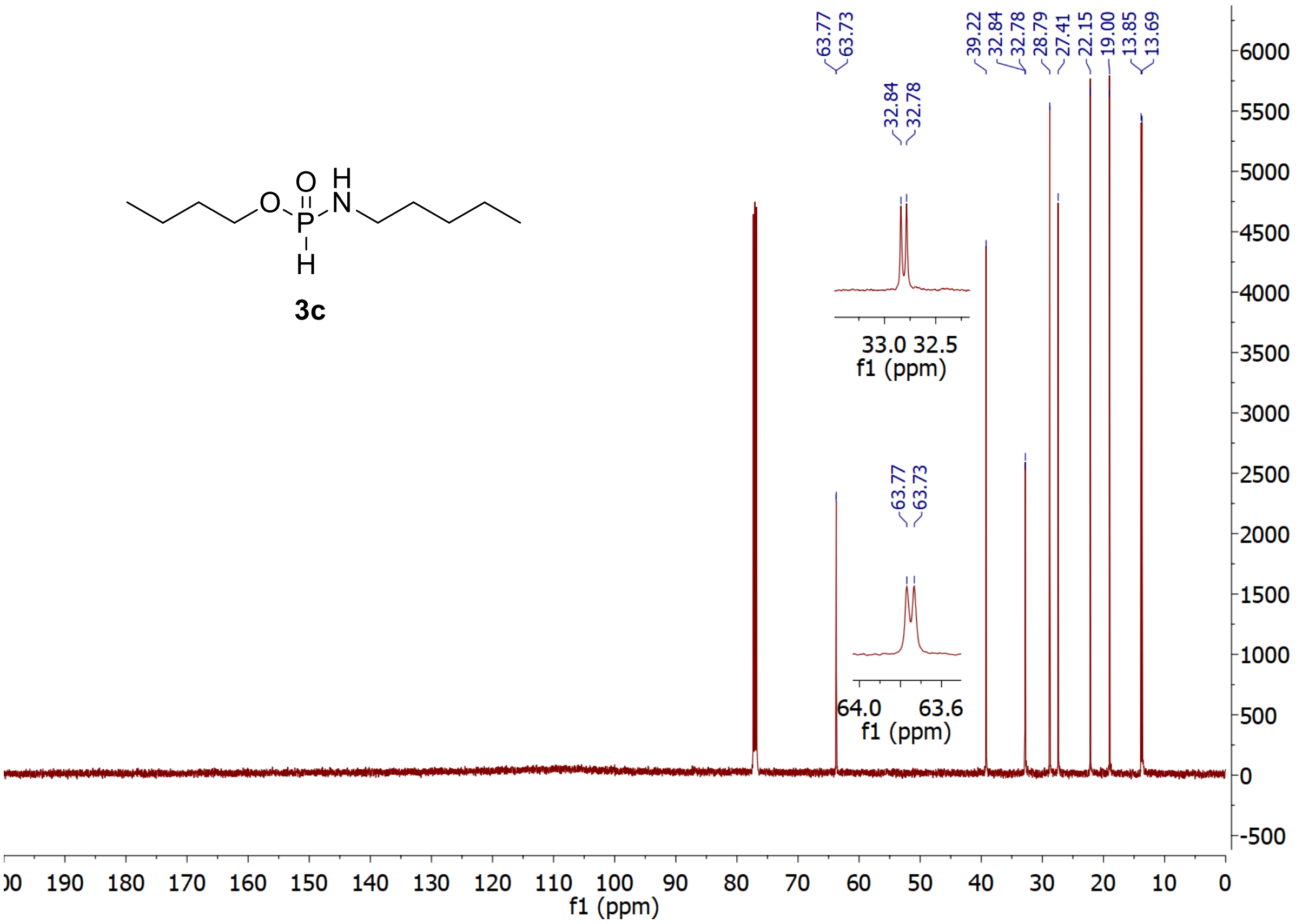

^{1}H NMR (500 MHz, $CDCl_3$) spectra for 3c

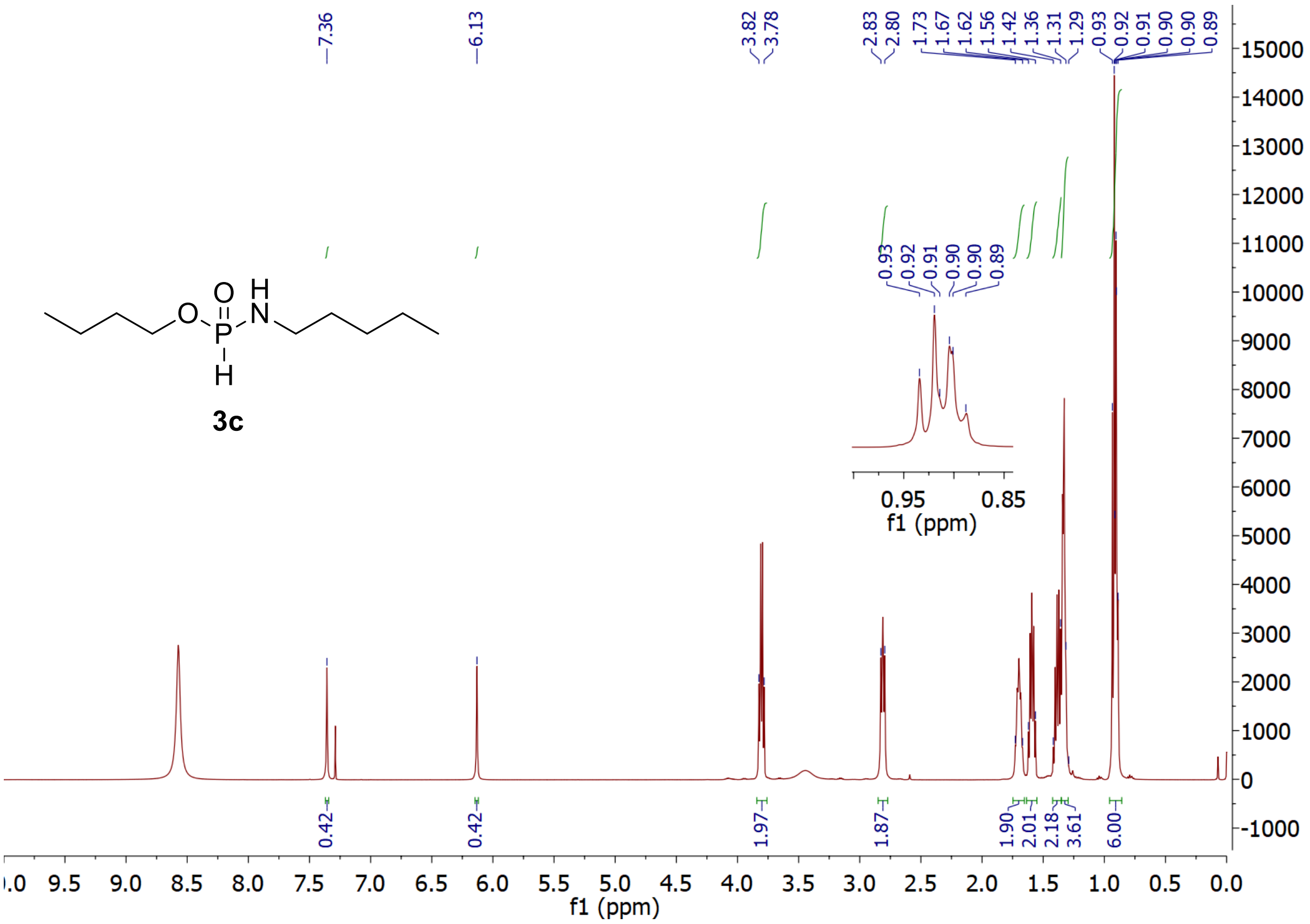

^{31}P {^{1}H} NMR (202 MHz, $CDCl_3$) spectra for 3d

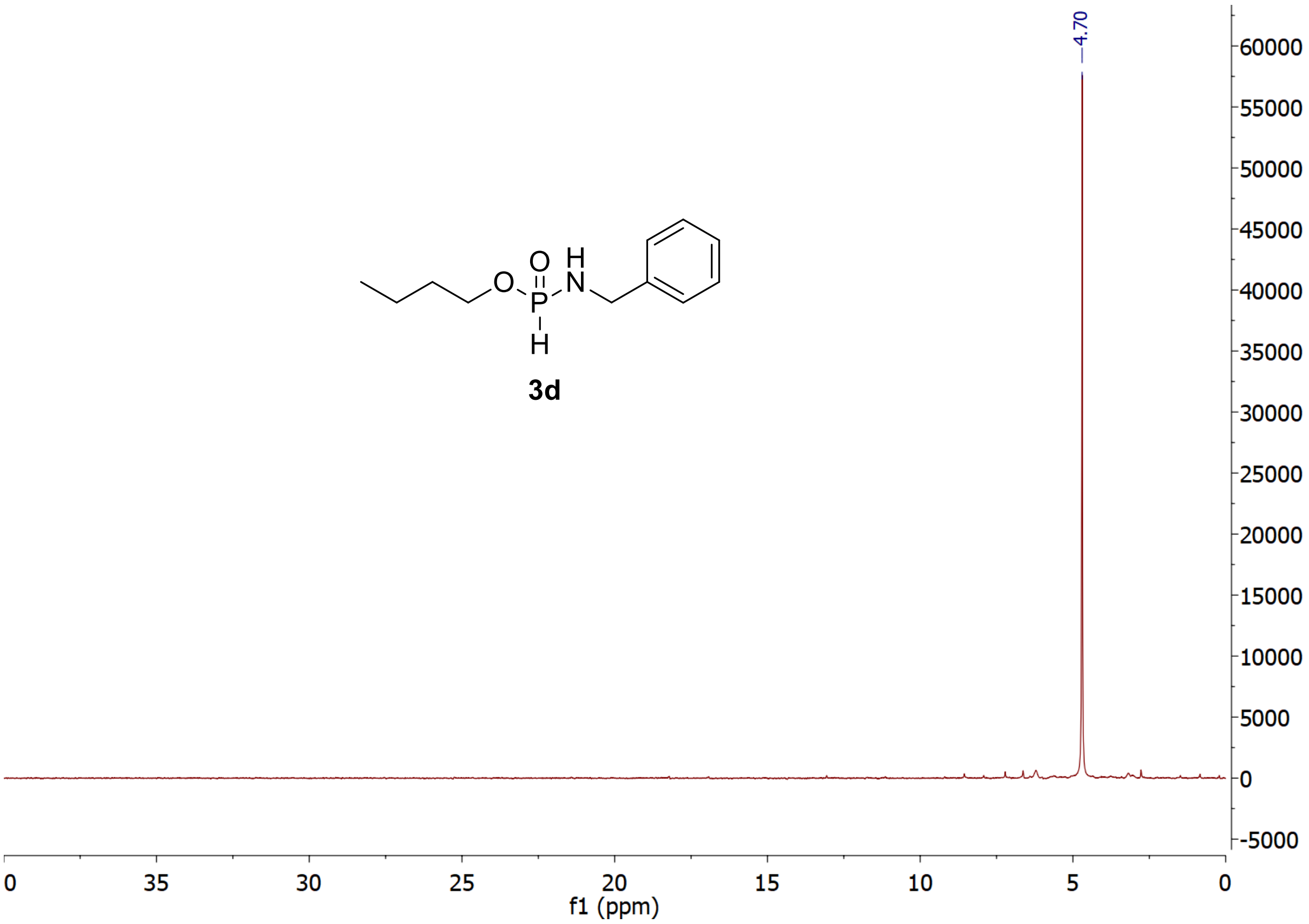

$^{13}C\ \{^1H\}$ NMR (126 MHz, $CDCl_3$) spectra for 3d

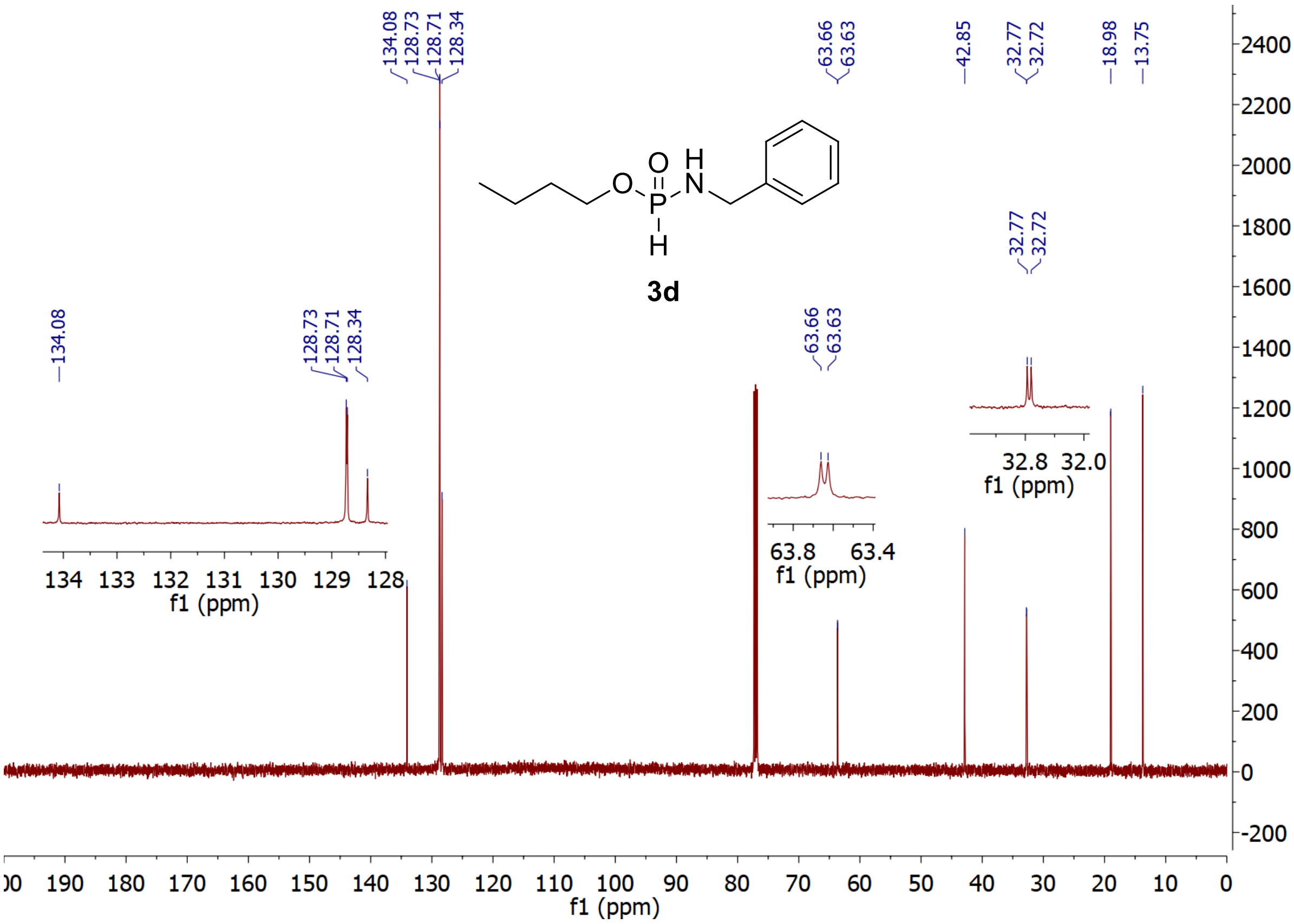

^1H NMR (500 MHz, $CDCl_3$) spectra for 3d

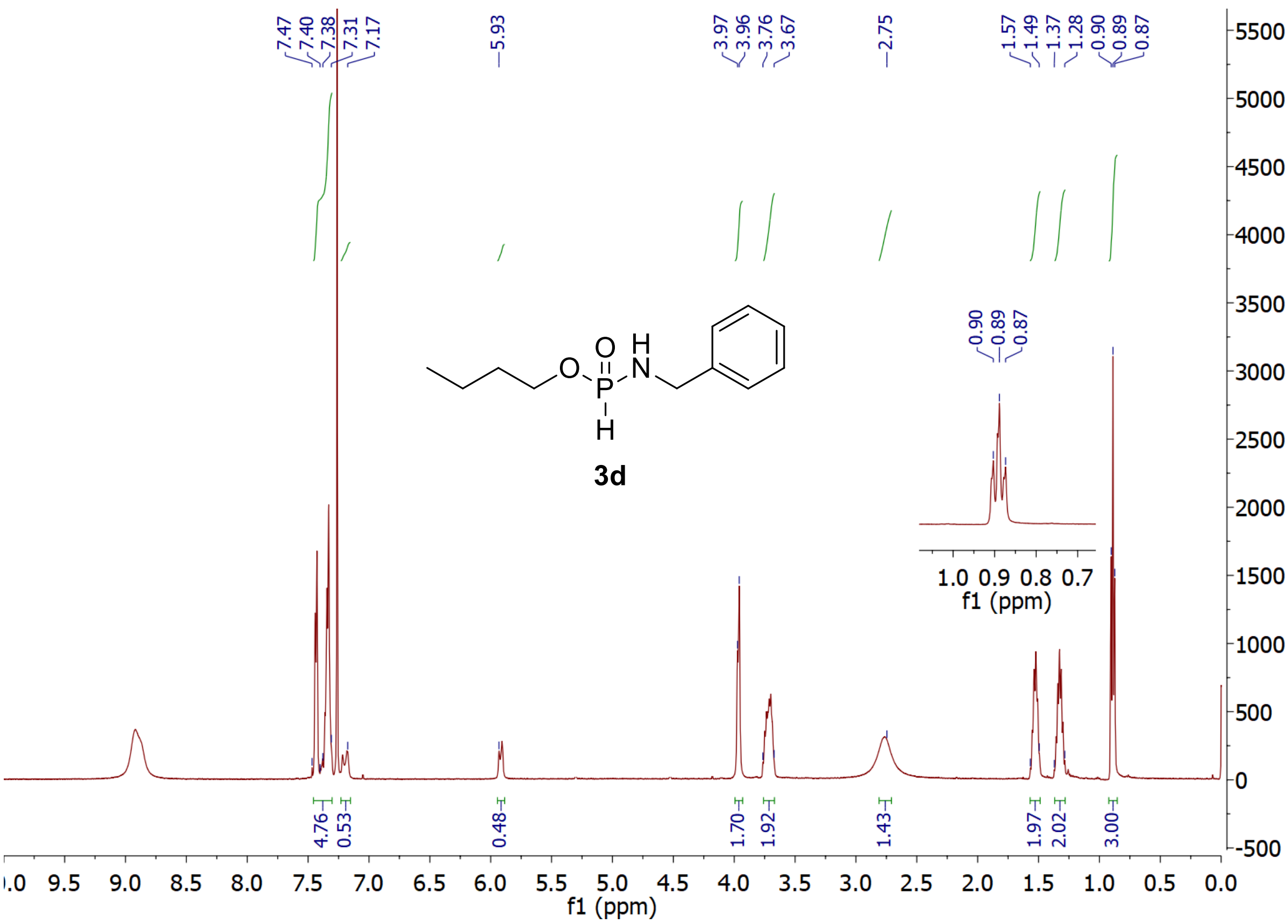

^{31}P {^{1}H} NMR (122 MHz, $CDCl_3$) spectra for 5

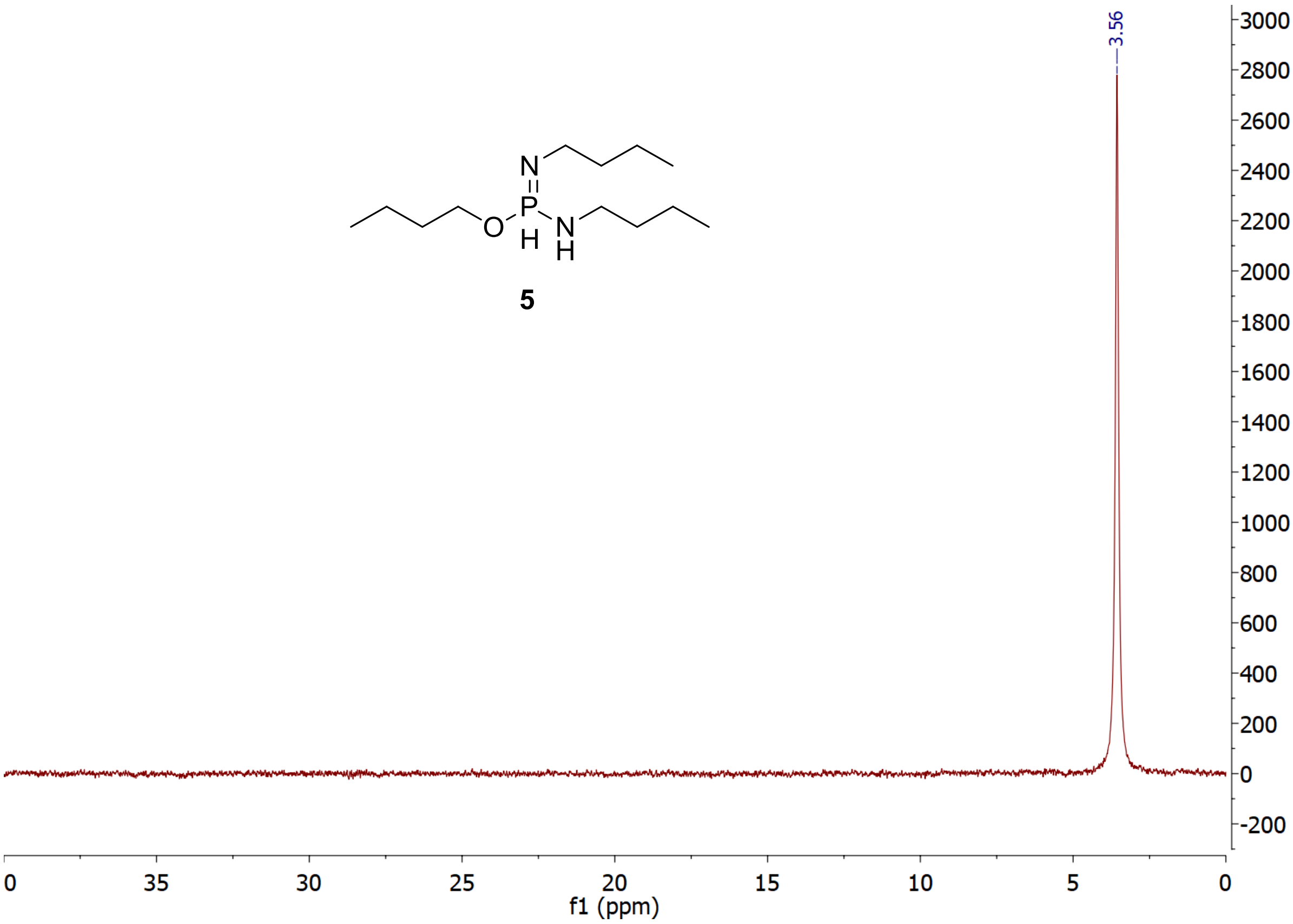

^{13}C {^{1}H} NMR (126 MHz, $CDCl_3$) spectra for 5

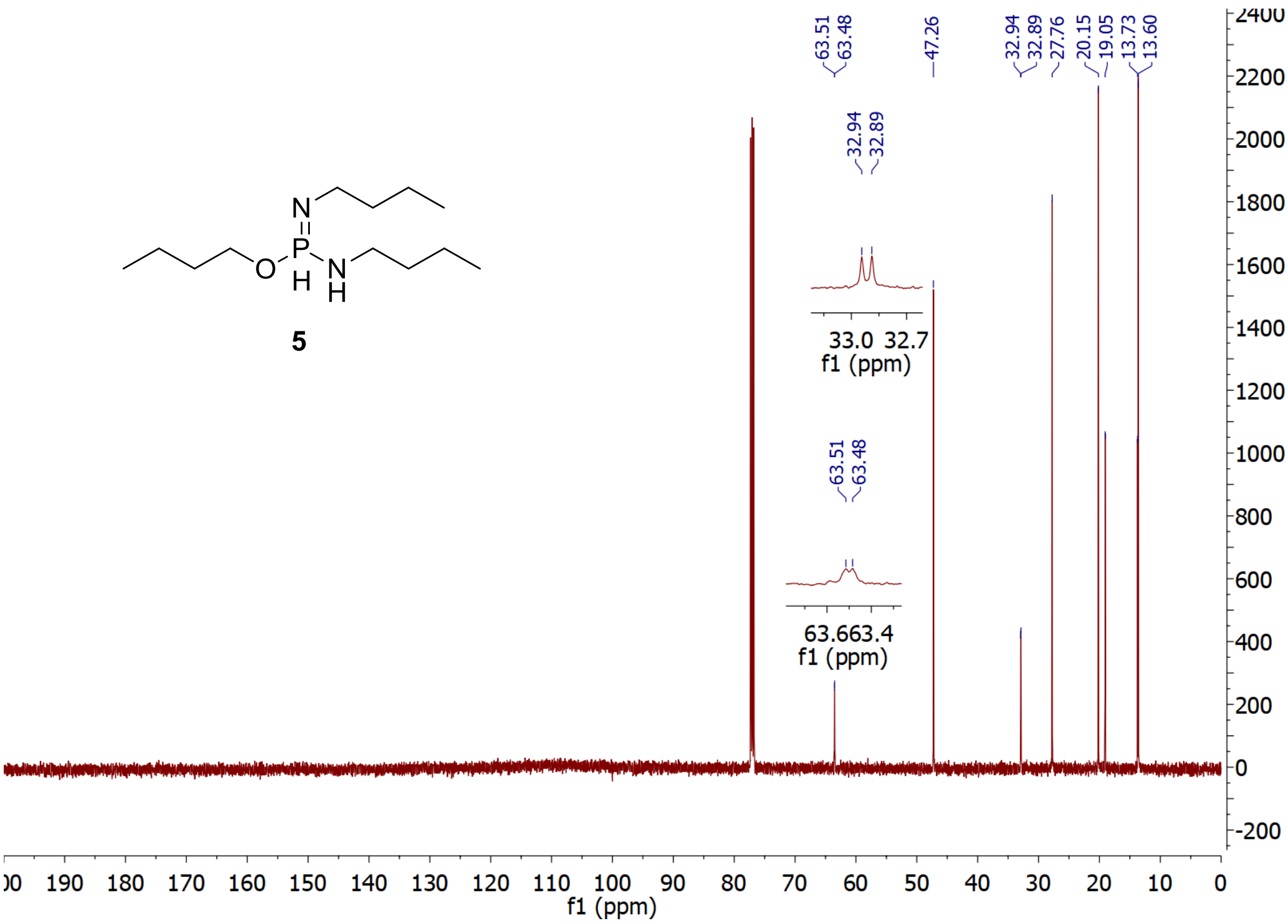

^{1}H NMR (500 MHz, $CDCl_3$) spectra for 5

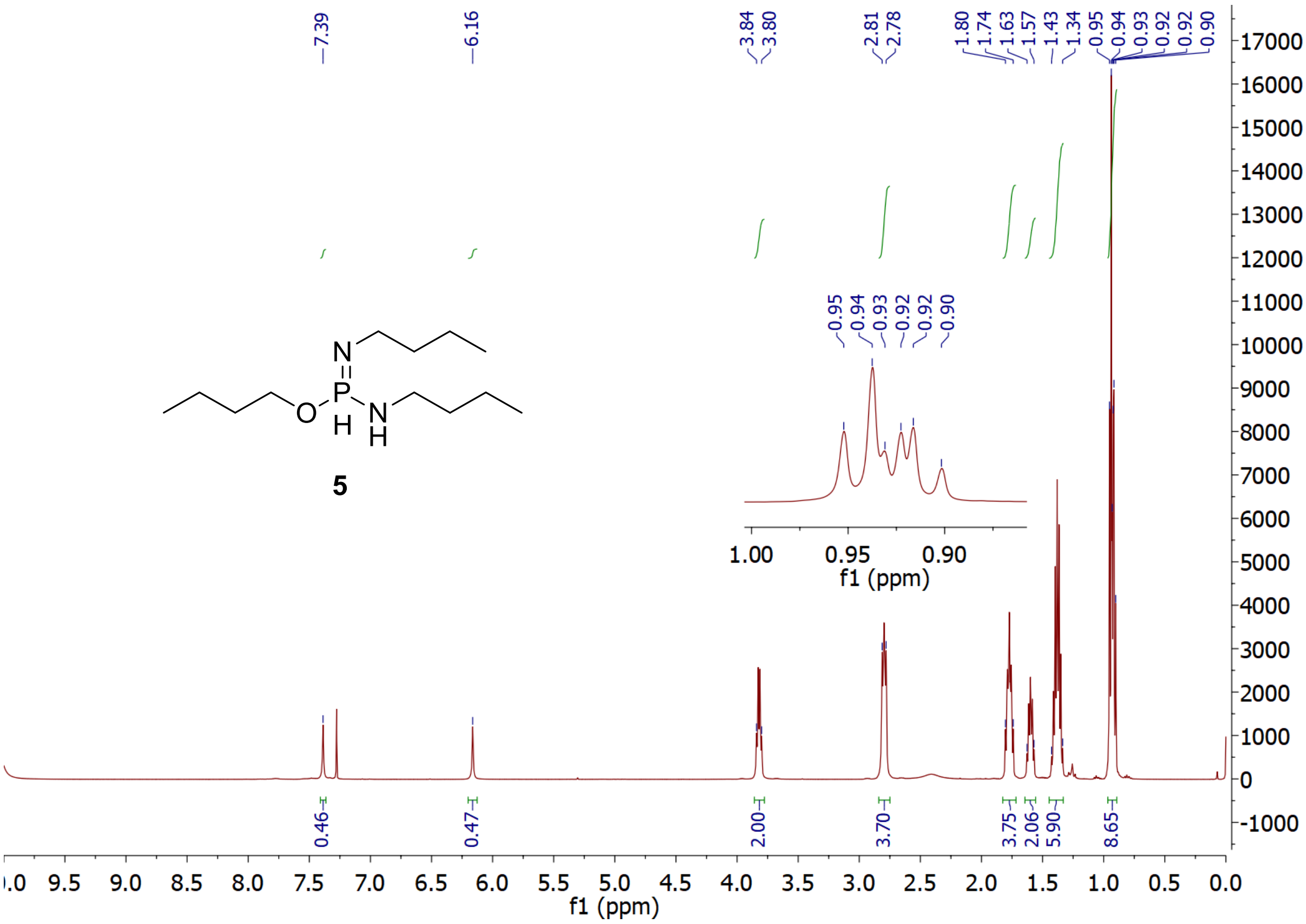

^{31}P {^{1}H} NMR (202 MHz, $CDCl_3$) spectra for 6

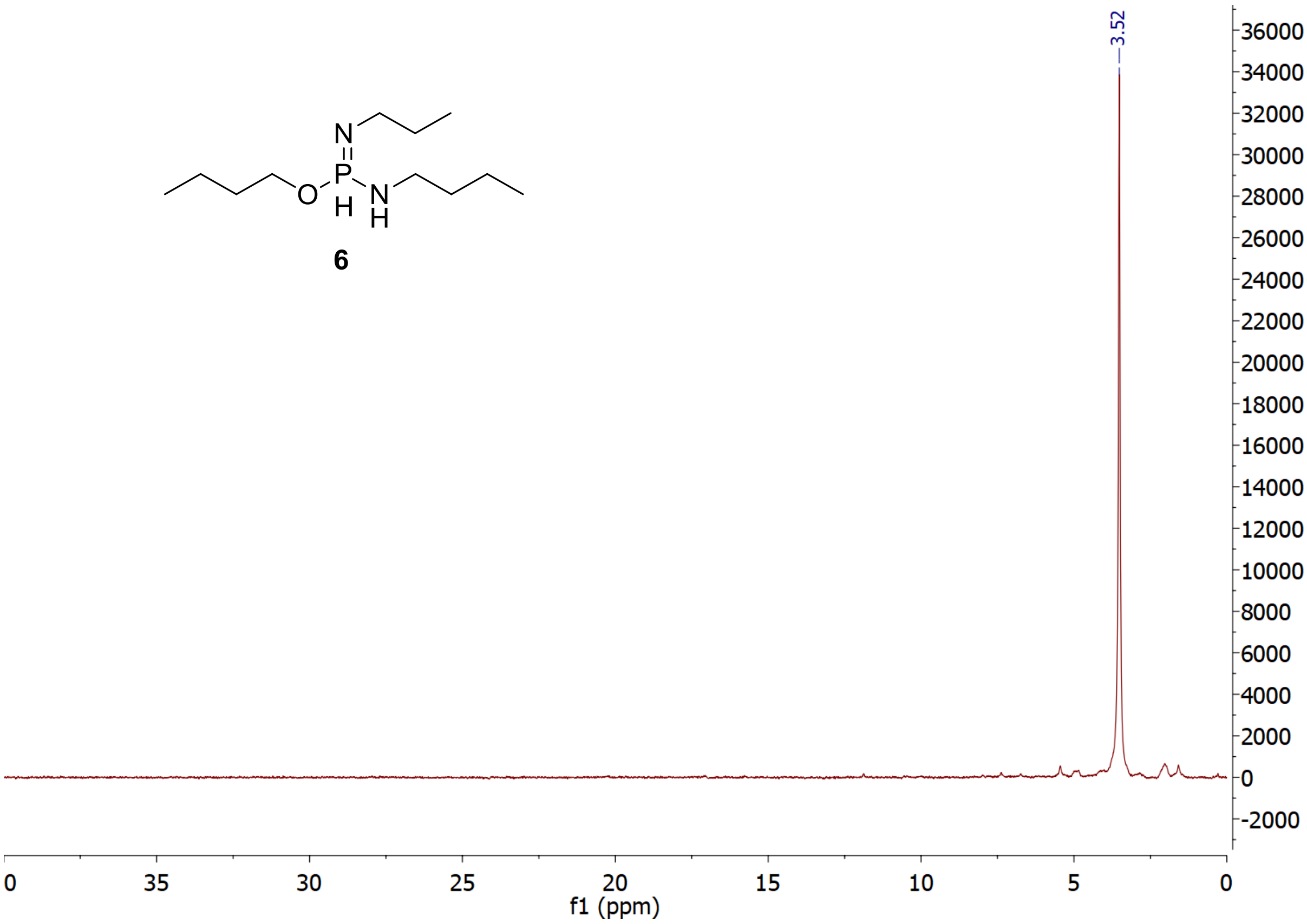

^{13}C {^{1}H} NMR (126 MHz, CDCl3) spectra for 6

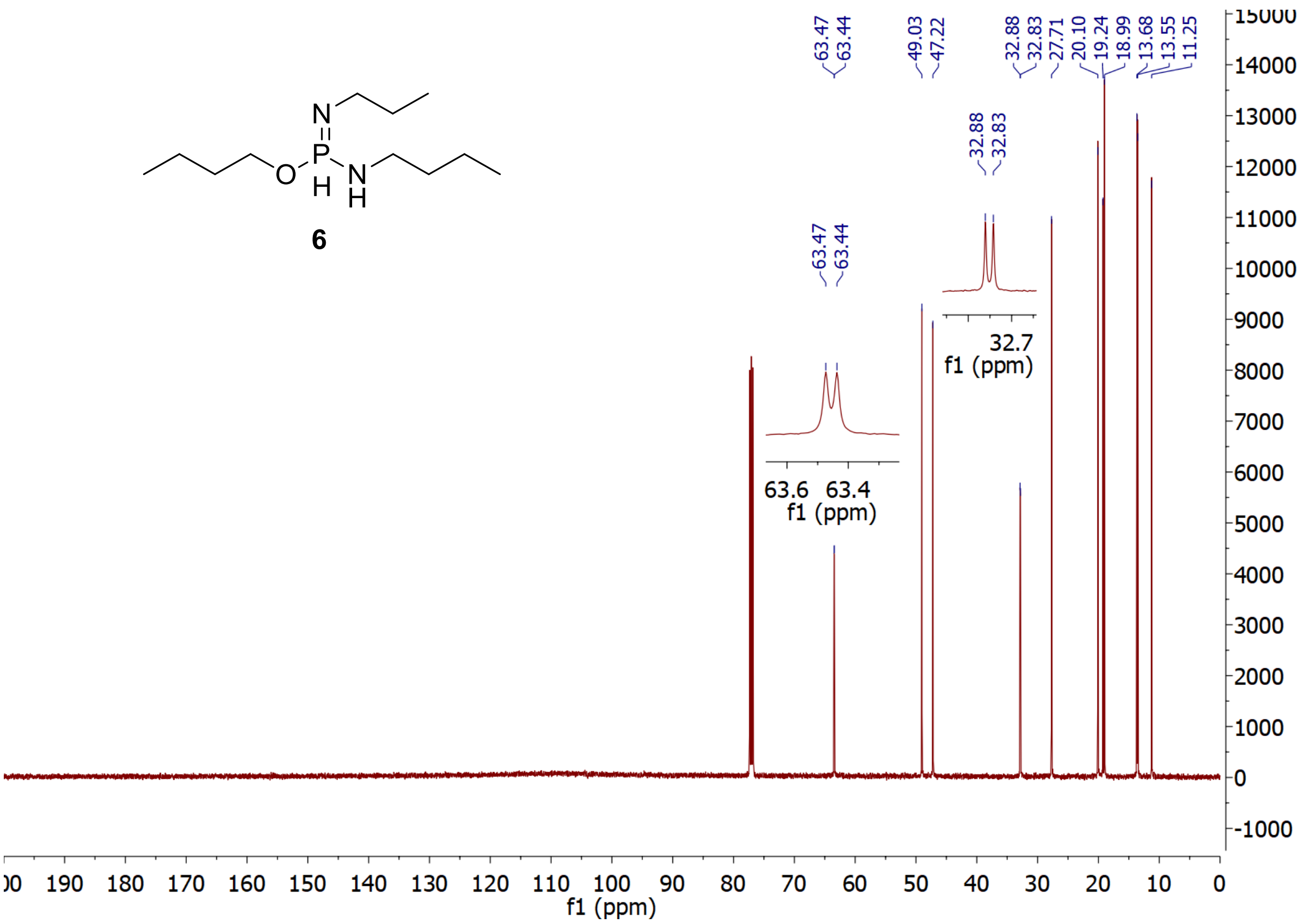

^{1}H NMR (500 MHz, CDCl3) spectra for 6

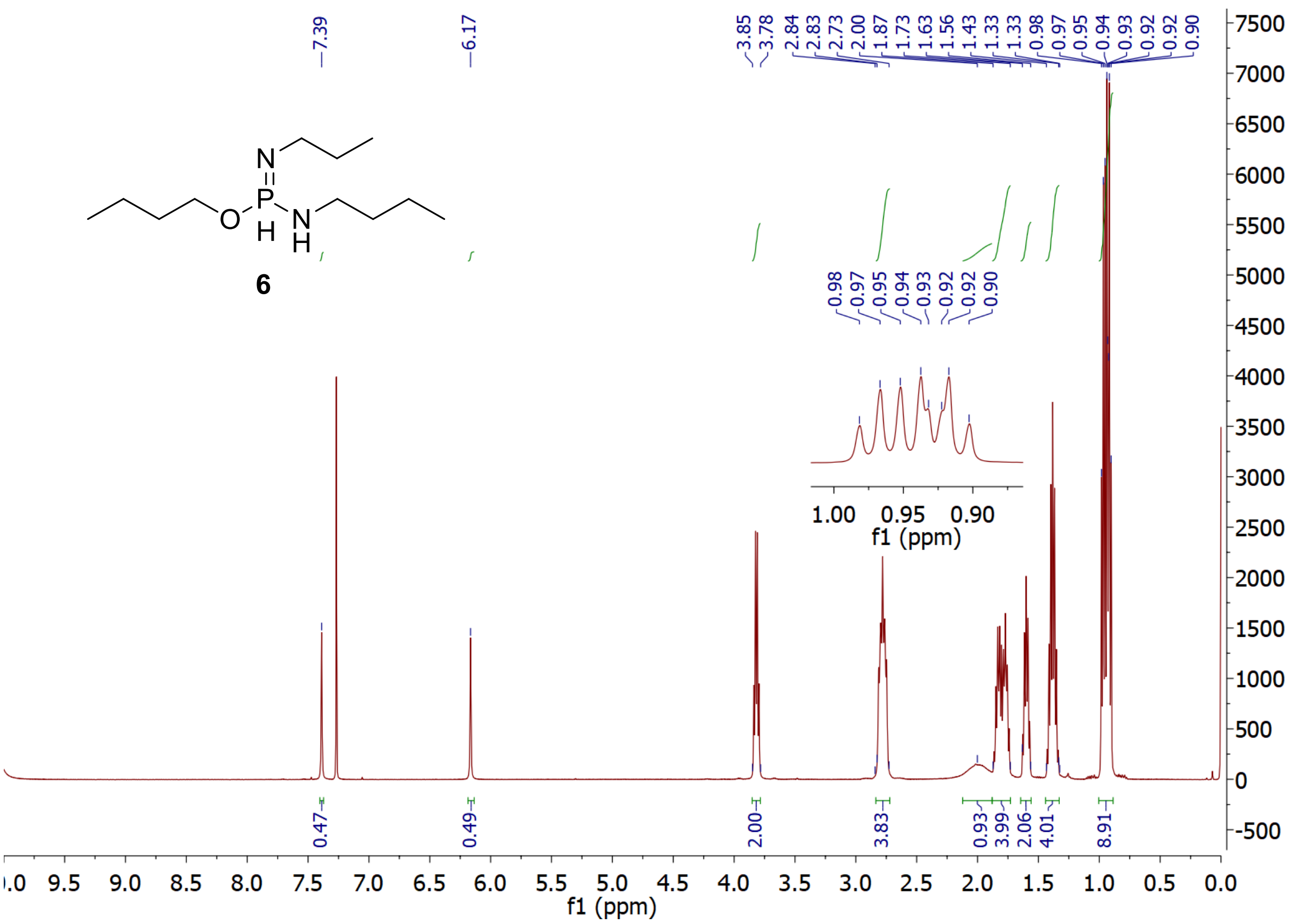

^{31}P {^{1}H} NMR (202 MHz, $CDCl_3$) spectra for 7

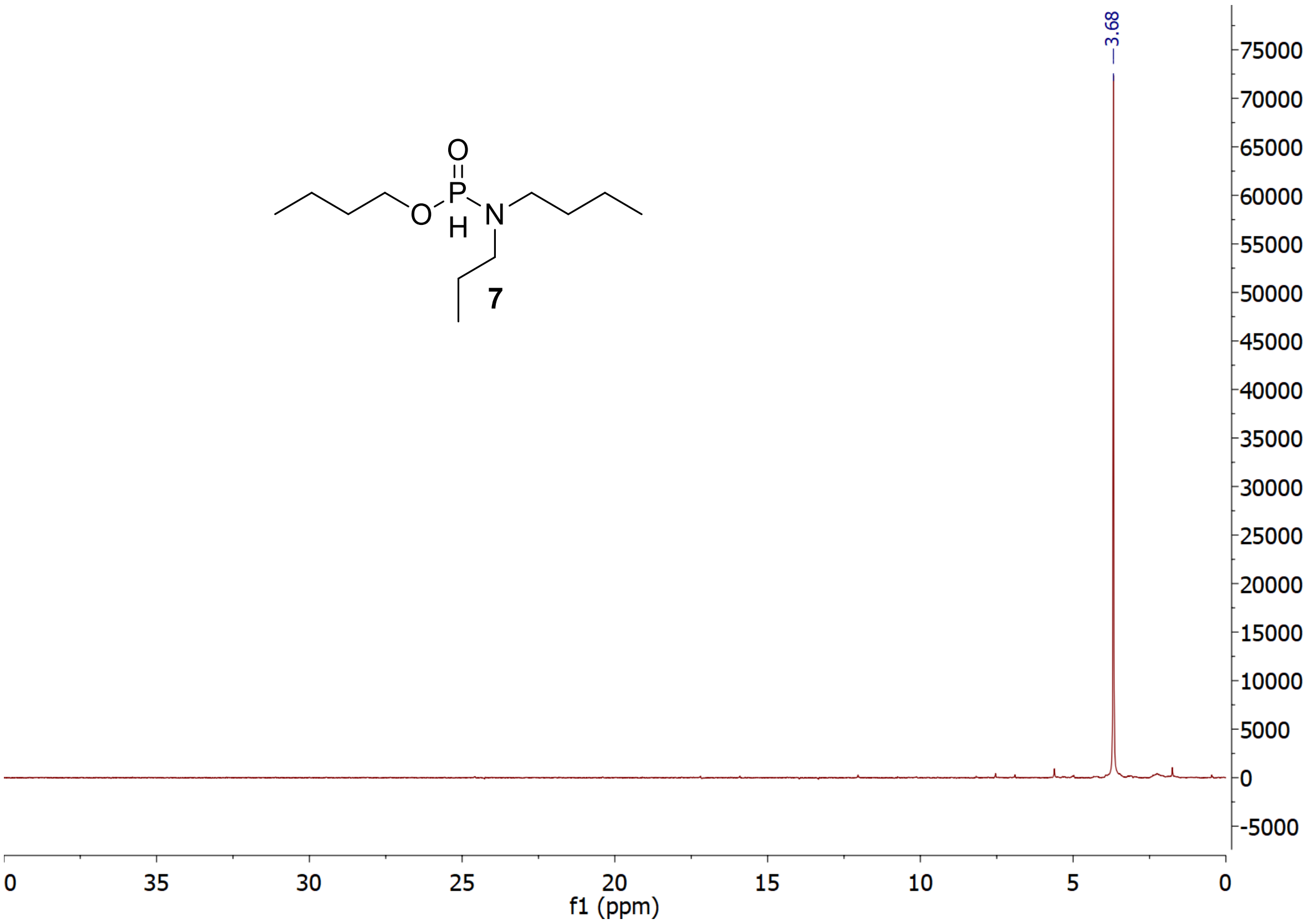

^{13}C {^{1}H} NMR (126 MHz, $CDCl_3$) spectra for 7

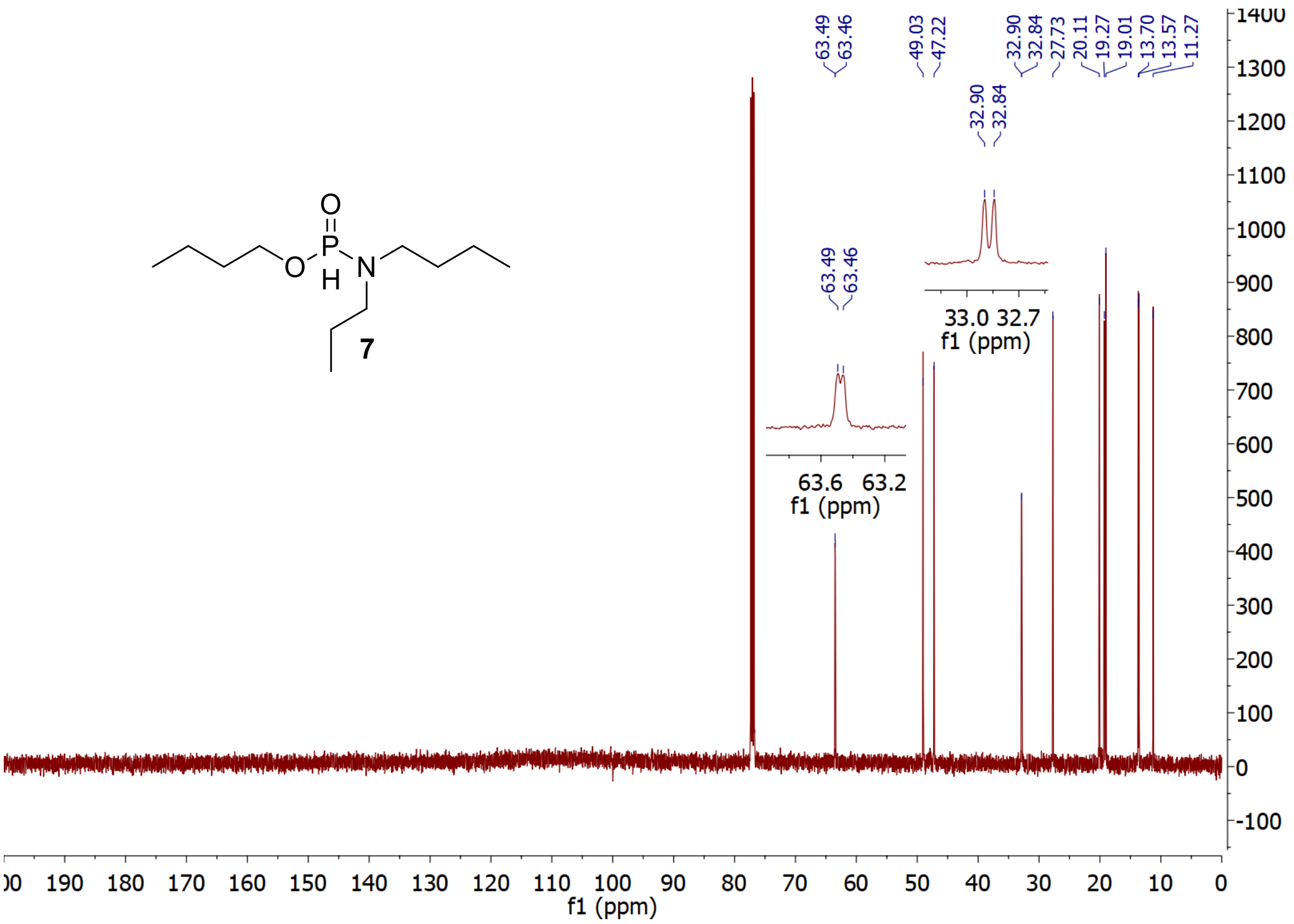

^{1}H NMR (500 MHz, $CDCl_3$) spectra for 7

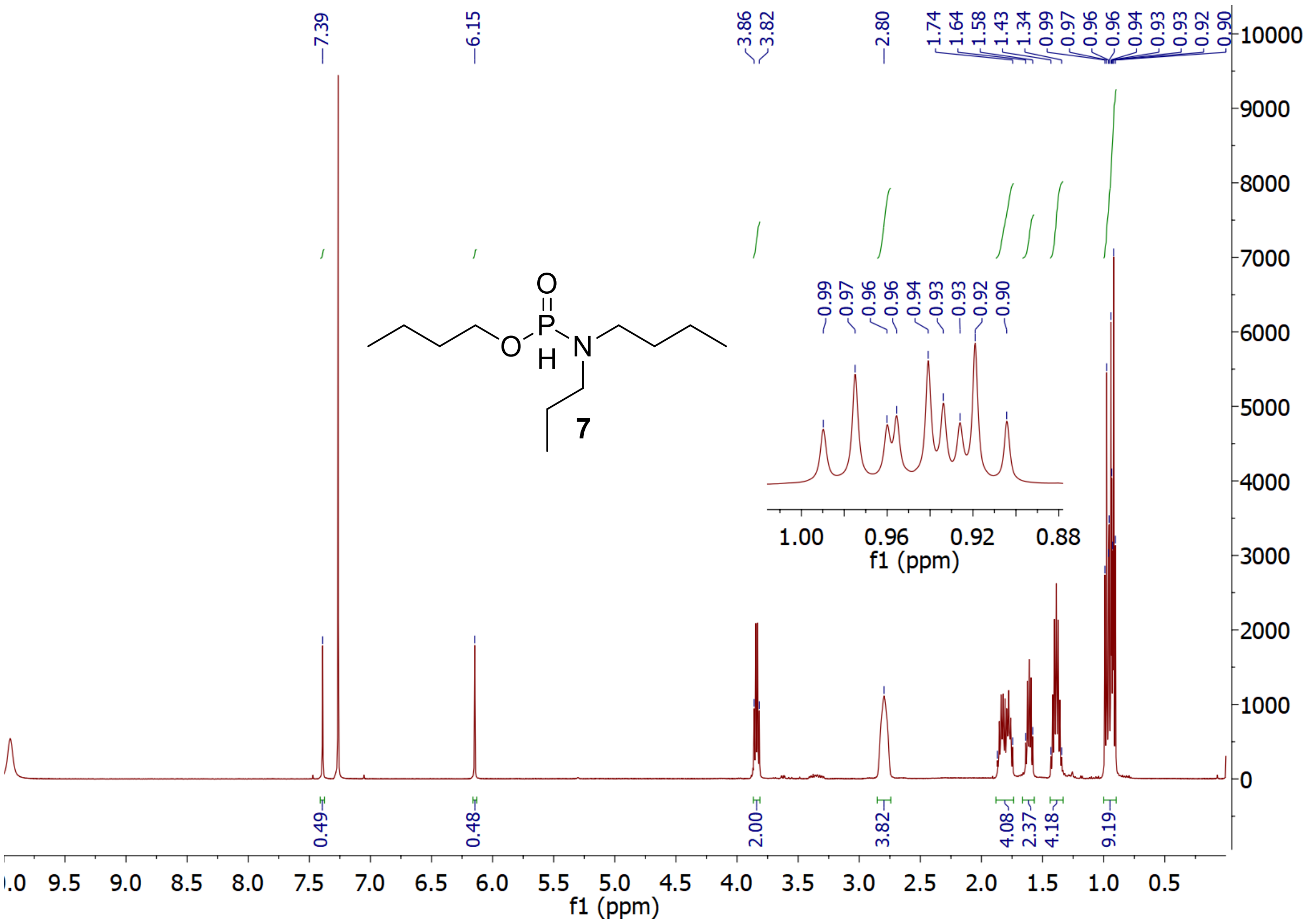

Supplement: Supplementary file 1 [file ol6c03484_si_001.pdf]
